# Supplementary material for: Stepped-wedge cluster-randomised controlled trial to assess the cardiovascular health effects of a managed aquifer recharge initiative to reduce drinking water salinity in southwest coastal Bangladesh: study design and rationale
Source: BMJ Open. 2017 Sep 1;7(9):e015205. doi: 10.1136/bmjopen-2016-015205 (PMC5588995; doi:10.1136/bmjopen-2016-015205)
Supplement: Supplementary file 1 [file bmjopen-2016-015205supp001.pdf]

Supplemental Information 1A: Consent from household head

Protocol Title: Health impacts of a climate change adaptation strategy to address drinking-water salinity in coastal Bangladesh

Investigator's name: Dr. Md. Mahbubur Rahman

Organization: **International Centre for Diarrhoeal Disease Research, Bangladesh (ICDDR,B)**

Purpose of the research

Hello (Assalamualaikum/Nomoshkar). My name is ________ and I work with the ICDDR,B (Cholera Hospital) in Dhaka. You are aware that a Managed aquifer recharge scheme has already been installed in your community that would like reduce the salinity problem in your drinking water. You may also know that drinking saline water is associated with hypertension. We will conduct a pilot study to understand the mean blood pressure of the household members who are drinking water from MAR.

Background (brief introduction of the issue and the need for/ importance of the research)
Salinity in drinking water is a big problem in your community and in many other communities in the south-western coastal region of Bangladesh. Previous research has demonstrated that drinking saline water caused high blood pressure among the adult population and gestational hypertension among the pregnant women. Hypertension risk many other cardiovascular diseases and gestational hypertension also causes adverse pregnancy and fetal outcomes. Managed aquifer recharge (MAR) is a promising sustainable intervention that can reduce salinity in groundwater. Moreover, MAR will not be affected by the cyclone and tidal surges. There is a potential to scale-up MAR across the south-western coastal region if we can demonstrate that drinking MAR water provide health benefits and do not pose any risk to human health. Now we would like to conduct a pilot study where we will measure the mean blood pressure of your household members and the salt concentration of their urine.

Why invited to participate in the study?
You're invited to participate in our study because your household is located in such an area where a new MAR site has been installed. Rain water and pond water is being infiltrated into the groundwater aquifers in this MAR scheme to lower the groundwater salinity. Currently, your household may be using MAR water or other water sources for drinking and cooking purposes. Since your household is among one of the households under the MAR catchment area, we are inviting you to participate in this study.

Methods and procedures
If your household decides to participate, we make two visits to your households. The study duration will be around four months. We will collect information from all the available household members $\geq$20 years old. In each visit, we will measure the blood pressure of all the $\geq$20 years household members and may also measure their 24 hour ambulatory arterial blood pressure by strapping a device in their arms for next 24 hours. We will collect about 20 ml urine

from all the household members as spot urine and will provide a large bag to collect the total amount of urine during the next 24 hours time. We will measure the total protein, creatinine, in urine that will provide important information about your kidney health. In the first visit, the research staff will collect the demographic and socio-economic information, smoking and diseases history, and measure height of available household members. We will also collect 5 ml blood from the participant to assess the genetic marker to understand whether their gene indicates that reduction of salt intake can reduce blood pressure of your family members. Samples will be stored for up to 5 years after the end of the study because we want to take advantage of expected future advances in characterizing genetic markers and other parameters relevant to the salt sensitivity of hypertension. At the end of this time, the samples will be destroyed and a certificate of their destruction will be provided to icddr,b. If you wish to withdraw your samples from the study earlier you may contact the investigators.

In addition, we will collect information about your drinking water sources during each visit and will collect your stored drinking water samples for measuring salinity and sodium concentration in water.

Risk and benefits

There is no more than minimal risk involved in this study. Your household members may feel bit discomfort during blood pressure measurement, blood and urine sample collection, but these will provide important health messages about your household member’s health. Hypertension is very prevalent in Bangladesh and it remains undetected to many people. By participating in this study, your household members will have a screening for hypertension.

There will be also no social harm to you and the rest of the households in this village by your participation in this study. During the visit day, your household need to spend time (~90 minutes) from your daily life for study purposes. There is no direct monetary compensation benefit for participating in this study. However, if drinking MAR water seems to provide health benefits, the study result will help to scale MAR across coastal Bangladesh that many people will have access to safe water with low salinity.

Privacy, anonymity and confidentiality

We will maintain the confidentiality of what information you give us and we will not disclose your identity when we write our reports. We will only use the collective information for the purpose of this study, and we will not use your name in sharing and publishing the results of this study. We expect the steps we take will keep all of your information confidential.

Future use of information

The information collected from this study may be shared with other researchers if needed, but we will strictly maintain your confidentiality and privacy. In the future, we may wish to perform additional tests on the urine and blood sample that will be collected. The samples will be stored at icddr,b.

Right not to participate and withdraw

Taking part in the study is completely voluntary. You may choose not to answer any or all of the questions that will be asked about your household or your behavior. You can drop out of this

study at any time during the interview or anytime during the study period. You have the right to refuse participation in this study.

Principle of compensation

You need not to pay us to take part in this study, and similarly we will not pay you money for attending in the study. You will also be provided best possible, free treatment, for research related injuries.

[Note: Payment for loss of earning of the study participants may be considered in case the participants require extended hospitalization or confinement only for the purpose of the research and/or reimbursement of cost of transportation for participating in the study. However, the amount should be equivalent to the loss and not so high that the offer might induce (influence/bias judgment) participation in the study.

Answering your questions/ Contact persons

If you have any question, you can ask me any time. If you have additional questions about the survey, you may contact: Dr. Solaiman Doza ,Infectious Disease Division, icddr,b, Mohakhali, Dhaka 1212. Phone: +880-2-9827001-10, ext. 4010

If you have questions about your rights as a participant of this research study, or if you think some harm has been done to you because of the survey, you may contact the ICDDR,B IRB: M. A. Salam Khan, IRB Secretariat, phone: 9827084 or PABX 9827001-10 ext. 3206

If you agree to our proposal of enrolling you/your patient in our study, please indicate that by putting your signature or your left thumb impression at the specified space below

Thank you for your cooperation

______________________________________ ____________________

Signature or left thumb impression Date

______________________________________ ____________________

Signature or left thumb impression of the witness Date

______________________________________ ____________________

Signature of the PI or his/her representative Date

(NOTE: In case of representative of the PI, she/he shall put her/his full name and designation and then sign)

Supplemental Information 1B: Consent from $\geq$20 year household members

Protocol Title: Health impacts of a climate change adaptation strategy to address drinking-water salinity in coastal Bangladesh

Investigator's name: Dr. Md. Mahbubur Rahman

Organization: **International Centre for Diarrhoeal Disease Research, Bangladesh (ICDDR,B)**

Purpose of the research

Hello (Assalamualaikum/Nomoshkar). My name is **________** and I work with the ICDDR,B (Cholera Hospital) in Dhaka. You are aware that a Managed aquifer recharge scheme has already been installed in your community that would like reduce the salinity problem in your drinking water. You may also know that drinking saline water is associated with hypertension. We will conduct a pilot study to understand the mean blood pressure of the household members who are drinking water from MAR .

Background (brief introduction of the issue and the need for/ importance of the research)
Salinity in drinking water is a big problem in your community and in many other communities in the south-western coastal region of Bangladesh. Previous research has demonstrated that drinking saline water caused high blood pressure among the adult population and gestational hypertension among the pregnant women. Hypertension risk many other cardiovascular diseases and gestational hypertension also causes adverse pregnancy and fetal outcomes. Managed aquifer recharge (MAR) is a promising sustainable intervention that can reduce salinity in groundwater. Moreover, MAR will not be affected by the cyclone and tidal surges. There is a potential to scale-up MAR across the south-western coastal region if we can demonstrate that drinking MAR water provide health benefits and do not pose any risk to human health. Now we would like to conduct a pilot study where we will measure your mean blood pressure and the salt concentration of urine.

Why invited to participate in the study?
You're invited to participate in our study because your household is located in such an area where a new MAR site has been installed. Rain water and pond water is being infiltrated into the groundwater aquifers in this MAR scheme to lower the groundwater salinity. Currently, you may be using MAR water or other water sources for drinking and cooking purposes. We are inviting all the household members $\geq$20 years old in this MAR scheme catchment area to routinely measure blood pressure and collect urine samples. Since your age is >20 (or 20) years, your participation is very important for the study.

Methods and procedures
If you decide to participate, we will make two visits to measure your blood pressure, collect urine and blood. We may also measure your 24 hour ambulatory arterial blood pressure by strapping a device in your arm for 24 hours. We will collect about 20 ml urine as spot urine and

will provide a large bag to collect the total amount of urine during the 24 hours time. We will measure the total protein, creatinine, in urine that will provide important information about your kidney health. In the first visit, the research staff will collect the demographic and socio-economic information, smoking and diseases history, and measure height of available household members. We will also collect 5 ml of your blood to assess the genetic marker to understand whether your gene indicates that reduction of salt intake can reduce blood pressure of your family members.

So, if you agree, we may also invite you for some further discussions. We would like to record this sessions in a tape recorder as it is very difficult to write all the conversation in a note pad. We would also like to take some notes from this session. This session will last about an hour and half.

Risk and benefits

There is no more than minimal risk involved in this study. You may feel bit discomfort during blood pressure measurement, and urine sample collection, but these will provide important health messages about your health. Hypertension is very prevalent in Bangladesh and it remains undetected to many people. By participating in this study, you will be screened for hypertension.

There will be also no social harm to you and the rest of the households in this village by your participation in this study. During the visit day, you may need to spend time (~90 minutes) from your daily life for study purposes. There is no direct monetary compensation benefit for participating in this study. However, if drinking MAR water seems to provide health benefits, the study result will help to scale MAR across coastal Bangladesh that many people will have access to safe water with low salinity.

Privacy, anonymity and confidentiality

We will maintain the confidentiality of what information you give us and we will not disclose your identity when we write our reports. We will only use the collective information for the purpose of this study, and we will not use your name in sharing and publishing the results of this study. We expect the steps we take will keep all of your information confidential.

Future use of information

The information collected from this study may be shared with other researchers if needed, but we will strictly maintain your confidentiality and privacy. In the future, we may wish to perform additional tests on the urine sample that will be collected. The samples will be stored at icddr,b.

Right not to participate and withdraw

Taking part in the study is completely voluntary. You may choose not to answer any or all of the questions that will be asked about your household or your behavior. You can drop out of this study at any time during the interview or anytime during the study period. You have the right to refuse participation in this study.

Principle of compensation

You need not to pay us to take part in this study, and similarly we will not pay you money for attending in the study. You will also be provided best possible, free treatment, for research related injuries.

[Note: Payment for loss of earning of the study participants may be considered in case the participants require extended hospitalization or confinement only for the purpose of the research and/or reimbursement of cost of transportation for participating in the study. However, the amount should be equivalent to the loss and not so high that the offer might induce (influence/bias judgment) participation in the study.

Answering your questions/ Contact persons

If you have any question, you can ask me any time. If you have additional questions about the survey, you may contact: Dr. Solaiman Doza ,Infectious Disease Division, icddr,b, Mohakhali, Dhaka 1212. Phone: +880-2-9827001-10, ext. 4010

If you have questions about your rights as a participant of this research study, or if you think some harm has been done to you because of the survey, you may contact the ICDDR,B IRB: M. A. Salam Khan, IRB Secretariat, phone: 9827084 or PABX 9827001-10 ext. 3206

If you agree to our proposal of enrolling you/your patient in our study, please indicate that by putting your signature or your left thumb impression at the specified space below

Thank you for your cooperation

______________________________________ ____________________

Signature or left thumb impression Date

______________________________________ ____________________

Signature or left thumb impression of the witness Date

______________________________________ ____________________

Signature of the PI or his/her representative Date

(NOTE: In case of representative of the PI, she/he shall put her/his full name and designation and then sign
