# Supplementary material for: Stepped-wedge cluster-randomised controlled trial to assess the cardiovascular health effects of a managed aquifer recharge initiative to reduce drinking water salinity in southwest coastal Bangladesh: study design and rationale
Source: BMJ Open. 2017 Sep 1;7(9):e015205. doi: 10.1136/bmjopen-2016-015205 (PMC5588995; doi:10.1136/bmjopen-2016-015205)
Supplement: Author's manuscript [file bmjopen-2016-015205.draft_revisions.pdf]

BMJ Open

BMJ Open is committed to open peer review. As part of this commitment we make the peer review history of every article we publish publicly available.

When an article is published we post the peer reviewers' comments and the authors' responses online. We also post the versions of the paper that were used during peer review. These are the versions that the peer review comments apply to.

The versions of the paper that follow are the versions that were submitted during the peer review process. They are not the versions of record or the final published versions. They should not be cited or distributed as the published version of this manuscript.

BMJ Open is an open access journal and the full, final, typeset and author-corrected version of record of the manuscript is available on our site with no access controls, subscription charges or pay-per-view fees (http://bmjopen.bmj.com).

If you have any questions on BMJ Open's open peer review process please email info.bmjopen@bmj.com

BMJ Open

Stepped-wedge cluster-randomized controlled trial to assess the cardiovascular health effects of a managed aquifer recharge initiative to reduce drinking water salinity in southwest coastal Bangladesh: study design and rationale

Journal:	*BMJ Open*
Manuscript ID	bmjopen-2016-015205
Article Type:	Protocol
Date Submitted by the Author:	17-Nov-2016

Complete List of Authors:	Naser, Abu; Emory University, Department of Environmental Health Sciences, Rollins School of Public Health<br>Unicomb, Leanne; International Centre for Diarrhoeal Disease Research, Centre for Communicable Diseases<br>Doza, Solaiman; International Centre for Diarrhoeal Disease Research, Centre for Communicable Diseases<br>Ahmed, Kazi; Dhaka University, Department of Geology<br>Rahman, Mahbubur ; International Centre for Diarrhoeal Disease Research, Centre for Communicable Diseases<br>Uddin, Mohammad; International Centre for Diarrhoeal Disease Research, Centre for Communicable Diseases<br>Quraishi, Shamshad; Atomic Energy Centre, Analytical Chemistry Laboratory<br>Selim, Shahjada; Bangabandhu Sheikh Mujib Medical University<br>Shamsudduha, Mohammad ; University College London, Institute for Risk and Disaster Reduction<br>Burgess, William; University College London, Department of Earth Sciences<br>Chang, Howard; Emory University, Department of Biostatistics and Bioinformatics, Rollins School of Public Health, Emory University<br>Gribble, Matthew; Emory University, Department of Environmental Health Sciences, Rollins School of Public Health<br>Clasen, Thomas; Emory University, Department of Environmental Health Sciences, Rollins School of Public Health<br>Luby, Stephen; International Centre for Diarrhoeal Disease Research, Centre for Communicable Diseases; Stanford University, Woods Institute for the Environment

**Primary Subject Heading**:	Public health
Secondary Subject Heading:	Epidemiology, Global health, Occupational and environmental medicine, Cardiovascular medicine
Keywords:	Salt, Drinking water, Shallow aquifer, stepped-wedge trial, Managed aquifer recharge, Blood pressure

SCHOLARONE™
Manuscripts

For peer review only

Stepped-wedge cluster-randomized controlled trial to assess the cardiovascular health effects of a managed aquifer recharge initiative to reduce drinking water salinity in southwest coastal Bangladesh: study design and rationale

Abu Mohd Naser[1*], Leanne Unicomb[2], Solaiman Doza[2], Kazi Matin Ahmed[3], Mahbubur Rahman[2], Mohammad Nasir Uddin[2], Shamshad B. Quraishi[4], Shahjada Selim[5], Mohammad Shamsudduha[6], William Burgess[7], Howard H. Chang[8], Matthew O. Gribble[1], Thomas F. Clasen[1], Stephen P. Luby[9]

[1] Department of Environmental Health Sciences, Rollins School of Public Health, Emory University, Atlanta, USA

[2]International Centre for Diarrhoeal Disease Research, Bangladesh (icddr,b), GPO Box 128, Dhaka 1000, Bangladesh

[3] Department of Geology, University of Dhaka, Dhaka, Bangladesh

[4]Analytical Chemistry Laboratory, Atomic Energy Centre, Dhaka, Bangladesh

[5]Bangabandhu Sheikh Mujib Medical University, Dhaka, Bangladesh

[6] Institute for Risk and Disaster Reduction, University College London, UK

[7] Department of Earth Sciences, University College London, London, UK

[8] Department of Biostatistics and Bioinformatics, Rollins School of Public Health, Emory University, Atlanta, USA

[9]Woods Institute for the Environment, Stanford University, Stanford, California, USA

*Corresponding author:

Abu Mohd Naser, MBBS, MPH
Department of Environmental Health Sciences
Rollins School of Public Health | Emory University
2nd floor, CNR building, Room # 2030E
Atlanta, GA 30322, USA
email: atitu@emory.edu

For peer review only - http://bmjopen.bmj.com/site/about/guidelines.xhtml

Abstract

Introduction: Saltwater intrusion and salinization have contributed to drinking water scarcity in many coastal regions globally, leading to dependence on alternative sources for water supply. In southwest coastal Bangladesh, communities have few options but to drink brackish groundwater which has been associated with high blood pressure among the adult population, and preeclampsia and gestational hypertension among pregnant women. Managed aquifer recharge (MAR), the purposeful recharge of surface water or rainwater to aquifers to bring hydrologic equilibrium, is a potential solution for salinity problem in southwest coastal Bangladesh by creating a freshwater lens within the brackish aquifer. Our study aims to evaluate whether consumption of MAR water improves human health, particularly by reducing blood pressure among communities in coastal Bangladesh.

Methods and analysis: The study employs a stepped-wedge cluster-randomized controlled community trial design to evaluate the impact of the programmatic implementation of MAR intervention in 16 communities over five monthly visits. During each visit, we will collect data on participants' source of drinking and cooking water and measure the salinity level and electrical conductivity of household stored water. At each visit, we will also measure blood pressure of participants' ≥ 20 years of age and pregnant women and collect urine samples for urinary sodium and protein measurements. We will use generalized estimating equations with robust standard errors to determine the association of access to MAR water on blood pressure of the participants.

Ethics and dissemination: The study protocol has been reviewed and approved by the Institutional Review Boards of the International Centre for Diarrheal Disease Research, Bangladesh (icddr.b). Informed written consent will be taken from all the participants. This study

For peer review only - http://bmjopen.bmj.com/site/about/guidelines.xhtml

is funded by Wellcome Trust, UK. The study findings will be disseminated to the government partners, at research conferences and in peer reviewed journals.

Registration: (http://www.clinicaltrials.gov): NCT02746003

For peer review only

Strengths and limitation of the study

- This is the first study to evaluate the health impact of managed aquifer recharge in southwest coastal Bangladesh.
- The stepped-wedge trial ensures we will have counterfactual data as well as gradual access to MAR water in all communities.
- Objective measurement of exposure (drinking water salinity) and outcomes (urinary sodium and blood pressure).
- The magnitude of exposure will vary geographically and across time period. Therefore, MAR water salinity will differ across communities at a single point of time, and also for the same community at different points of time.
- Compliance of the intervention may be different across sites and for individuals of different professions.

Introduction

Background and rationale

Saltwater intrusion and salinization have increased groundwater salinity in many coastal aquifers and small islands across the world[1-5]. This is driven by a number of climatological and anthropogenic factors including global warming, increased cyclones and tidal surges, reduced river discharge, and increased groundwater abstraction in excess of recharge[6-8]. Communities in many coastal regions rely on groundwater as their main source of drinking water [9] as well as freshwater for domestic, agricultural and industrial purposes [10]. Nearly half of the world's population resides in coastal areas [3] and 10% of these live in low-lying coastal areas where surface elevation is generally ≤10 meters above mean sea level [11]. Salinization in coastal areas is expected to increase in the future because of increased groundwater withdrawal due to population and economic growth and sea-level rise [3]. As the world's population and economic activities continue to grow, groundwater supplies are progressively under threat of depletion, which increases the importance of monitoring, management and conservation of coastal freshwater aquifers [12 13].

One approach to minimize the impact of groundwater salinization is to enhance groundwater recharge into coastal aquifers [14]. Managed aquifer recharge (MAR) is an approach to artificially promote freshwater recharge to increase storage. MAR involves infiltration of freshwater (e.g. rainwater and pond water) into aquifers to create a store of freshwater within the naturally brackish aquifer (**Figure 1**)[15-17]. MAR represents a promising adaptive strategy for increasing freshwater availability and sustaining a year-round drinking water supply that is

For peer review only - http://bmjopen.bmj.com/site/about/guidelines.xhtml

protected from evaporation, and could be resilient to tidal storms, cyclones and surface water salinity since freshwater infiltration and storage occur under confined conditions [18].

The brackishness of groundwater in southwest coastal Bangladesh is caused by a combination of climatic and anthropogenic factors including sea level rise, frequent cyclones and tidal storms, and shrimp cultivation[19-22]. In the future, climate change and sea-level rise are expected to cause more cyclones, tidal surges and flood in this region that will further affect surface water and groundwater salinity [7]. In many areas in southwest coastal Bangladesh, both shallow and deep aquifers contain naturally brackish water causing acute scarcity of drinking water (**Figure 2A**) [23]. Water salinity in southwest coastal Bangladesh follows a clear seasonal pattern ─ higher in the dry season than the wet season [24]. Salinity of water bodies builds up from October to May, peaking during February to early May. After May, salinity decreases sharply due to rainfall and increased upstream river flow. People collect and use rainwater for drinking and cooking purpose during the monsoon when precipitation is intense, but during the dry season, they generally rely on pond water or saline tube well water [25].

The Geology Department of the University of Dhaka, in collaboration with UNICEF and the Department of Public Health Engineering (DPHE), Government of Bangladesh have piloted 20 small-scale MAR projects in three districts of southwest coastal Bangladesh to evaluate the feasibility of MAR for drinking water supply in rural communities [26]. The shallow brackish aquifer was the target storage zone that is overlain by Holocene clay aquitard of 3 to 15 meter thickness [16]. In the pilot phase, an average storage of approximately 900 m^3 of fresh water per year per site was established, sufficient to deliver 15 L of safe drinking water per day per household, which can fulfill the demand for drinking and cooking for approximately 300 people in 60-70 households during the dry season [27 28]. The second phase of the MAR project started in

For peer review only - http://bmjopen.bmj.com/site/about/guidelines.xhtml

2014 aiming to install 75 MAR systems in three districts of southwest coastal Bangladesh: Satkhira, Khulna and Bagerhat (**Figure 2B**). In March, 2016, 30 MAR sites were handed over to the community for water consumption. The remaining 45 sites are expected to be ready by November 2016 following infiltration of water during monsoon of 2016 (June – October, 2016).

Epidemiological studies have demonstrated that high sodium intake is associated with elevated blood pressure [29-31] and other cardiovascular diseases [32]. A study conducted among the adult population residing in southwest coastal Bangladesh suggest drinking saline water was associated with high blood pressure after adjusting for personal, lifestyle and environmental factors [33]. Among study participants, the mean systolic blood pressure for those consuming water with sodium from the lowest quintile was 119.4 (SD 13.7) and from the highest sodium quintile was 126.7 mm Hg (18·0) [33]. Excess sodium intake from drinking brackish water has been also associated increased gestational hypertension and preeclampsia among pregnant women in southwest coastal Bangladesh[34 35]. Mean systolic and diastolic blood pressure were higher among the pregnant women who drank tube well or pond (saline sources) water compared to those who drank rain water [34 36]. The mean systolic blood pressure of pregnant women from these areas was 102.4 mm Hg among those who drank rainwater, 112.6 mm Hg among pond water users and 119.4 mm Hg among brackish groundwater users [34]. High blood pressure during pregnancy is associated with high maternal mortality, and adverse pregnancy and fetal outcomes [37].

There are 37 million people living in the southwest coastal region and 20 million are currently affected by drinking water salinity [38]. The estimated mean global sodium consumption is 3.95 g per day (range 2.2 to 5.5 g per day) [39], but people in southwest coastal Bangladesh consume up to 16 g of sodium per day through drinking brackish groundwater [40]. While MAR water can potentially reduce exposure to saline water, the health effects of providing access to

For peer review only - http://bmjopen.bmj.com/site/about/guidelines.xhtml

MAR water have not been investigated. Characterizing the health consequences of shifting from current drinking water supply to MAR water can assist decision-makers to assess the value of scaling up MAR.

Objectives

Randomized controlled trials demonstrate that reduction in dietary sodium in adults decreases blood pressure among people both with and without hypertension[41-43]. Meta-analysis of randomized trials also suggest modest reduction in salt intake for four or more weeks causes significant reduction in blood pressure at the population-level [44 45]. We will assess whether access to low-salinity MAR water is associated with cardiovascular health benefits measured by reduction of blood pressure, urinary sodium and protein excretion among a population ≥20 years old and pregnant women in southwest coastal Bangladesh.

For peer review only - http://bmjopen.bmj.com/site/about/guidelines.xhtml

Methods

Study design: We will implement a stepped-wedge cluster randomized controlled trial in 16 MAR communities. The stepped wedge design allows communities to gradually have access to MAR water; however, the point at which their access commences will be randomly assigned [46]. In this way, each MAR site will contribute data for both the intervention and the control time periods. We will have five monthly steps in the stepped wedge trial (**Figure 3**). In the first step, none of the communities will have MAR water available for drinking and we will collect baseline information. During each subsequent month, four randomly selected communities will receive access to MAR water for drinking and cooking. In the last (fifth) monthly step, all the communities will have access to MAR drinking water.

Study settings: The study will be conducted at the community-level in three districts in southwest coastal Bangladesh ─ Khulna, Satkhira and Bagerhat ─ where 75 MAR systems have been installed. Of these, 30 systems are already in use. We will select 16 of the remaining 45 MAR communities for the study based on consultation with Dhaka University and UNICEF using three criteria: communities have not started drinking MAR water, acceptable level of arsenic in MAR water, and electrical conductivity (a measure of salinity) of water below 2000 µS/cm, an indication that the MAR system has successfully reduced salinity in the aquifer.

Participant eligibility criteria: The inclusion criteria will be households whose ≥ 20 year old members and pregnant women willingly agree to exclusively use MAR water during the dry season for drinking and cooking purposes. We will enroll post-adolescent ≥ 20 year population as an individual's response to salt intake (salt-sensitivity) increases with age [47] and adolescence is associated adrenal and nervous system maturation that may contribute to salt sensitivity [48 49]. We will approach households living near the MAR water access point. We will enroll the 28

For peer review only - http://bmjopen.bmj.com/site/about/guidelines.xhtml

closest households surrounding each MAR systems that meet the inclusion criteria and consent to study participation. All household members $\geq$20 year of age will be eligible and enrolled in the study from the selected 28 households. In addition, we will enroll households that include a pregnant woman in each selected MAR catchment community irrespective of household selection, from a list of pregnant women developed by a female promoter.

Intervention: We will randomly select four MAR communities to drink MAR water in each step following the first (baseline) step (**Figure 3**). The Dhaka University team have determined the catchment area under each MAR site based on geographic distance from MAR infrastructure and have developed a list of households who expressed willingness to drink MAR water. We will deploy a local trained promoter at each MAR site, who will visit these households, list members and identify pregnant women in these and other households under catchment areas of each MAR site. Promoters will encourage all household members to drink MAR water exclusively while they are at home, carry a bottle of MAR water while they go out for work and other activities, and to cook with MAR water.

Sample size: The sample size of the stepped wedge trial was calculated based on the primary objective: reduction of systolic blood pressure. Data from southwest coastal Bangladesh demonstrated a 7 mmHg lower mean systolic blood pressure among pregnant women who drank pond water compared to those who drank brackish water, and a 17 mmHg lower mean systolic blood pressure for pregnant women who drank rainwater compared to those who drank brackish water [34]. The mean reduction in systolic blood pressure following long term modest salt reduction is 4.2 mmHg [44]. For calculations we considered a mean systolic blood pressure reduction of at least 3 mmHg among the communities who will have access to MAR water for drinking and cooking purposes compared to those who will use brackish water. The entire

For peer review only - http://bmjopen.bmj.com/site/about/guidelines.xhtml

population under the MAR community catchment area will be considered as one cluster. Each MAR community serves approximately 300 people, constituting 60-70 households in the catchment area [27]. The mean household size in southwest coastal Bangladesh is 4.2, approximately 52% of household members are ≥ 20 years [50 51]. Since we will collect blood pressure for all household members ≥ 20 years, we estimated an average of 2.2 participants in this age group per household. We will select 28 households per cluster (28 *2.2 = 60 people per cluster). A previously published study in Bangladesh reported standard deviation (SD) for systolic blood pressure as 13.51 [52], but we considered standard deviation of systolic blood pressure 20 to capture a greater variation of blood pressure. We calculated the sample size for the stepped wedge trial adopting the formula used by Woertman et al. 2013 and Hemming et al. 2015 [53 54]. Woertman et al. proposed the usual approach of initially calculating the sample size for a simple unadjusted trial (N_U), and then multiplying by the design effect for a stepped wedge trial (DE_{SW}). The sample size for a simple unadjusted trial was calculated as 1,396 participants (effect size= 3, standard deviation of 20 for both groups, 80% power, and 5% Type I error). The DE_{SW} was 3.04 based on a formula provided by Woertman et al 2013. Considering a fixed cluster size of 60, we will need 16 MAR communities, with four communities randomized to access MAR water in each step (**Figure 3**).

Data collection methods: The outcomes of interest will be measured from selected participants of 16 communities irrespective of their access to MAR water during five monthly visits. The interval between two successive blood pressure measurements or two consecutive data collection visits will be at least one month for each participant. After taking informed written consent from household heads and each participant, research staff will administer a survey using a structured questionnaire programmed on handheld computers to collect information on drinking water

For peer review only - http://bmjopen.bmj.com/site/about/guidelines.xhtml

sources, household members' socio-demographic status, participants' medical history and cardiovascular risk factors including smoking history and alcohol intake during the first monthly visit. At this time they will also measure participants' weight, height, waist and hip circumference and blood pressure, and collect urine samples. During subsequent monthly visits, research staff will measure blood pressure, weight and collect urine samples. If a pregnant woman is identified, research staff will measure her blood pressure, and collect urine and blood samples at every monthly visits.

Exposure assessment: During each visit, research staff will inquire about household members' reported primary water sources used for drinking and cooking purposes in last 24 hours, collect reported information on whether participants exclusively used the primary water sources and explore whether any alternative water sources were used. They will also ask about the frequency of collection and cost of primary water sources for drinking and cooking, time required to collect water, amount of collected water and when the last water was collected. Research staff will observe the presence of stored water in households, ask the sources of each container of stored water and will collect water samples that have been stored for drinking and cooking (if any) to measure the salinity level, electrical conductivity, resistivity, total dissolve solutes (TDS) and temperature of water samples using Hanna Salinity™ meter. They will also collect the MAR water from the source MAR outlet to measure the salinity level, electrical conductivity, resistivity, total dissolve solutes (TDS) and temperature of water. Research staff will collect 24 hour urine from each participant to measure the urinary sodium excretion as a proxy for sodium intake[55 56].

Outcomes assessment: Systolic blood pressure of the ≥ 20 year old population and pregnant women is the primary outcome of this study. Secondary outcomes will be diastolic blood

For peer review only - http://bmjopen.bmj.com/site/about/guidelines.xhtml

pressure, mean arterial pressure, and pulse pressure (**Table 1**). The different components of blood pressure are independent cardiovascular risk factors associated with sodium intake [57-61], and indicate future risk for different cardiovascular diseases [62]. High systolic blood pressure puts more stress on the vascular wall and is strongly associated with risk for future intracerebral haemorrhage, subarachnoid haemorrhage, angina, myocardial infarction and peripheral vascular diseases[62]. Raised diastolic blood pressure is associated with aortic and thoracic aneurysm [62]. Some effects of high sodium intake are independent of high systolic and diastolic blood pressure such as arterial stiffness and left ventricular mass ─ both of these are independent predictor of future cardiovascular diseases. High pulse pressure (difference between systolic and diastolic blood pressure) is associated with increased arterial stiffness [58 60], and high mean arterial pressure (diastolic blood pressure plus one-third of systolic blood pressure) and high pulse pressure is independently associated with increased left ventricular mass [58 60].

We will also measure the creatinine adjusted protein excretion of all participants and urinary marinobufagenin (MBG) and serum uric acid among pregnant women as tertiary outcomes (**Table 1**). Proteinuria is a biomarker for future risk of cardiovascular diseases [63-66], and is associated with the pathogenesis of cardiovascular diseases, including hypertension [67 68], chronic kidney disease [69], myocardial ischemia [70], carotid artery thickness [71 72], left ventricular hypertrophy [73 74], hyperlipidemia [75], atherosclerosis [76], and coronary artery calcification [64 72 77 78]. Reduced salt intake for four weeks has been associated with decreased proteinuria in blinded randomized controlled trial [79]. Preeclampsia is associated with high maternal mortality and adverse pregnancy and fetal outcomes [37] and is characterized by high blood pressure and proteinuria after 20 weeks of pregnancy [80]. Salt intake causes increased release of

For peer review only - http://bmjopen.bmj.com/site/about/guidelines.xhtml

marinobufagenin and uric acid, which has been associated with the pathogenesis of preeclampsia[81-83].

Blood pressure measurement: Blood pressure will be measured for all ≥20 year old participants and pregnant women during each step. Research staff will use a Omron® HEM–907 blood pressure device, which is comparable to the gold standard mercury sphygmomanometers in terms of measurement and meets the Association for the Advancement of Medical Instrumentation's (AAMI) criteria [84]. Blood pressure will be measured following the guidelines described by Pickering et. al. 2005 and Giorgini et. al 2014 [85 86]. Caffeine (tea, coffee, carbonated beverages), eating, heavy physical activities and smoking will be proscribed for 30 minutes prior to measuring blood pressure. Participants will rest for 5 minutes sitting on a chair keeping their arm supported. An appropriate sized cuff and calibrated instrument will be used for different age groups and the blood pressure instrument will be positioned at heart level. Blood pressure will be measured three times; first left arm, then right arm, then again left arm. Both systolic and diastolic blood pressure will be recorded from all measurements. The arithmetic mean of three systolic blood pressure measurements will be used as the primary outcome. However, if a systolic blood pressure measurement differs by 10% from the other measurements, that measurement will be excluded when calculating the arithmetic mean systolic blood pressure.

Biomarker measurements: Field research staff will instruct the participants' to collect 24 hours urine sample during each household visit. The volume of the urine samples will be noted at household level, and a sample of 25 ml urine will be collected and transported to the field laboratory at 2-8° C within 6 hours of collection for processing, analysis and storage. Aliquots of each participant's urine sample will be made for biochemical and electrolyte measurements. Urinary creatinine concentration will be measured by a colorimetric method (Jaffe reaction)

For peer review only - http://bmjopen.bmj.com/site/about/guidelines.xhtml

using a semi-auto biochemistry analyzer (Evolution 3000, BSI, Italy). Urinary total protein will be estimated using a light sensitive colored reagent (Randox.UK). We will use the direct Ion Selective Electrode (ISE) method for urinary sodium measurements using a semi-auto electrolyte analyzer (Biolyte2000, Bio-care Corporation,Taiwan). We will measure the uric acid concentration in blood by an enzymatic colorimetric method (Evolution 3000, BSI, Italy). We will perform routine Quality Control for all tests using standard quality control reagents (Bio-Rad Laboratories, USA) and 5% of samples will be cross checked at the International Centre for Diarrhoeal Disease Research, Bangladesh (icddr,b) central laboratory, in Dhaka. We will use a chemifluorescent competitive enzyme-linked immunosorbent assay (ELISA) that quantifies marinobufagenin (MBG) levels in urine.

Statistical methods: We will conduct an intention-to-treat analysis. Since we will have longitudinal data on individual participants, generalized estimating equations (GEE) with an appropriate autoregressive correlation structure, to account for repeated measures within individual study participants will be used as primary analysis. The systolic blood pressure will be regressed on an indicator variable for access to MAR water, and dummy variables for the study sites and time periods. The interpretation of GEE model output will be the population average association of MAR water exposure with blood pressure, conditional on study site. In secondary analyses, we will adjust for age, sex, weight and height, personal, dietary and environmental factors **(Table 2).** We will use weighted GEE or multiple imputations methods to account for missing observations. In secondary analyses, we will consider the association of drinking MAR water with diastolic blood pressure, pulse pressure, mean arterial pressure, urinary sodium and protein concentrations. For the primary analysis of pregnant women for systolic blood pressure

For peer review only - http://bmjopen.bmj.com/site/about/guidelines.xhtml

outcome, we will a run random effect model considering MAR (dummy variable) and gestational months as predictor variables.

Ethics

Informed written consent will be taken from all participants and household heads. This study protocol has been reviewed and approved by the icddr,b Ethical Review Committee. If research staff members identify hypertensive patients or cases of hypertension during pregnancy, they will refer patients to the local government health facilities for further management. In addition, research staff will train pregnant women and family members to recognize the danger signs during pregnancy, and will also instruct them where to seek medical care if such danger signs appear. During monthly household visits research staff will encourage pregnant women to attend prenatal visits.

Dissemination

Study findings will be shared with the Department of Public Health Engineering (DPHE), Department of Environment of the government of Bangladesh and other partner NGOs working in southwest coastal Bangladesh for safe access to drinking water. We will discuss the scope and limitations of the MAR system to address the demand of safe drinking water based on findings from this study. We will be submitting abstracts to international conferences for dissemination to the international audience working on safe water. We will develop manuscripts and submit to peer-reviewed journals to publish our research.

For peer review only - http://bmjopen.bmj.com/site/about/guidelines.xhtml

For peer review only

Discussion

This will be the first study to assess the health impact of an environmental intervention to reduce groundwater salinity in southwest coastal Bangladesh. Our study has several strengths. The stepped-wedge trial ensures that we will have counterfactual data as well as gradual access to MAR water in all communities. In a stepped-wedge design, treatment effect of an intervention can be estimated from between- and within-cluster comparisons as participants will act as their own control, compared to only between-cluster comparison in a parallel cluster randomized design [87]. We will use the same instruments for data collection and outcome measurements for all steps for exposed and unexposed communities, which will mitigate bias in data collection. Measurement of water source salinity and urinary sodium concentration will explain the biologically plausible effect of drinking MAR water on blood pressure. The primary, secondary and tertiary outcomes are objective and would reduce the risk of reporting bias. Collection of detailed exposure and co-variate data will help in determining a valid association between drinking MAR water and health benefits. We will have several outcome variables and biomarkers that will ensure a comprehensive health benefit evaluation of access to MAR water.

The study has several limitations. We will be unable to control salinity level of drinking water across the 16 MAR communities due to different hydro-geological conditions and volume of infiltrated water across the aquifers. Although the initial electrical conductivity of MAR water will be < 2000 μS/cm, electrical conductivity is likely to vary across different MAR communities with the intensification of dry season. Therefore, MAR water salinity will differ across communities at a single point of time, and also for the same community at different points of time. This will result in different versions of exposures (i.e. access to MAR water with different salinity-levels) that may affect different responses for some participants depending on the

For peer review only - http://bmjopen.bmj.com/site/about/guidelines.xhtml

version of exposure they will receive [88 89]. To account this, we will specify the versions of exposure by measuring the salinity level of MAR water available in participants' households and interpret the response as unit change of salinity at different MAR water salinity-levels. As compliance of drinking MAR water may be different across communities despite active encouragement by promoters, one problem of the intention to treat analysis is that if the proportions of participants who will always drink MAR water is low compared to those who will not, the potentially greater effect of MAR intervention on blood pressure of fully participating individuals may be washed out by the smaller effect of those who will not comply. To account for this, as a secondary analysis, we will conduct "instrumental variable" (IV) analyses by jointly running two regression models: a regression model for predicting urinary sodium excretion by drinking water salinity, and a regression model for blood pressure prediction given the urinary sodium excretion. The assumption of instrumental variable analyses is fulfilled in our context as the exogenous variable (instrumental variable) can only affect outcome variable (blood pressure) through influencing the endogenous variable (urinary sodium excretion) [90]. If MAR water has lower-salinity than other water sources and if participants always drink MAR water, their urinary sodium excretion will be low compared to those who will not fully participate.

Acknowledgements

The study has been funded by Wellcome Trust, UK, Our Planet, Our Health Award. Wellcome Trust reviewed and approved the design as the condition of providing funding but will not have a role in data collection, data analysis, data interpretation, or writing of the report. icddr,b acknowledges with gratitude the commitment of Wellcome Trust, UK to its research

For peer review only - http://bmjopen.bmj.com/site/about/guidelines.xhtml

efforts. icddr,b is also grateful to the Governments of Bangladesh, Canada, Sweden and the UK for providing core/unrestricted support. Dr. Gribble is supported by funding from the National Institute for Environmental Health Sciences to the Emory Health and Research Exposome Center (P30 ES019776). The authors would like to thank Dr. Karla Hemming, Institute of Applied Health Research, University of Birmingham for her input in and sample size calculation. We are grateful to the Dhaka University/UNICEF field team for their assistance in providing preliminary data about the readiness of MAR sites.

Contributors: SPL and AMN developed the study concept. AMN, SPL, LU KMA, and SD have developed the study design. MR, MOG, TFC and HHC reviewed the epidemiological study design. KMA, MS, WB, SS and MNU provided input in environmental and biological sample collection and analysis. HHC and MOG provided input in statistical analysis. AMN drafted and all author reviewed the manuscript.

Competing interest: None

Funding: Wellcome Trust (GR 01336).

Data sharing statement: The anonymous dataset without personal identifiers will be publicly available following publication of the primary trial results.

For peer review only

References-

1. Van Weert F, van der Gun J, Reckman J. Global overview of saline groundwater occurrence and genesis. International Groundwater Resources Assessment Centre 2009.
2. Vengosh A, Rosenthal E. Saline groundwater in Israel: its bearing on the water crisis in the country. Journal of Hydrology 1994;**156**(1):389-430.
3. Post V. Fresh and saline groundwater interaction in coastal aquifers: Is our technology ready for the problems ahead? Hydrogeology Journal 2005;**13**(1):120-23.
4. Holman IP. Climate change impacts on groundwater recharge-uncertainty, shortcomings, and the way forward? Hydrogeology journal 2006;**14**(5):637-47.
5. Ferguson G, Gleeson T. Vulnerability of coastal aquifers to groundwater use and climate change. Nature Climate Change 2012;**2**(5):342-45.
6. Mall R, Gupta A, Singh R, et al. Water resources and climate change: an Indian perspective. Current Science 2006;**90**(12):1610-26.
7. Taylor RG, Scanlon B, Döll P, et al. Ground water and climate change. Nature Climate Change 2013;**3**(4):322-29.
8. Changming L, Jingjie Y, Kendy E. Groundwater exploitation and its impact on the environment in the North China Plain. Water International 2001;**26**(2):265-72.
9. Counts HB, Donsky E. *Salt-water Encroachment Geology and Ground-water Resources of Savannah Area Georgia and South Carolina*: US Government Printing Office, 1963.
10. Barlow PM. *Ground Water in Fresh Water-salt Water Environments of the Atlantic*: Geological Survey (USGS), 2003.
11. McGranahan G, Balk D, Anderson B. The rising tide: assessing the risks of climate change and human settlements in low elevation coastal zones. Environment and urbanization 2007;**19**(1):17-37.
12. Villholth KG. Integrated groundwater use and management in vulnerable coastal zones of Asia-Pacific. Groundwater in the Coastal Zones of Asia-Pacific: Springer, 2013:317-42.
13. Morris BL, Lawrence AR, Chilton P, et al. Groundwater and its susceptibility to degradation: a global assessment of the problem and options for management. 2003.
14. Dillon P. Future management of aquifer recharge. Hydrogeology journal 2005;**13**(1):313-16.
15. Bouwer H. Artificial recharge of groundwater: systems, design, and management. Hydraulic design handbook McGraw-Hill, New York 1999:24.1-24.44.
16. Ahmed K, Sultana S, Groen K, et al. DEVELOPMENT OF OPTIMAL DESIGN OF AQUIFER STORAGE AND RECOVERY FOR PROVIDING SAFE WATER TO VULNERABLE COMMUNITIES OF COASTAL BANGLADESH. International conference on Climate Change in relation to Water and Environment (I3CWE-2015). Department of Civil Engineering, DUET - Gazipur, Bangladesh, 2015:1-8.
17. Dillon P, Toze S, Page D, et al. Managed aquifer recharge: rediscovering nature as a leading edge technology. Water Science and Technology 2010;**62**(10):2338.
18. Groundwater and Climate Change: Challenges and Possibilities 2008 [Available from: http://www.geus.dk/program-areas/water/denmark/rapporter/groundwater_and_%20climate_change_071108.pdf.
19. Allison MA, Khan SR, Goodbred SL, et al. Stratigraphic evolution of the late Holocene Ganges-Brahmaputra lower delta plain. Sedimentary Geology 2003;**155**(3-4):317-42.
20. Mirza MMQ. Diversion of the Ganges water at Farakka and its effects on salinity in Bangladesh. Environmental management 1998;**22**(5):711-22.
21. Karim MR. Brackish-water shrimp cultivation threatens permanent damage to coastal agriculture in Bangladesh. Environment and livelihoods in tropical coastal zones: managing agriculture-fishery-aquaculture conflicts 2006;**2**:61-71.

For peer review only - http://bmjopen.bmj.com/site/about/guidelines.xhtml

22. Mahmuduzzaman M, Ahmed ZU, Nuruzzaman A, et al. Causes of Salinity Intrusion in Coastal Belt of Bangladesh. International Journal of Plant Research 2014;**4**(4A):8-13.
23. Bahar MM, Reza MS. Hydrochemical characteristics and quality assessment of shallow groundwater in a coastal area of Southwest Bangladesh. Environmental Earth Sciences 2010;**61**(5):1065-73.
24. Changes of the seasonal salinity distribution at the Sundarbans coast due to impact of climate change. 4th International Conference on Water & Flood Management (ICWFM); 2013.
25. Islam MA, Sakakibara H, Karim MR, et al. Potable water scarcity: options and issues in the coastal areas of Bangladesh. Journal of water and health 2013;**11**(3):532-42.
26. Acacia Water DU. Action reserach: Creating fresh water bubbles in Brackish aquifer 2011 [Available from: http://www.google.com.bd/url?sa=t&rct=j&q=creating+Fresh+water+bubbles+in+Brackish+aquifers&source=web&cd=1&cad=rja&ved=0CCcQFjAA&url=http%3A%2F%2Facaciawater.com%2Fsite%2Fmedia%2Fprojecten%2FFlyer_MAR_BD.pdf&ei=Q6gHUZrHDKe0iQfdjoCADg&usg=AFQjCNHc5fhWh5Nk2v-jzz0s6PTsztFAqg&bvm=bv.41524429,d.aGc.
27. UNICEF. Underground fresh water storage A practical solution to increase water security in saline deltas 2014 [Available from: http://www.spaarwater.com/content/27227/download/clnt/57128_Underground_fresh_water_storage_-_a_practical_solution_to_increase_water_security_in_saline_deltas.pdf.
28. Sultana S, Ahmed K, Mahtab-Ul-Alam S, et al. Low-Cost Aquifer Storage and Recovery: Implications for Improving Drinking Water Access for Rural Communities in Coastal Bangladesh. Journal of Hydrologic Engineering 2014.
29. Alderman MH. Salt, blood pressure, and human health. Hypertension 2000;**36**(5):890-3.
30. Cutler JA, Follmann D, Allender PS. Randomized trials of sodium reduction: an overview. Am J Clin Nutr 1997;**65**(2 Suppl):643S-51S.
31. Midgley JP, Matthew AG, Greenwood CM, et al. Effect of reduced dietary sodium on blood pressure: a meta-analysis of randomized controlled trials. JAMA 1996;**275**(20):1590-7.
32. Strazzullo P, D'Elia L, Kandala N-B, et al. Salt intake, stroke, and cardiovascular disease: meta-analysis of prospective studies. Bmj 2009;**339**.
33. Scheelbeek PF, Chowdhury MA, Haines A, et al. High concentrations of sodium in drinking water and raised blood pressure in coastal deltas affected by episodic seawater inundations. The Lancet Global Health 2016;**4**:S18.
34. Khan AE, Scheelbeek PFD, Shilpi AB, et al. Salinity in Drinking Water and the Risk of (Pre) Eclampsia and Gestational Hypertension in Coastal Bangladesh: A Case-Control Study. 2014.
35. Khan AE, Ireson A, Kovats S, et al. Drinking Water Salinity and Maternal Health in Coastal Bangladesh: Implications of Climate Change. Environ Health Perspect 2011.
36. Scheelbeek PF, Khan AE, Mojumder S, et al. Drinking Water Sodium and Elevated Blood Pressure of Healthy Pregnant Women in Salinity-Affected Coastal Areas. Hypertension 2016;**68**(2):464-70.
37. Brown MA, Lindheimer MD, de Swiet M, et al. The classification and diagnosis of the hypertensive disorders of pregnancy: statement from the International Society for the Study of Hypertension in Pregnancy (ISSHP). Hypertension in pregnancy 2001;**20**(1):ix-xiv.
38. Dasgupta S, Kamal FA, Khan ZH, et al. River salinity and climate change: evidence from coastal Bangladesh. World Bank Policy Research Working Paper 2014(6817).
39. Mozaffarian D, Fahimi S, Singh GM, et al. Global sodium consumption and death from cardiovascular causes. New England Journal of Medicine 2014;**371**(7):624-34.
40. Khan AE, Ireson A, Kovats S, et al. Drinking Water Salinity and Maternal Health in Coastal Bangladesh: Implications of Climate Change. Environ Health Perspect.
41. He FJ, MacGregor GA. Effect of longer-term modest salt reduction on blood pressure. Cochrane Database Syst Rev 2004;**3**.

For peer review only - http://bmjopen.bmj.com/site/about/guidelines.xhtml

42. Organization WH. *Guideline: Sodium intake for adults and children*: World Health Organization, 2012.
43. Graudal NA, Hubeck-Graudal T, Jurgens G. Effects of low sodium diet versus high sodium diet on blood pressure, renin, aldosterone, catecholamines, cholesterol, and triglyceride. Cochrane Database Syst Rev 2011;**11**.
44. He FJ, Li J, MacGregor GA. Effect of longer term modest salt reduction on blood pressure: Cochrane systematic review and meta-analysis of randomised trials. 2013.
45. He F, MacGregor G. Effect of modest salt reduction on blood pressure: a meta-analysis of randomized trials. Implications for public health. Journal of human hypertension 2002;**16**(11):761-70.
46. De Allegri M, Pokhrel S, Becher H, et al. Step-wedge cluster-randomised community-based trials: an application to the study of the impact of community health insurance. Health Res Policy Syst 2008;**6**:10.
47. Luft FC, Miller JZ, Grim CE, et al. Salt sensitivity and resistance of blood pressure. Age and race as factors in physiological responses. Hypertension 1991;**17**(1 Suppl):I102.
48. Orchard T, Rodgers M, Hedley A, et al. Changes in blood lipids and blood pressure during adolescence. Br Med J 1980;**280**(6231):1563-67.
49. Fujita T. Mechanism of salt-sensitive hypertension: focus on adrenal and sympathetic nervous systems. Journal of the American Society of Nephrology 2014;**25**(6):1148-55.
50. BBS. POPULATION AND HOUSING CENSUS 2011 [Available from: http://203.112.218.66/WebTestApplication/userfiles/Image/BBS/Socio_Economic.pdf.
51. NIPORT. Bangladesh Demographic and Health Survey: Preliminary Report. Dhaka, 2011.
52. Khan SH, Talukder SM, Zaman MM. Non-communicable Disease Prevention Intervention in Production Sector of Bangladesh: Findings and Lessons Learnt. 13th ASCON. icddr,b, Dhaka, 2011.
53. Woertman W, de Hoop E, Moerbeek M, et al. Stepped wedge designs could reduce the required sample size in cluster randomized trials. Journal of clinical epidemiology 2013;**66**(7):752-58.
54. Hemming K, Lilford R, Girling AJ. Stepped-wedge cluster randomised controlled trials: a generic framework including parallel and multiple-level designs. Statistics in medicine 2015;**34**(2):181-96.
55. McLean RM. Measuring population sodium intake: a review of methods. Nutrients 2014;**6**(11):4651-62.
56. Brown IJ, Dyer AR, Chan Q, et al. Estimating 24-hour urinary sodium excretion from casual urinary sodium concentrations in western populations the INTERSALT study. American journal of epidemiology 2013;**177**(11):1180-92.
57. Bednarski A, Stolarz-Skrzypek K, Kielbasa G, et al. Pulse pressure in relation to 24-hour urinary sodium excretion in a sample of high-salt intake population. Artery Research 2015;**12**:5.
58. Staessen J, Bulpitt CJ, Thijs L, et al. Pulse Rate and Sodium Intake Interact to Determine Blood Pressure A Population Study. American journal of hypertension 1991;**4**(2 Pt 1):107-12.
59. Weir MR, Dengel DR, Behrens MT, et al. Salt-induced increases in systolic blood pressure affect renal hemodynamics and proteinuria. Hypertension 1995;**25**(6):1339-44.
60. Du Cailar G, Mimran A. Non-pressure-related effects of dietary sodium. Current hypertension reports 2009;**11**(1):12-17.
61. Cheung B, Ho S, Cheung A, et al. Diastolic blood pressure is related to urinary sodium excretion in hypertensive Chinese patients. QJM 2000;**93**(3):163-68.
62. Rapsomaniki E, Timmis A, George J, et al. Blood pressure and incidence of twelve cardiovascular diseases: lifetime risks, healthy life-years lost, and age-specific associations in 1· 25 million people. The Lancet 2014;**383**(9932):1899-911.

For peer review only - http://bmjopen.bmj.com/site/about/guidelines.xhtml

63. Kramer H, Jacobs DR, Bild D, et al. Urine albumin excretion and subclinical cardiovascular disease. Hypertension 2005;**46**(1):38-43.
64. Weir MR. Microalbuminuria and cardiovascular disease. Clinical Journal of the American Society of Nephrology 2007;**2**(3):581-90.
65. Luft FC, Agrawal B. Microalbuminuria as a predictive factor for cardiovascular events. Journal of cardiovascular pharmacology 1999;**33**:S11-S15.
66. Bigazzi R, Bianchi S, Baldari D, et al. Microalbuminuria in salt-sensitive patients. A marker for renal and cardiovascular risk factors. Hypertension 1994;**23**(2):195-99.
67. Bianchi S, Bigazzi R, Campese VM. Microalbuminuria in essential hypertension: significance, pathophysiology, and therapeutic implications. American journal of kidney diseases 1999;**34**(6):973-95.
68. Agrawal B, Berger A, Wolf K, et al. Microalbuminuria screening by reagent strip predicts cardiovascular risk in hypertension. Journal of hypertension 1996;**14**(2):223-28.
69. Sarafidis PA, Bakris GL. Microalbuminuria and chronic kidney disease as risk factors for cardiovascular disease. Nephrology Dialysis Transplantation 2006;**21**(9):2366-74.
70. Diercks G, Van Boven A, Hillege H, et al. Microalbuminuria is independently associated with ischaemic electrocardiographic abnormalities in a large non-diabetic population. The PREVEND (Prevention of REnal and Vascular ENdstage Disease) study. European heart journal 2000;**21**(23):1922-27.
71. Bigazzi R, Bianchi S, Nenci R, et al. Increased thickness of the carotid artery in patients with essential hypertension and microalbuminuria. Journal of human hypertension 1995;**9**(10):827-33.
72. Mykkänen L, Zaccaro DJ, O'Leary DH, et al. Microalbuminuria and carotid artery intima-media thickness in nondiabetic and NIDDM subjects The Insulin Resistance Atherosclerosis Study (IRAS). Stroke 1997;**28**(9):1710-16.
73. Pedrinelli R, Di Bello V, Catapano G, et al. Microalbuminuria is a marker of left ventricular hypertrophy but not hyperinsulinemia in nondiabetic atherosclerotic patients. Arteriosclerosis, Thrombosis, and Vascular Biology 1993;**13**(6):900-06.
74. Assadi F. Relation of left ventricular hypertrophy to microalbuminuria and C-reactive protein in children and adolescents with essential hypertension. Pediatric cardiology 2008;**29**(3):580-84.
75. Campese VM, Bianchi S, Bigazzi R. Association between hyperlipidemia and microalbuminuria in essential hypertension. Kidney International 1999;**56**:S10-S13.
76. Jensen JS, Borch-Johnsen K, Jensen G, et al. Atherosclerotic risk factors are increased in clinically healthy subjects with microalbuminuria. Atherosclerosis 1995;**112**(2):245-52.
77. Klausen K, Borch-Johnsen K, Feldt-Rasmussen B, et al. Very low levels of microalbuminuria are associated with increased risk of coronary heart disease and death independently of renal function, hypertension, and diabetes. Circulation 2004;**110**(1):32-35.
78. Currie G, Delles C. Proteinuria and its relation to cardiovascular disease. International journal of nephrology and renovascular disease 2013;**7**:13-24.
79. Swift PA, Markandu ND, Sagnella GA, et al. Modest salt reduction reduces blood pressure and urine protein excretion in black hypertensives A randomized control trial. Hypertension 2005;**46**(2):308-12.
80. Leeman L, Fontaine P. Hypertensive disorders of pregnancy. American family physician 2008;**78**(1):93-100.
81. Uddin MN, Horvat D, Glaser SS, et al. Examination of the cellular mechanisms by which marinobufagenin inhibits cytotrophoblast function. Journal of Biological Chemistry 2008;**283**(26):17946-53.

For peer review only - http://bmjopen.bmj.com/site/about/guidelines.xhtml

82. Uddin MN, Allen SR, Jones RO, et al. Pathogenesis of pre-eclampsia: marinobufagenin and angiogenic imbalance as biomarkers of the syndrome. Translational Research 2012;**160**(2):99-113.
83. Forman JP, Scheven L, de Jong PE, et al. Association between sodium intake and change in uric acid, urine albumin excretion, and the risk of developing hypertension. Circulation 2012:CIRCULATIONAHA. 112.096115.
84. Ostchega Y, Zhang G, Sorlie P, et al. Blood Pressure Randomized Methodology Study Comparing Automatic Oscillometric and Mercury Sphygmomanometer Devices: National Health and Nutrition Examination Survey, 2009–2010. National Health Statistics Reports, 2012.
85. Pickering T, Hall J, Appel L, et al. Blood pressure measurement in humans: A statement for professionals from the subcommittee of professional and public education of the American Heart Association Council on High Blood Pressure Research. J Am Heart Assoc 2005;**45**:142-61.
86. Giorgini P, Weder AB, Jackson EA, et al. A review of blood pressure measurement protocols among hypertension trials: implications for "evidence-based" clinical practice. Journal of the American Society of Hypertension 2014;**8**(9):670-76.
87. Hussey MA, Hughes JP. Design and analysis of stepped wedge cluster randomized trials. Contemporary clinical trials 2007;**28**(2):182-91.
88. Schwartz S, Gatto NM, Campbell UB. Extending the sufficient component cause model to describe the Stable Unit Treatment Value Assumption (SUTVA). Epidemiologic Perspectives & Innovations 2012;**9**(1):3.
89. VanderWeele TJ. Concerning the consistency assumption in causal inference. Epidemiology 2009;**20**(6):880-83.
90. Arellano M, Bover O. Another look at the instrumental variable estimation of error-components models. Journal of econometrics 1995;**68**(1):29-51.

For peer review only

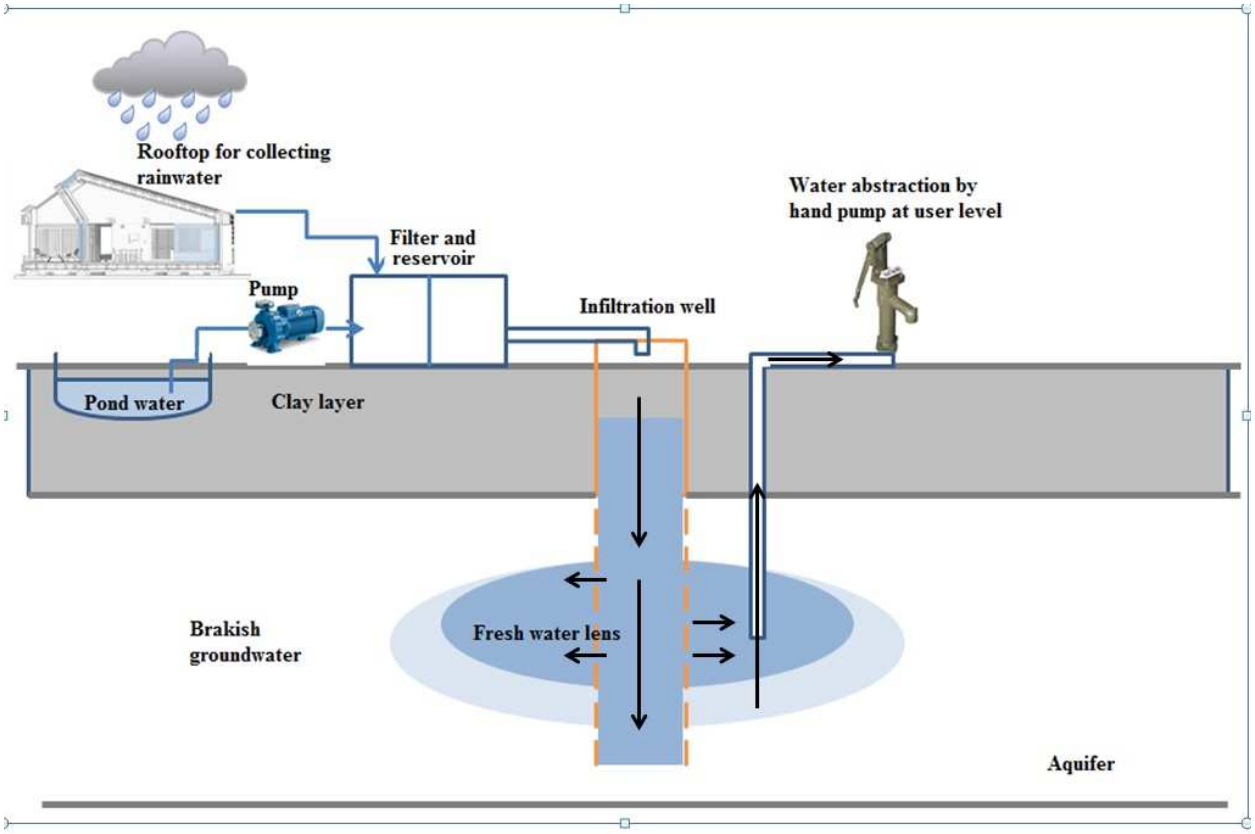

Figure 1: Schematic diagram of the southwest coastal Bangladesh MAR systems

For peer review only - http://bmjopen.bmj.com/site/about/guidelines.xhtml

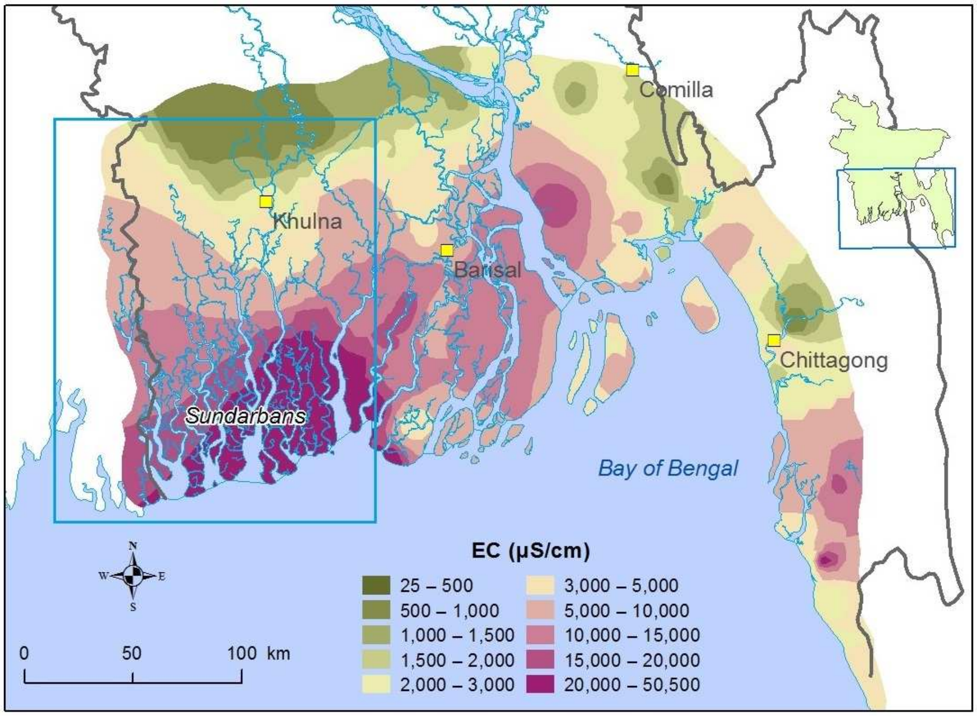

A)

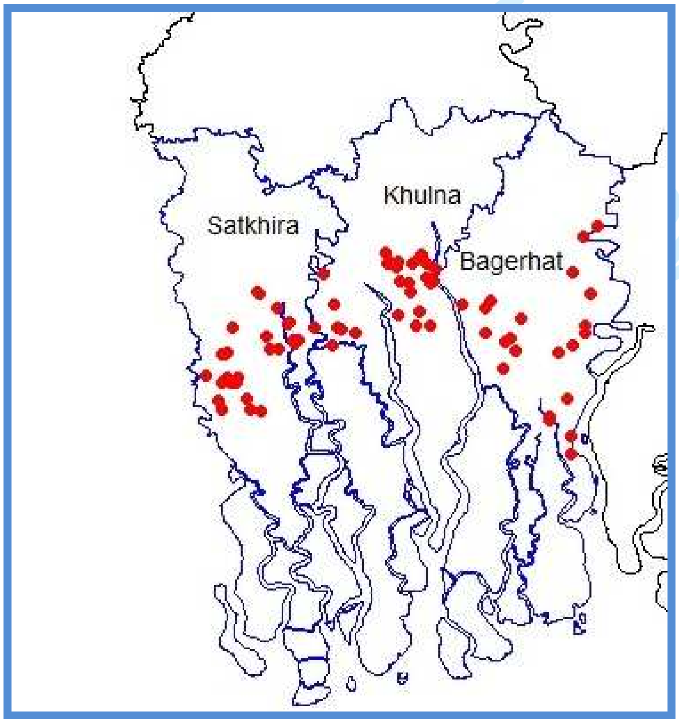

B)

Figure 2: A) Groundwater salinity (measured as EC: electrical conductivity) distribution in coastal Bangladesh and B) location of the 75 MAR sites in southwest coastal region (red dots).

For peer review only - http://bmjopen.bmj.com/site/about/guidelines.xhtml

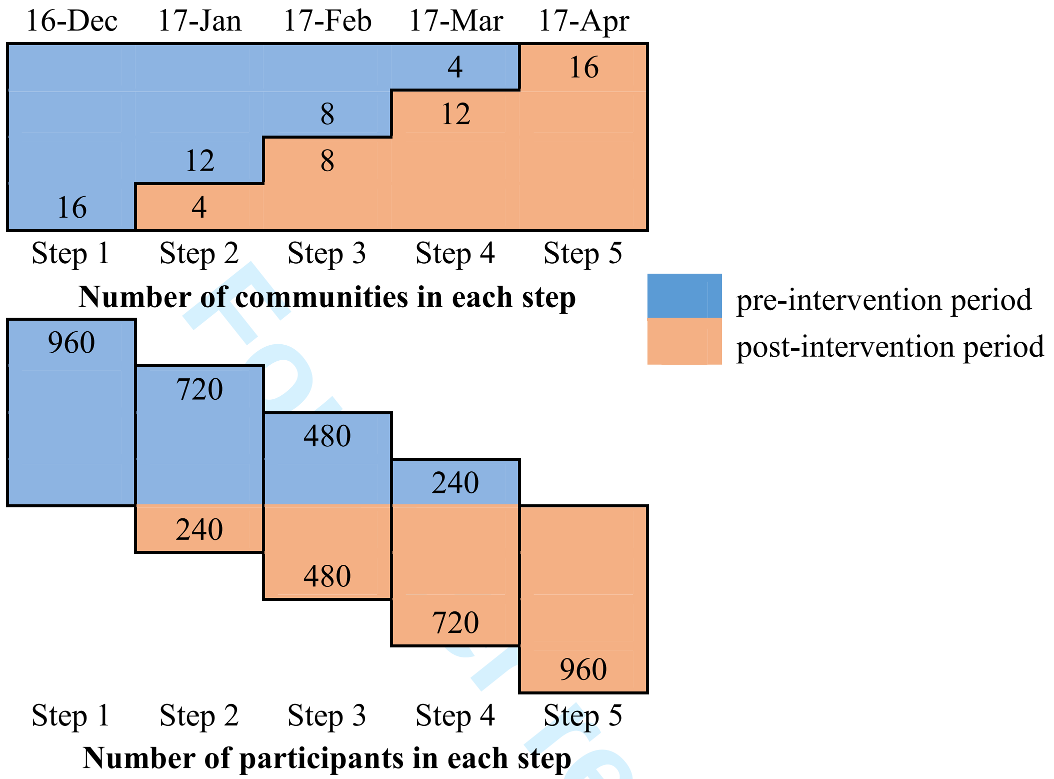

Figure 2: Timeline of blood pressure measurement with number of MAR communities and participants in pre-intervention (blue color) and post-intervention (grey color) time period in different steps

For peer review only - http://bmjopen.bmj.com/site/about/guidelines.xhtml

Table 1: Study outcomes including normal ranges of blood pressure and biomarkers

Outcomes	Study population		Measures	Normal range/calculation
	≥ 20 years old	Pregnant women		
Primary outcome: Systolic blood pressure	Yes	Yes	Systolic blood pressure	90 -140 mmHg
Secondary outcomes: Diastolic blood pressure	Yes	Yes	Diastolic blood pressure	60 -90 mmHg
Mean arterial pressure	Yes	Yes	Mean arterial pressure	Diastolic blood pressure + 1/3 systolic blood pressure
Pulse pressure	Yes	Yes	Pulse pressure	Systolic blood pressure – diastolic blood pressure
Tertiary outcomes: Urinary sodium excretion	Yes	Yes	Urinary sodium-creatinine ratio	40-220 mEq/L/24 hours
Proteinuria	Yes	Yes	Urinary protein-creatinine ratio	< 0.11 mg/mg
Serum uric acid	No	Yes	Serum uric acid concentration	2 – 6.3 mg/dl* (normal pregnancy)
Urinary MBG excretion	No	Yes	Urinary MBG-creatinine ratio	< 1000 pg/mg* (normal pregnancy)

* Concentration varies across gestational age

For peer review only - http://bmjopen.bmj.com/site/about/guidelines.xhtml

Table 2: Key variables to be used for assessing the health impact of access to MAR water

**Outcomes**	**Exposures**	**Confounders**	**Co-variates**	**Moderators**
Systolic blood pressure	Drinking water sources	Sex	Age	Presence of another water sources
Diastolic blood pressure	Drinking water salinity	Socio-economic status	Weight	Drinking/cooking from another water sources
Pulse pressure	Cooking water sources	Education	Height	
Mean arterial pressure	Cooking water salinity	Ambient average daily temperature	Waist circumference	
Urinary total protein		Ambient maximum daily temperature	Mid upper arm circumference	
		Humidity	Adding table salt	
		Rainfall	Adding salt for cooking	
		Sunshine	Exercise level	
		Day light hours	Smoking status	
			Use of betel nuts	
			Stress	
			Diet	
			Urinary potassium	

For peer review only

For peer review only - http://bmjopen.bmj.com/site/about/guidelines.xhtml

Stepped-wedge cluster-randomized controlled trial to assess the cardiovascular health effects of a managed aquifer recharge initiative to reduce drinking water salinity in southwest coastal Bangladesh: study design and rationale

Journal:	*BMJ Open*
Manuscript ID	bmjopen-2016-015205.R1
Article Type:	Protocol
Date Submitted by the Author:	31-May-2017

Complete List of Authors:	Naser, Abu; Emory University, Department of Environmental Health Sciences, Rollins School of Public Health<br>Unicomb, Leanne; International Centre for Diarrhoeal Disease Research, Centre for Communicable Diseases<br>Doza, Solaiman; International Centre for Diarrhoeal Disease Research, Centre for Communicable Diseases<br>Ahmed, Kazi; Dhaka University, Department of Geology<br>Rahman, Mahbubur ; International Centre for Diarrhoeal Disease Research, Centre for Communicable Diseases<br>Uddin, Mohammad; International Centre for Diarrhoeal Disease Research, Centre for Communicable Diseases<br>Quraishi, Shamshad; Atomic Energy Centre, Analytical Chemistry Laboratory<br>Selim, Shahjada; Bangabandhu Sheikh Mujib Medical University<br>Shamsudduha, Mohammad ; University College London, Institute for Risk and Disaster Reduction<br>Burgess, William; University College London, Department of Earth Sciences<br>Chang, Howard; Emory University, Department of Biostatistics and Bioinformatics, Rollins School of Public Health, Emory University<br>Gribble, Matthew; Emory University, Department of Environmental Health Sciences, Rollins School of Public Health<br>Clasen, Thomas; Emory University, Department of Environmental Health Sciences, Rollins School of Public Health<br>Luby, Stephen; International Centre for Diarrhoeal Disease Research, Centre for Communicable Diseases; Stanford University, Woods Institute for the Environment

**Primary Subject Heading**:	Public health
Secondary Subject Heading:	Epidemiology, Global health, Occupational and environmental medicine, Cardiovascular medicine
Keywords:	stepped-wedge trial, Managed aquifer recharge, Blood pressure, Sodium intake, Drinking saline water, Coastal aquifer

For peer review only

Stepped-wedge cluster-randomized controlled trial to assess the cardiovascular health effects of a managed aquifer recharge initiative to reduce drinking water salinity in southwest coastal Bangladesh: study design and rationale

Abu Mohd Naser[1*], Leanne Unicomb[2], Solaiman Doza[2], Kazi Matin Ahmed[3], Mahbubur Rahman[2], Mohammad Nasir Uddin[2], Shamshad B. Quraishi[4], Shahjada Selim[5], Mohammad Shamsudduha[6], William Burgess[7], Howard H. Chang[8], Matthew O. Gribble[1], Thomas F. Clasen[1], Stephen P. Luby[9]

[1] Department of Environmental Health Sciences, Rollins School of Public Health, Emory University, Atlanta, USA

[2]International Centre for Diarrhoeal Disease Research, Bangladesh (icddr,b), GPO Box 128, Dhaka 1000, Bangladesh

[3] Department of Geology, University of Dhaka, Dhaka, Bangladesh

[4]Analytical Chemistry Laboratory, Atomic Energy Centre, Dhaka, Bangladesh

[5]Bangabandhu Sheikh Mujib Medical University, Dhaka, Bangladesh

[6] Institute for Risk and Disaster Reduction, University College London, UK

[7] Department of Earth Sciences, University College London, London, UK

[8] Department of Biostatistics and Bioinformatics, Rollins School of Public Health, Emory University, Atlanta, USA

[9]Woods Institute for the Environment, Stanford University, Stanford, California, USA

*Corresponding author:

Abu Mohd Naser, MBBS, MPH
Department of Environmental Health Sciences
Rollins School of Public Health | Emory University
2nd floor, CNR building, Room # 2030E
Atlanta, GA 30322, USA
email: atitu@emory.edu

For peer review only - http://bmjopen.bmj.com/site/about/guidelines.xhtml

Abstract

Introduction: Saltwater intrusion and salinization have contributed to drinking water scarcity in many coastal regions globally, leading to dependence on alternative sources for water supply. In southwest coastal Bangladesh, communities have few options but to drink brackish groundwater which has been associated with high blood pressure among the adult population, and preeclampsia and gestational hypertension among pregnant women. Managed aquifer recharge (MAR), the purposeful recharge of surface water or rainwater to aquifers to bring hydrologic equilibrium, is a potential solution for salinity problem in southwest coastal Bangladesh by creating a freshwater lens within the brackish aquifer. Our study aims to evaluate whether consumption of MAR water improves human health, particularly by reducing blood pressure among communities in coastal Bangladesh.

Methods and analysis: The study employs a stepped-wedge cluster-randomized controlled community trial design in 16 communities over five monthly visits. During each visit, we will collect data on participants' source of drinking and cooking water and measure the salinity level and electrical conductivity of household stored water. At each visit, we will also measure the blood pressure of participants' ≥ 20 years of age and pregnant women and collect urine samples for urinary sodium and protein measurements. We will use generalized linear mixed models to determine the association of access to MAR water on blood pressure of the participants.

Ethics and dissemination: The study protocol has been reviewed and approved by the Institutional Review Boards of the International Centre for Diarrheal Disease Research, Bangladesh (icddr.b). Informed written consent will be taken from all the participants. This study is funded by Wellcome Trust, UK. The study findings will be disseminated to the government partners, at research conferences, and in peer-reviewed journals.

For peer review only - http://bmjopen.bmj.com/site/about/guidelines.xhtml

Registration: (http://www.clinicaltrials.gov): NCT02746003

Strengths and limitation of the study

- This is the first study to evaluate the health impact of managed aquifer recharge in southwest coastal Bangladesh.
- The stepped-wedge trial ensures we will have counterfactual data as well as gradual access to MAR water in all communities.
- Objective measurement of exposure (drinking water salinity) and outcomes (urinary sodium and blood pressure).
- The magnitude of exposure will vary geographically and across time period. Therefore, MAR water salinity will differ across communities at a single point of time, and also for the same community at different points of time.
- Compliance of the intervention may be different across sites and for individuals of different professions.

For peer review only

For peer review only - http://bmjopen.bmj.com/site/about/guidelines.xhtml

Introduction

Background and rationale: Saltwater intrusion and salinization have increased groundwater salinity in many coastal aquifers and small islands across the world.[1-5] This is driven by a number of climatological and anthropogenic factors including global warming, increased cyclones and tidal surges, reduced river discharge, and increased groundwater abstraction in excess of recharge.[6-8] Communities in many coastal regions rely on groundwater as their main source of drinking water [9] as well as freshwater for domestic, agricultural and industrial purposes.[10] Nearly half of the world's population resides in coastal areas [3] and 10% of these live in low-lying coastal areas where surface elevation is generally ≤10 meters above mean sea level.[11] Salinization in coastal areas is expected to increase in the future because of increased groundwater withdrawal due to population and economic growth and sea-level rise.[3] As the world's population and economic activities continue to grow, groundwater supplies are progressively under threat of depletion, which increases the importance of monitoring, management and conservation of coastal freshwater aquifers.[12 13]

One approach to minimize the impact of groundwater salinization is to enhance groundwater recharge into coastal aquifers.[14] Managed aquifer recharge (MAR) is an approach to artificially promote freshwater recharge to increase storage. MAR involves infiltration of freshwater (e.g. rainwater and pond water) into aquifers to create a store of freshwater within the naturally brackish aquifer (**Figure 1**).[15-17] MAR represents a promising adaptive strategy for increasing freshwater availability and sustaining a year-round drinking water supply that is protected from evaporation, and could be resilient to tidal storms, cyclones and surface water salinity since freshwater infiltration and storage occur under confined conditions.[18]

For peer review only - http://bmjopen.bmj.com/site/about/guidelines.xhtml

The brackishness of groundwater in southwest coastal Bangladesh is caused by a combination of climatic and anthropogenic factors including sea level rise, frequent cyclones and tidal storms, and shrimp cultivation.[19-22] In the future, climate change and sea-level rise are expected to cause more cyclones, tidal surges and flood in this region that will further affect surface water and groundwater salinity.[7] In many areas in southwest coastal Bangladesh, both shallow and deep aquifers contain naturally brackish water causing acute scarcity of drinking water (**Figure 2A**).[23 24] Water salinity in southwest coastal Bangladesh follows a clear seasonal pattern ─ higher in the dry season than the wet season.[25] Salinity of water bodies builds up from October to May, peaking during February to early May. After May, salinity decreases sharply due to rainfall and increased upstream river flow. People collect and use rainwater for drinking and cooking purpose during the monsoon when precipitation is intense, but during the dry season, they generally rely on pond water or saline tube well water.[26]

The Geology Department of the University of Dhaka, in collaboration with UNICEF and the Department of Public Health Engineering (DPHE), Government of Bangladesh have piloted 20 small-scale MAR projects in three districts of southwest coastal Bangladesh to evaluate the feasibility of MAR for drinking water supply in rural communities.[27] The shallow brackish aquifer was the target storage zone that is overlain by Holocene clay aquitard of 3 to 15 meter thickness.[16] In the pilot phase, an average storage of approximately 900 m^3 of fresh water per year per site was established, sufficient to deliver 15 L of safe drinking water per day per household, which can fulfill the demand for drinking and cooking for approximately 300 people in 60-70 households during the dry season.[28 29] The second phase of the MAR project started in 2014 aiming to install 75 MAR systems in three districts of southwest coastal Bangladesh: Satkhira, Khulna and Bagerhat (**Figure 2B**). In March, 2016, 30 MAR sites were handed over to

For peer review only - http://bmjopen.bmj.com/site/about/guidelines.xhtml

the community for water consumption. The remaining 45 sites are expected to be ready by November 2016 following infiltration of water during monsoon of 2016 (June – October, 2016).

Epidemiological studies have demonstrated that high sodium intake is associated with elevated blood pressure [30-32] and other cardiovascular diseases.[33] A study conducted among the adult population residing in southwest coastal Bangladesh suggest drinking saline water was associated with high blood pressure after adjusting for personal, lifestyle and environmental factors.[34] Among study participants, the mean systolic blood pressure for those consuming water with sodium from the lowest quintile was 119.4 (SD 13.7) and from the highest sodium quintile was 126.7 mm Hg (18·0).[34] Excess sodium intake from drinking brackish water has been also associated increased gestational hypertension and preeclampsia among pregnant women in southwest coastal Bangladesh.[35 36] Mean systolic and diastolic blood pressure were higher among the pregnant women who drank tube well or pond (saline sources) water compared to those who drank rain water.[35 37] The mean systolic blood pressure of pregnant women from these areas was 102.4 mm Hg among those who drank rainwater, 112.6 mm Hg among pond water users and 119.4 mm Hg among brackish groundwater users.[35] High blood pressure during pregnancy is associated with high maternal mortality, and adverse pregnancy and fetal outcomes.[38]

There are 37 million people living in the southwest coastal region and 20 million are currently affected by drinking water salinity.[39] The estimated mean global sodium consumption is 3.95 g per day (range 2.2 to 5.5 g per day),[40] but people in southwest coastal Bangladesh consume up to 16 g of sodium per day through drinking brackish groundwater.[41] While MAR water can potentially reduce exposure to saline water, the health effects of providing access to MAR water have not been investigated. Characterizing the health consequences of shifting from

For peer review only - http://bmjopen.bmj.com/site/about/guidelines.xhtml

current drinking water supply to MAR water can assist decision-makers to assess the value of scaling up MAR. Many water interventions, for example, failed to achieve health impact, because people don't use them. There is also considerable controversy on whether reducing sodium intake improves health.[42] In addition the MAR system will also alter the intake of other cations such as calcium and magnesium that may have health impact.[43-46] Because the cost of these systems is high and their long-term feasibility an open question, clarifying the amount of health benefit, if any, is an important step that can help inform future efforts to provide water to communities faced with high levels of groundwater salinity. The human subjects committees who reviewed the study appreciated the uncertainty in bringing solutions to scale in this setting and considered it appropriate to include health measurements of participating residents to objectively evaluate the potential impact.

Objectives: Randomized controlled trials demonstrate that reduction in dietary sodium in adults decreases blood pressure among people both with and without hypertension. [47-49] Meta-analysis of randomized trials also suggest modest reduction in salt intake for four or more weeks causes significant reduction in blood pressure at the population-level. [50 51] The primary objective of the study is to assess whether access to low-salinity MAR water can reduce blood pressure of community members ≥20 years of age. Secondary objectives include whether access to MAR water can reduces urinary sodium and total protein excretion. We will evaluate water salinity, urinary sodium excretion and blood pressure so that we understand whether or not we achieved our immediate targets along the causal pathway.

For peer review only - http://bmjopen.bmj.com/site/about/guidelines.xhtml

Methods

Study design: We will implement a stepped-wedge cluster randomized controlled trial in 16 MAR communities. MAR system is a community based intervention designed to supply low-salinity water for 60-70 households in a village or community. MAR intervention cannot be implemented and randomized at individual- or household-levels. Once a community will have access to MAR water, it is difficult to withhold the access of MAR water for some households. Therefore, we will conduct a cluster randomized trial where each community will be considered as a cluster. The stepped wedge design allows communities to gradually have access to MAR water; however, the point at which their access commences will be randomly assigned.[52] In this way, each MAR site will contribute data for both the intervention and the control time periods. We will have five monthly steps in the stepped wedge trial (**Figure 3**). In the first step, none of the communities will have MAR water available for drinking and we will collect baseline information. During each subsequent month, four randomly selected communities will receive access to MAR water for drinking and cooking. In the last (fifth) monthly step, all the communities will have access to MAR drinking water.

Study settings: The study will be conducted at the community-level in three districts in southwest coastal Bangladesh ─ Khulna, Satkhira and Bagerhat ─ where 75 MAR systems have been installed. Of these, 30 systems are already in use. We will select 16 communities for study based on consultation with Dhaka University and UNICEF, Bangladesh using three criteria: communities have not started drinking MAR water, acceptable level of arsenic (<50 µg/L) in drinking water set by the government of Bangladesh,[53 54] and electrical conductivity (a measure of salinity) of water below 2000 µS/cm, an indication that the MAR system has successfully reduced salinity in the aquifer.

For peer review only - http://bmjopen.bmj.com/site/about/guidelines.xhtml

Participant eligibility criteria: The inclusion criteria will be households whose ≥ 20 year old members and pregnant women willingly agree to exclusively use MAR water during the dry season for drinking and cooking purposes. The Dhaka University team have determined the catchment area for each MAR site based on geographic distance from MAR infrastructure and have developed a list of households who expressed willingness to drink MAR water. Any ≥ 20 year old hypertensive household members will be also eligible for participation but research staff will collect information about their medication if any. Research staff will enrol post-adolescent ≥ 20 year population as an individual's response to salt intake (salt-sensitivity) increases with age[55] and adolescence is associated adrenal and nervous system maturation that may contribute to salt sensitivity.[56 57] They will approach households living near the MAR water access point. They will enroll the 28 closest households surrounding each MAR systems that meet the inclusion criteria and consent (Supplemental Information 1) to study participation. During the informed written consent process, research staff will inform each household that they will be randomly selected when to start consuming MAR water as part of the scientific process of the study. All household members ≥ 20 year of age will be eligible and enrolled in the study from the selected 28 households. In addition, research staff will enroll households that include a pregnant woman in each selected MAR catchment community irrespective of household selection, from a list of pregnant women developed by a female promoter.

Randomization and intervention delivery: We will randomly select four MAR communities to drink MAR water in each step following the first (baseline) step (**Figure 3**) by computer-generated random numbers. Randomization will be conducted by an investigator who will not be directly involved in implementation of the stepped wedge study and this will be done before commencement of the study. The study could not be blinded therefore there was no

For peer review only - http://bmjopen.bmj.com/site/about/guidelines.xhtml

concealment from the cluster participants. Although infiltration of rainwater and pond water into the brackish aquifer through the MAR systems will be ongoing for 1-2 years in each community, people will not have access to MAR water until a formal handover of MAR systems to community members. The implementers form a community management team who are responsible for maintenance of each MAR system following handover. We have synchronized the community handover with the randomization schedule in the 16 communities and our community health promoters informed participants when the MAR water will be available for consumption.

We will deploy a local trained promoter at each MAR site, who will visit these households, list members and identify pregnant women in these and other households in catchment areas of each MAR site. Promoters will encourage all household members to drink MAR water exclusively while they are at home, carry a bottle of MAR water while they go out for work and other activities, and to cook with MAR water. The community health promoters will visit households with promotional materials (e.g. flip charts) from the beginning of the study to inform household members about adverse health effects of drinking brackish water and potential benefits of drinking low-salinity MAR water. As per the randomization schedule, they will inform households when MAR water will be available for consumption.

Sample size: The sample size of the stepped wedge trial was calculated based on the effect of MAR systems to reduce systolic blood pressure of the ≥ 20 years old community members. Data from southwest coastal Bangladesh demonstrated a 7 mmHg lower mean systolic blood pressure among pregnant women who drank pond water compared to those who drank brackish water, and a 17 mmHg lower mean systolic blood pressure for pregnant women who drank rainwater compared to those who drank brackish water [35]. The mean reduction in systolic

For peer review only - http://bmjopen.bmj.com/site/about/guidelines.xhtml

blood pressure following long term modest salt reduction is 4.2 mmHg.[50] For calculations we considered a mean systolic blood pressure reduction of at least 3 mmHg among the communities who will have access to MAR water for drinking and cooking purposes compared to those who will use brackish water. The entire population under the MAR community catchment area will be considered as one cluster. Each MAR community serves approximately 300 people, constituting 60-70 households in the catchment area.[28] The mean household size in southwest coastal Bangladesh is 4.2, approximately 52% of household members are ≥ 20 years.[58 59] Since we will collect blood pressure for all household members ≥ 20 years, we estimated an average of 2.2 participants in this age group per household. We will select 28 households per cluster (28 x 2.2 = 60 people per cluster). A previously published study in Bangladesh reported standard deviation (SD) for systolic blood pressure as 13.51,[60] but we considered standard deviation of systolic blood pressure 20 to capture a greater variation of blood pressure. We calculated the sample size for the stepped wedge trial adopting the formula used by Hussey and Hughes 2007.[61] Although their formula assumed no within participant correlation over time (cross sectional design), we applied their formula for a cohort design (same participant followed up over time). The salinity problem and the MAR water quality may be influenced by seasonality over the progression of dry season and participants' blood pressure may respond differently to different level of sodium exposure in MAR drinking water. Therefore, it is important that we investigate the effect of the MAR system on the same participants over the entire dry season. Since we'll investigate the same participants in each steps, we'll have less participant-level variability and sufficient power compared to a cross-sectional design to investigate the effect of MAR systems. We initially calculated the sample size for a simple unadjusted trial (N_U), and then multiplied by the design effect for a stepped wedge trial (DE_{SW}). The sample size for a simple unadjusted trial was

For peer review only - http://bmjopen.bmj.com/site/about/guidelines.xhtml

calculated as 1,396 participants (effect size= 3, standard deviation of 20 for both groups, 80% power, and 5% Type I error). The DE_{SW} was 3.04 based on a formula provided by Hussy and Hughes therefore sample size was 1396 x 3.04. We also inflated the total sample size considering 10% loss to follow-up. We then followed the approaches of determining the number of clusters required for total sample size considering a fixed cluster size of 60.[62] We then calculated the number of steps that we need to randomize by dividing total cluster by steps. We calculated that 16 MAR communities will be required for the study with four communities randomized to access MAR water in each step (**Figure 3**).

Data collection methods: The outcomes of interest will be measured from selected participants of 16 communities irrespective of their access to MAR water during five monthly visits. The interval between two successive blood pressure measurements or two consecutive data collection visits will be at least one month for each participant. After taking informed written consent from household heads and each participant, research staff will administer a survey using a structured questionnaire to collect information on drinking water sources, household members' socio-demographic status, medical history, family history of cardiovascular diseases, medication intake and cardiovascular risk factors including smoking history and alcohol intake during the first monthly visit. At this time they will also measure participants' weight, height, waist and hip circumference and blood pressure, and collect urine samples. During subsequent monthly visits, research staff will measure blood pressure, weight and collect urine samples. If a pregnant woman is identified, research staff will measure her blood pressure, and collect urine samples at every monthly visits.

For peer review only

As part of the MAR uptake evaluation, the research staff will collect self-reported data on MAR water use for each households and whether households exclusively or intermittently use MAR water for drinking and cooking during follow-up visits. Research staff will also collect stored drinking and cooking water samples for measurement of electrical conductivity and will ask respondents the source of stored water.

Exposure assessment: During each visit, research staff will inquire about household members' reported primary water sources used for drinking and cooking purposes in last 24 hours, collect reported information on whether participants exclusively used the primary water sources and explore whether any alternative water sources were used. They will also ask about the frequency of collection and cost of primary water sources for drinking and cooking, time required to collect water, amount of collected water and when the last water was collected. Research staff will observe the presence of stored water in households, ask the sources of each container of stored water and will collect water samples that have been stored for drinking and cooking (if any) to measure the salinity level, electrical conductivity, resistivity, total dissolve solutes (TDS) and temperature of water samples using Hanna Salinity™ meter. They will also collect the MAR water from the source MAR outlet to measure the salinity level, electrical conductivity, resistivity, total dissolve solutes (TDS) and temperature of water. Research staff will collect 24 hour urine from each participant to measure the urinary sodium excretion as a proxy for sodium intake.[63 64]

Outcomes assessment: Systolic blood pressure of the ≥ 20 year old population and pregnant women is the primary outcome of this study. Secondary outcomes will be diastolic blood pressure, mean arterial pressure, and pulse pressure (**Table 1**). The different components of blood pressure are independent cardiovascular risk factors associated with sodium intake,[65-69]

For peer review only - http://bmjopen.bmj.com/site/about/guidelines.xhtml

and indicate future risk for different cardiovascular diseases.[70] High systolic blood pressure puts more stress on the vascular wall and is strongly associated with risk for future intracerebral haemorrhage, subarachnoid haemorrhage, angina, myocardial infarction and peripheral vascular diseases.[70] Raised diastolic blood pressure is associated with aortic and thoracic aneurysm.[70] Some effects of high sodium intake are independent of high systolic and diastolic blood pressure such as arterial stiffness and left ventricular mass ─ both of these are independent predictor of future cardiovascular diseases. High pulse pressure (difference between systolic and diastolic blood pressure) is associated with increased arterial stiffness,[66 68] and high mean arterial pressure (diastolic blood pressure plus one-third of systolic blood pressure) and high pulse pressure is independently associated with increased left ventricular mass.[66 68]

We will also measure the creatinine adjusted protein excretion of all participants as tertiary outcomes (**Table 1**). Proteinuria is a biomarker for future risk of cardiovascular diseases,[71-74] and is associated with the pathogenesis of cardiovascular diseases, including hypertension,[75 76] chronic kidney disease,[77] myocardial ischemia,[78] carotid artery thickness,[79 80] left ventricular hypertrophy,[81 82] hyperlipidemia,[83] atherosclerosis,[84] and coronary artery calcification.[72 80 85 86] Reduced salt intake for four weeks has been associated with decreased proteinuria in blinded randomized controlled trial.[87] Preeclampsia is associated with high maternal mortality and adverse pregnancy and fetal outcomes [38] and is characterized by high blood pressure and proteinuria after 20 weeks of pregnancy.[88]

Blood pressure measurement: Blood pressure will be measured for all ≥20 year old participants and pregnant women during each step. Research staff will use a Omron® HEM–907 blood pressure device, which is comparable to the gold standard mercury sphygmomanometers in terms of measurement and meets the Association for the Advancement of Medical

For peer review only - http://bmjopen.bmj.com/site/about/guidelines.xhtml

Instrumentation's (AAMI) criteria.[89] Blood pressure will be measured following the guidelines described by Pickering et al. 2005 and Giorgini et al. 2014.[90 91] Caffeine (tea, coffee, carbonated beverages), eating, heavy physical activities and smoking will be proscribed for 30 minutes prior to measuring blood pressure. Participants will rest for 5 minutes sitting on a chair keeping their arm supported. An appropriate sized cuff and calibrated instrument will be used for different age groups and the blood pressure instrument will be positioned at heart level. Blood pressure will be measured three times; first left arm, then right arm, then again left arm. Both systolic and diastolic blood pressure will be recorded from all measurements. The arithmetic mean of three systolic blood pressure measurements will be used as the primary outcome. However, if a systolic blood pressure measurement differs by 10% from the other measurements, that measurement will be excluded when calculating the arithmetic mean systolic blood pressure.

Biomarker measurements: Field research staff will instruct the participants' to collect 24 hours urine sample during each household visit. The volume of the urine samples will be noted at household level, and a sample of 25 ml urine will be collected and transported to the field laboratory at 2-8° C within 6 hours of collection for processing, analysis and storage. Aliquots of each participant's urine sample will be made for biochemical and electrolyte measurements. Urinary creatinine concentration will be measured by a colorimetric method (Jaffe reaction) using a semi-auto biochemistry analyzer (Evolution 3000, BSI, Italy). Urinary total protein will be estimated using a light sensitive colored reagent (Randox.UK). We will use the direct Ion Selective Electrode (ISE) method for urinary sodium measurements using a semi-auto electrolyte analyzer (Biolyte2000, Bio-care Corporation, Taiwan). We will measure the uric acid concentration in blood by an enzymatic colorimetric method (Evolution 3000, BSI, Italy). We will perform routine Quality Control for all tests using standard quality control reagents

For peer review only - http://bmjopen.bmj.com/site/about/guidelines.xhtml

(Bio-Rad Laboratories, USA) and 5% of samples will be cross checked at the International Centre for Diarrhoeal Disease Research, Bangladesh (icddr,b) central laboratory, in Dhaka.

Data management and monitoring: The data collection instrument will be programmed and field research staff will use hand held computers to collect data. Appropriate range values and data values will be programmed to minimize data entry errors. The dataset downloaded from handheld devices will be cleaned and checked by the site investigators. All laboratory data will be double entered. Data will be stored in icddr,b's data repository system, in compliance with the system's requirements and will be publicly available after analyzing the primary result.

The research staff will be trained for identifying adverse events such as hypertension and hypertensive disorders in pregnancy. They will report to the investigators following identification of these patients and the investigators will assess whether these adverse events need to be reported to icddr,b's Ethical Review Committee.

Statistical methods: We will conduct an intention-to-treat analysis for the primary analysis. For the primary analysis we will assess whether access to MAR water reduce the systolic blood pressure (continuous outcome) of the >20 years old community member. We will use generalized linear mixed models with appropriate links for the primary analysis considering random effects for community, households and participants, and a fixed effects of steps or visits. We will adjust the effect of MAR systems on blood pressure for age, sex, weight and height, personal, and socioeconomic factors **(Table 2).** We will inspect the missing data patterns and use multiple imputation with chained equations to jointly impute data on missing exposure and confounders to preserve an unbiased association estimate if the data are missing at random conditional on measured variables. We will also conduct subgroup analyses among the households that adhere with the MAR intervention and that exclusively use MAR water. In

For peer review only - http://bmjopen.bmj.com/site/about/guidelines.xhtml

secondary analyses, we will consider the association of drinking MAR water with diastolic blood pressure, pulse pressure, mean arterial pressure, and urinary protein concentrations. Since pregnant women's physiology is different from other adults, we will conduct separate analysis for this group. We will conduct generalized linear mixed models with appropriate links to assess whether access to water from MAR systems reduce blood pressure and urinary protein among the pregnant women. We'll consider the random effects for communities and pregnant women and fixed effects for steps and gestational weeks to determine the health effects of access to MAR water on pregnant women's blood pressure and urinary protein. If any women in the enrolled households become pregnant during the study, we will collect their blood pressure data during routine visits. However, since cardiovascular changes occur after eight weeks of gestation, we will include them in pregnant women analysis from the eighth week of gestation.

Ethics: Informed written consent will be taken from all participants and household heads. Consent will be also taken for ancillary studies and future use of specimens collected from study participants. This study protocol has been reviewed and approved by the icddr,b Ethical Review Committee. Approval will be taken for any addition or modification of the protocol from icddr,b Ethical Review Committee. If research staff members identify hypertensive patients or cases of hypertension during pregnancy, they will refer patients to the local government health facilities for further management. In addition, research staff will train pregnant women and family members to recognize the danger signs during pregnancy, and will also instruct them where to seek medical care if such danger signs appear. During monthly household visits research staff will encourage pregnant women to attend prenatal visits. All dataset will be anonymous without the personal identifiers and participants' privacy will be maintained during data storage, analysis and dissemination.

For peer review only - http://bmjopen.bmj.com/site/about/guidelines.xhtml

Dissemination: Study findings will be shared with the Department of Public Health Engineering (DPHE), Department of Environment of the government of Bangladesh and other partner NGOs working in southwest coastal Bangladesh for access to safe drinking water. We will discuss the scope and limitations of the MAR system to address the demand of safe drinking water based on findings from this study. We will be submitting abstracts to international conferences for dissemination to the international audience working on safe water. We will develop manuscripts and submit to peer-reviewed journals to publish our research.

For peer review only

For peer review only - http://bmjopen.bmj.com/site/about/guidelines.xhtml

Discussion

This will be the first study to assess the health impact of an environmental intervention to reduce groundwater salinity in southwest coastal Bangladesh. Our study has several strengths. The stepped-wedge trial ensures that we will have counterfactual data as well as gradual access to MAR water in all communities. In a stepped-wedge design, treatment effect of an intervention can be estimated from between- and within-cluster comparisons as participants will act as their own control, compared to only between-cluster comparison in a parallel cluster randomized design.[61]

We explored other study designs such as non-randomized study. As per suggestion of icddr,b's IRB, we conducted a pilot study in four communities (two MAR communities and two non-MAR communities) during May-August 2016. Pilot study findings suggested that because of the heterogeneity in village level hydrogeological conditions and in participation by community residents with different characteristics, non-randomized quasi-experimental studies would expected to generate unequal distribution of confounders that would undermine scientific inference. The objective of the pilot study was to pre-test our data collection instruments laboratory protocols prior commencement of the main study We identified unequal distributions of several important observed confounders across the communities such as socio-economic status, and household characteristics such as supply of electricity and ownership refrigerator. We identified that some covariates such as supply of electricity was perfectly predictive of communities receiving MAR interventions. We were unable to derive satisfactory matching of MAR intervention and control households after principal component analysis. The findings highlighted the importance of a randomized trial to evaluate the health effects of MAR systems

For peer review only - http://bmjopen.bmj.com/site/about/guidelines.xhtml

since there are many observed and unobserved household and community-level and hydrogeological confounders that may influence blood pressure.

We also explored the possibilities of parallel group randomized trial but several programmatic issues barred us to implement a parallel group cluster randomized trial. The implementers of MAR systems (UNICEF Bangladesh and University of Dhaka) installed the 75 new sites in different stages and planned to allow communities to access these over three dry seasons. The implementers informed us that 20-25 MAR sites may be suitable for community access during our study period and they wanted to allow all communities to drink MAR water in the same season after satisfactory reduction in salinity. Therefore, a parallel group cluster randomized trial was not suitable as we could not randomly select control sites and postpone the community to drink water for the dry season. We believe that the stepped-wedge trial design was appropriate to obtain counterfactual data as well as fulfilling the programmatic need.

We will use the same instruments for data collection and outcome measurements for all steps for exposed and unexposed communities, which will mitigate bias in data collection. Measurement of water source salinity and urinary sodium concentration will explain the biologically plausible effect of drinking MAR water on blood pressure. All outcomes planned to measure in the study are objective outcomes that will reduce the risk of reporting bias. Collection of detailed exposure and co-variate data will help in determining a valid association between drinking MAR water and health benefits. We will have several outcome variables and biomarkers that will ensure a comprehensive health benefit evaluation of access to MAR water.

The study has several limitations. We will be unable to control salinity level of drinking water across the 16 MAR communities due to different hydro-geological conditions and volume

For peer review only - http://bmjopen.bmj.com/site/about/guidelines.xhtml

of infiltrated water across the aquifers. Although the initial electrical conductivity of MAR water will be < 2000 µS/cm, electrical conductivity is likely to vary across different MAR communities with the intensification of dry season. Therefore, MAR water salinity will differ across communities at a single point of time, and also for the same community at different points of time. This will result in different versions of exposures (i.e. access to MAR water with different salinity-levels) that may affect different responses for some participants depending on the version of exposure they will receive.[92 93] To account this, we will specify the versions of exposure by measuring the salinity level of MAR water available in participants' households and interpret the response as unit change of salinity at different MAR water salinity-levels. As compliance of drinking MAR water may be different across communities despite active encouragement by promoters, one problem of the intention to treat analysis is that if the proportions of participants who will always drink MAR water is low compared to those who will not, the potentially greater effect of MAR intervention on blood pressure of fully participating individuals may be washed out by the smaller effect of those who will not comply. To account for this, as a secondary analysis, we will conduct "instrumental variable" (IV) analyses by jointly running two regression models: a regression model for predicting urinary sodium excretion by drinking water salinity, and a regression model for blood pressure prediction given the urinary sodium excretion. The assumption of instrumental variable analyses is fulfilled in our context as the exogenous variable (instrumental variable) can only affect outcome variable (blood pressure) through influencing the endogenous variable (urinary sodium excretion).[94] If MAR water has lower-salinity than other water sources and if participants always drink MAR water, their urinary sodium excretion will be low compared to those who will not fully participate.

For peer review only - http://bmjopen.bmj.com/site/about/guidelines.xhtml

Acknowledgements: The study has been funded by Wellcome Trust, UK, Our Planet, Our Health Award. Wellcome Trust reviewed and approved the design as the condition of providing funding but will not have a role in data collection, data analysis, data interpretation, or writing of the report. The study was implemented by International Centre for Diarrhoeal Disease Research, Bangladesh (icddr,b). icddr,b acknowledges with gratitude the commitment of Wellcome Trust, UK to its research efforts. icddr,b is also grateful to the Governments of Bangladesh, Canada, Sweden and the UK for providing core/unrestricted support. Dr. Gribble is supported by funding from the National Institute for Environmental Health Sciences to the Emory Health and Research Exposome Center (P30 ES019776). The authors would like to thank Dr. Karla Hemming, Institute of Applied Health Research, University of Birmingham for her input in design and sample size calculation. We are grateful to the Dhaka University/UNICEF field team for their assistance in providing preliminary data about the readiness of MAR sites.

Contributors: SPL and AMN developed the study concept. AMN, SPL, LU, KMA, and SD have developed the study design. MR, MOG, TFC and HHC reviewed the epidemiological study design. KMA, MS, WB, SS and MNU provided input in environmental and biological sample collection and analysis. HHC and MOG provided input in statistical analysis. AMN drafted and all author reviewed the manuscript.

Competing interest: None

Funding: Wellcome Trust, UK (Grant # 106871/Z/15/Z).

Data sharing statement: The anonymous dataset without personal identifiers will be publicly available following publication of the primary trial results.

For peer review only - http://bmjopen.bmj.com/site/about/guidelines.xhtml

References-

1. Van Weert F, van der Gun J, Reckman J. Global overview of saline groundwater occurrence and genesis. International Groundwater Resources Assessment Centre 2009.
2. Vengosh A, Rosenthal E. Saline groundwater in Israel: its bearing on the water crisis in the country. Journal of Hydrology 1994;**156**(1):389-430.
3. Post V. Fresh and saline groundwater interaction in coastal aquifers: Is our technology ready for the problems ahead? Hydrogeology Journal 2005;**13**(1):120-23.
4. Holman IP. Climate change impacts on groundwater recharge-uncertainty, shortcomings, and the way forward? Hydrogeology journal 2006;**14**(5):637-47.
5. Ferguson G, Gleeson T. Vulnerability of coastal aquifers to groundwater use and climate change. Nature Climate Change 2012;**2**(5):342-45.
6. Mall R, Gupta A, Singh R, et al. Water resources and climate change: an Indian perspective. Current Science 2006;**90**(12):1610-26.
7. Taylor RG, Scanlon B, Döll P, et al. Ground water and climate change. Nature Climate Change 2013;**3**(4):322-29.
8. Changming L, Jingjie Y, Kendy E. Groundwater exploitation and its impact on the environment in the North China Plain. Water International 2001;**26**(2):265-72.
9. Counts HB, Donsky E. *Salt-water Encroachment Geology and Ground-water Resources of Savannah Area Georgia and South Carolina*: US Government Printing Office, 1963.
10. Barlow PM. *Ground Water in Fresh Water-salt Water Environments of the Atlantic*: Geological Survey (USGS), 2003.
11. McGranahan G, Balk D, Anderson B. The rising tide: assessing the risks of climate change and human settlements in low elevation coastal zones. Environment and urbanization 2007;**19**(1):17-37.
12. Villholth KG. Integrated groundwater use and management in vulnerable coastal zones of Asia-Pacific. Groundwater in the Coastal Zones of Asia-Pacific: Springer, 2013:317-42.
13. Morris BL, Lawrence AR, Chilton P, et al. Groundwater and its susceptibility to degradation: a global assessment of the problem and options for management. 2003.
14. Dillon P. Future management of aquifer recharge. Hydrogeology journal 2005;**13**(1):313-16.
15. Bouwer H. Artificial recharge of groundwater: systems, design, and management. Hydraulic design handbook McGraw-Hill, New York 1999:24.1-24.44.
16. Ahmed K, Sultana S, Groen K, et al. DEVELOPMENT OF OPTIMAL DESIGN OF AQUIFER STORAGE AND RECOVERY FOR PROVIDING SAFE WATER TO VULNERABLE COMMUNITIES OF COASTAL BANGLADESH. International conference on Climate Change in relation to Water and Environment (I3CWE-2015). Department of Civil Engineering, DUET - Gazipur, Bangladesh, 2015:1-8.
17. Dillon P, Toze S, Page D, et al. Managed aquifer recharge: rediscovering nature as a leading edge technology. Water Science and Technology 2010;**62**(10):2338.
18. Groundwater and Climate Change: Challenges and Possibilities 2008 [Available from: http://www.geus.dk/program-areas/water/denmark/rapporter/groundwater_and_%20climate_change_071108.pdf.
19. Allison MA, Khan SR, Goodbred SL, et al. Stratigraphic evolution of the late Holocene Ganges-Brahmaputra lower delta plain. Sedimentary Geology 2003;**155**(3-4):317-42.
20. Mirza MMQ. Diversion of the Ganges water at Farakka and its effects on salinity in Bangladesh. Environmental management 1998;**22**(5):711-22.
21. Karim MR. Brackish-water shrimp cultivation threatens permanent damage to coastal agriculture in Bangladesh. Environment and livelihoods in tropical coastal zones: managing agriculture-fishery-aquaculture conflicts 2006;**2**:61-71.

For peer review only - http://bmjopen.bmj.com/site/about/guidelines.xhtml

22. Mahmuduzzaman M, Ahmed ZU, Nuruzzaman A, et al. Causes of Salinity Intrusion in Coastal Belt of Bangladesh. International Journal of Plant Research 2014;**4**(4A):8-13.
23. Bahar MM, Reza MS. Hydrochemical characteristics and quality assessment of shallow groundwater in a coastal area of Southwest Bangladesh. Environmental Earth Sciences 2010;**61**(5):1065-73.
24. Zahid A, Jahan K, Ali MH, et al. Distribution of Groundwater Salinity and Its Seasonal Variability in the Coastal Aquifers of Bengal Delta, in: Zahid et al. (Eds.), Impact of Climate Change on Socio-economic Conditions of Bangladesh. German Academic Exchange Service (DAAD) 2013:170-93.
25. Changes of the seasonal salinity distribution at the Sundarbans coast due to impact of climate change. 4th International Conference on Water & Flood Management (ICWFM); 2013.
26. Islam MA, Sakakibara H, Karim MR, et al. Potable water scarcity: options and issues in the coastal areas of Bangladesh. Journal of water and health 2013;**11**(3):532-42.
27. Acacia Water DU. Action reserach: Creating fresh water bubbles in Brackish aquifer 2011 [Available from: http://www.google.com.bd/url?sa=t&rct=j&q=creating+Fresh+water+bubbles+in+Brackish+aquifers&source=web&cd=1&cad=rja&ved=0CCcQFjAA&url=http%3A%2F%2Facaciawater.com%2Fsite%2Fmedia%2Fprojecten%2FFlyer_MAR_BD.pdf&ei=Q6gHUZrHDKe0iQfdjoCADg&usg=AFQjCNHc5fhWh5Nk2v-jzz0s6PTsztFAqg&bvm=bv.41524429,d.aGc.
28. UNICEF. Underground fresh water storage A practical solution to increase water security in saline deltas 2014 [Available from: http://www.spaarwater.com/content/27227/download/clnt/57128_Underground_fresh_water_storage_-_a_practical_solution_to_increase_water_security_in_saline_deltas.pdf.
29. Sultana S, Ahmed K, Mahtab-Ul-Alam S, et al. Low-Cost Aquifer Storage and Recovery: Implications for Improving Drinking Water Access for Rural Communities in Coastal Bangladesh. Journal of Hydrologic Engineering 2014.
30. Alderman MH. Salt, blood pressure, and human health. Hypertension 2000;**36**(5):890-3.
31. Cutler JA, Follmann D, Allender PS. Randomized trials of sodium reduction: an overview. Am J Clin Nutr 1997;**65**(2 Suppl):643S-51S.
32. Midgley JP, Matthew AG, Greenwood CM, et al. Effect of reduced dietary sodium on blood pressure: a meta-analysis of randomized controlled trials. JAMA 1996;**275**(20):1590-7.
33. Strazzullo P, D'Elia L, Kandala N-B, et al. Salt intake, stroke, and cardiovascular disease: meta-analysis of prospective studies. Bmj 2009;**339**.
34. Scheelbeek PF, Chowdhury MA, Haines A, et al. High concentrations of sodium in drinking water and raised blood pressure in coastal deltas affected by episodic seawater inundations. The Lancet Global Health 2016;**4**:S18.
35. Khan AE, Scheelbeek PFD, Shilpi AB, et al. Salinity in Drinking Water and the Risk of (Pre) Eclampsia and Gestational Hypertension in Coastal Bangladesh: A Case-Control Study. 2014.
36. Khan AE, Ireson A, Kovats S, et al. Drinking Water Salinity and Maternal Health in Coastal Bangladesh: Implications of Climate Change. Environ Health Perspect 2011.
37. Scheelbeek PF, Khan AE, Mojumder S, et al. Drinking Water Sodium and Elevated Blood Pressure of Healthy Pregnant Women in Salinity-Affected Coastal Areas. Hypertension 2016;**68**(2):464-70.
38. Brown MA, Lindheimer MD, de Swiet M, et al. The classification and diagnosis of the hypertensive disorders of pregnancy: statement from the International Society for the Study of Hypertension in Pregnancy (ISSHP). Hypertension in pregnancy 2001;**20**(1):ix-xiv.
39. Dasgupta S, Kamal FA, Khan ZH, et al. River salinity and climate change: evidence from coastal Bangladesh. World Bank Policy Research Working Paper 2014(6817).
40. Mozaffarian D, Fahimi S, Singh GM, et al. Global sodium consumption and death from cardiovascular causes. New England Journal of Medicine 2014;**371**(7):624-34.

For peer review only - http://bmjopen.bmj.com/site/about/guidelines.xhtml

41. Khan AE, Ireson A, Kovats S, et al. Drinking Water Salinity and Maternal Health in Coastal Bangladesh: Implications of Climate Change. Environ Health Perspect.
42. Mente A, O'Donnell M, Rangarajan S, et al. Associations of urinary sodium excretion with cardiovascular events in individuals with and without hypertension: a pooled analysis of data from four studies. The Lancet 2016;**388**(10043):465-75.
43. Kinniburgh D, Smedley P. Arsenic contamination of groundwater in Bangladesh. Kinniburgh, D G and Smedley, P L (Editors), British Geological Survey Report WC/00/19, British Geological Survey, Keyworth. British Geological Survey 2001.
44. Sauvant M-P, Pepin D. Drinking water and cardiovascular disease. Food and chemical toxicology 2002;**40**(10):1311-25.
45. Marx A, Neutra RR. Magnesium in drinking water and ischemic heart disease. Epidemiologic reviews 1997;**19**(2):258-72.
46. BiÖrck G, BostrÖm H, WidstrÖm A. On the Relationship Between Water Hardness and Death Rate in Cardiovascular Diseases1. Acta medica Scandinavica 1965;**178**(2):239-52.
47. He FJ, MacGregor GA. Effect of longer-term modest salt reduction on blood pressure. Cochrane Database Syst Rev 2004;**3**.
48. Organization WH. *Guideline: Sodium intake for adults and children*: World Health Organization, 2012.
49. Graudal NA, Hubeck-Graudal T, Jurgens G. Effects of low sodium diet versus high sodium diet on blood pressure, renin, aldosterone, catecholamines, cholesterol, and triglyceride. Cochrane Database Syst Rev 2011;**11**.
50. He FJ, Li J, MacGregor GA. Effect of longer term modest salt reduction on blood pressure: Cochrane systematic review and meta-analysis of randomised trials. 2013.
51. He F, MacGregor G. Effect of modest salt reduction on blood pressure: a meta-analysis of randomized trials. Implications for public health. Journal of human hypertension 2002;**16**(11):761-70.
52. De Allegri M, Pokhrel S, Becher H, et al. Step-wedge cluster-randomised community-based trials: an application to the study of the impact of community health insurance. Health Res Policy Syst 2008;**6**:10.
53. UNICEF B. Bangladesh National Drinking Water Quality Survey of 2009 2009 [Available from: https://www.unicef.org/bangladesh/BNDWQS_2009_web.pdf.
54. Ministry of Environment and Forest, Government of the People's Republic of Bangladesh. The Environment Conservation Rules, 1997 [Available from: http://extwprlegs1.fao.org/docs/pdf/bgd19918.pdf.
55. Luft FC, Miller JZ, Grim CE, et al. Salt sensitivity and resistance of blood pressure. Age and race as factors in physiological responses. Hypertension 1991;**17**(1 Suppl):I102.
56. Orchard T, Rodgers M, Hedley A, et al. Changes in blood lipids and blood pressure during adolescence. Br Med J 1980;**280**(6231):1563-67.
57. Fujita T. Mechanism of salt-sensitive hypertension: focus on adrenal and sympathetic nervous systems. Journal of the American Society of Nephrology 2014;**25**(6):1148-55.
58. BBS. POPULATION AND HOUSING CENSUS 2011 [Available from: http://203.112.218.66/WebTestApplication/userfiles/Image/BBS/Socio_Economic.pdf.
59. NIPORT. Bangladesh Demographic and Health Survey: Preliminary Report. Dhaka, 2011.
60. Khan SH, Talukder SM, Zaman MM. Non-communicable Disease Prevention Intervention in Production Sector of Bangladesh: Findings and Lessons Learnt. 13th ASCON. icddr,b, Dhaka, 2011.
61. Hussey MA, Hughes JP. Design and analysis of stepped wedge cluster randomized trials. Contemporary clinical trials 2007;**28**(2):182-91.

For peer review only - http://bmjopen.bmj.com/site/about/guidelines.xhtml

62. Hemming K, Taljaard M. Sample size calculations for stepped wedge and cluster randomised trials: a unified approach. Journal of clinical epidemiology 2016;**69**:137-46.
63. McLean RM. Measuring population sodium intake: a review of methods. Nutrients 2014;**6**(11):4651-62.
64. Brown IJ, Dyer AR, Chan Q, et al. Estimating 24-hour urinary sodium excretion from casual urinary sodium concentrations in western populations the INTERSALT study. American journal of epidemiology 2013;**177**(11):1180-92.
65. Bednarski A, Stolarz-Skrzypek K, Kielbasa G, et al. Pulse pressure in relation to 24-hour urinary sodium excretion in a sample of high-salt intake population. Artery Research 2015;**12**:5.
66. Staessen J, Bulpitt CJ, Thijs L, et al. Pulse Rate and Sodium Intake Interact to Determine Blood Pressure A Population Study. American journal of hypertension 1991;**4**(2 Pt 1):107-12.
67. Weir MR, Dengel DR, Behrens MT, et al. Salt-induced increases in systolic blood pressure affect renal hemodynamics and proteinuria. Hypertension 1995;**25**(6):1339-44.
68. Du Cailar G, Mimran A. Non-pressure-related effects of dietary sodium. Current hypertension reports 2009;**11**(1):12-17.
69. Cheung B, Ho S, Cheung A, et al. Diastolic blood pressure is related to urinary sodium excretion in hypertensive Chinese patients. QJM 2000;**93**(3):163-68.
70. Rapsomaniki E, Timmis A, George J, et al. Blood pressure and incidence of twelve cardiovascular diseases: lifetime risks, healthy life-years lost, and age-specific associations in 1· 25 million people. The Lancet 2014;**383**(9932):1899-911.
71. Kramer H, Jacobs DR, Bild D, et al. Urine albumin excretion and subclinical cardiovascular disease. Hypertension 2005;**46**(1):38-43.
72. Weir MR. Microalbuminuria and cardiovascular disease. Clinical Journal of the American Society of Nephrology 2007;**2**(3):581-90.
73. Luft FC, Agrawal B. Microalbuminuria as a predictive factor for cardiovascular events. Journal of cardiovascular pharmacology 1999;**33**:S11-S15.
74. Bigazzi R, Bianchi S, Baldari D, et al. Microalbuminuria in salt-sensitive patients. A marker for renal and cardiovascular risk factors. Hypertension 1994;**23**(2):195-99.
75. Bianchi S, Bigazzi R, Campese VM. Microalbuminuria in essential hypertension: significance, pathophysiology, and therapeutic implications. American journal of kidney diseases 1999;**34**(6):973-95.
76. Agrawal B, Berger A, Wolf K, et al. Microalbuminuria screening by reagent strip predicts cardiovascular risk in hypertension. Journal of hypertension 1996;**14**(2):223-28.
77. Sarafidis PA, Bakris GL. Microalbuminuria and chronic kidney disease as risk factors for cardiovascular disease. Nephrology Dialysis Transplantation 2006;**21**(9):2366-74.
78. Diercks G, Van Boven A, Hillege H, et al. Microalbuminuria is independently associated with ischaemic electrocardiographic abnormalities in a large non-diabetic population. The PREVEND (Prevention of REnal and Vascular ENdstage Disease) study. European heart journal 2000;**21**(23):1922-27.
79. Bigazzi R, Bianchi S, Nenci R, et al. Increased thickness of the carotid artery in patients with essential hypertension and microalbuminuria. Journal of human hypertension 1995;**9**(10):827-33.
80. Mykkänen L, Zaccaro DJ, O'Leary DH, et al. Microalbuminuria and carotid artery intima-media thickness in nondiabetic and NIDDM subjects The Insulin Resistance Atherosclerosis Study (IRAS). Stroke 1997;**28**(9):1710-16.
81. Pedrinelli R, Di Bello V, Catapano G, et al. Microalbuminuria is a marker of left ventricular hypertrophy but not hyperinsulinemia in nondiabetic atherosclerotic patients. Arteriosclerosis, Thrombosis, and Vascular Biology 1993;**13**(6):900-06.

For peer review only - http://bmjopen.bmj.com/site/about/guidelines.xhtml

82. Assadi F. Relation of left ventricular hypertrophy to microalbuminuria and C-reactive protein in children and adolescents with essential hypertension. Pediatric cardiology 2008;**29**(3):580-84.
83. Campese VM, Bianchi S, Bigazzi R. Association between hyperlipidemia and microalbuminuria in essential hypertension. Kidney International 1999;**56**:S10-S13.
84. Jensen JS, Borch-Johnsen K, Jensen G, et al. Atherosclerotic risk factors are increased in clinically healthy subjects with microalbuminuria. Atherosclerosis 1995;**112**(2):245-52.
85. Klausen K, Borch-Johnsen K, Feldt-Rasmussen B, et al. Very low levels of microalbuminuria are associated with increased risk of coronary heart disease and death independently of renal function, hypertension, and diabetes. Circulation 2004;**110**(1):32-35.
86. Currie G, Delles C. Proteinuria and its relation to cardiovascular disease. International journal of nephrology and renovascular disease 2013;**7**:13-24.
87. Swift PA, Markandu ND, Sagnella GA, et al. Modest salt reduction reduces blood pressure and urine protein excretion in black hypertensives A randomized control trial. Hypertension 2005;**46**(2):308-12.
88. Leeman L, Fontaine P. Hypertensive disorders of pregnancy. American family physician 2008;**78**(1):93-100.
89. Ostchega Y, Zhang G, Sorlie P, et al. Blood Pressure Randomized Methodology Study Comparing Automatic Oscillometric and Mercury Sphygmomanometer Devices: National Health and Nutrition Examination Survey, 2009–2010. National Health Statistics Reports, 2012.
90. Pickering T, Hall J, Appel L, et al. Blood pressure measurement in humans: A statement for professionals from the subcommittee of professional and public education of the American Heart Association Council on High Blood Pressure Research. J Am Heart Assoc 2005;**45**:142-61.
91. Giorgini P, Weder AB, Jackson EA, et al. A review of blood pressure measurement protocols among hypertension trials: implications for “evidence-based” clinical practice. Journal of the American Society of Hypertension 2014;**8**(9):670-76.
92. Schwartz S, Gatto NM, Campbell UB. Extending the sufficient component cause model to describe the Stable Unit Treatment Value Assumption (SUTVA). Epidemiologic Perspectives & Innovations 2012;**9**(1):3.
93. VanderWeele TJ. Concerning the consistency assumption in causal inference. Epidemiology 2009;**20**(6):880-83.
94. Arellano M, Bover O. Another look at the instrumental variable estimation of error-components models. Journal of econometrics 1995;**68**(1):29-51.

For peer review only

For peer review only - http://bmjopen.bmj.com/site/about/guidelines.xhtml

Table 1: Study outcomes including normal ranges of blood pressure and biomarkers

Outcomes	Study population		Measures	Normal range/calculation
	≥ 20 years old	Pregnant women		
Primary outcome: Systolic blood pressure	Yes	Yes	Systolic blood pressure	90 -140 mmHg
Secondary outcomes: Diastolic blood pressure	Yes	Yes	Diastolic blood pressure	60 -90 mmHg
Mean arterial pressure	Yes	Yes	Mean arterial pressure	Diastolic blood pressure + 1/3 systolic blood pressure
Pulse pressure	Yes	Yes	Pulse pressure	Systolic blood pressure – diastolic blood pressure
Tertiary outcomes: Urinary sodium excretion	Yes	Yes	Urinary sodium-creatinine ratio	40-220 mEq/L/24 hours
Proteinuria	Yes	Yes	Urinary protein-creatinine ratio	< 0.11 mg/mg

For peer review only

Table 2: Key variables to be used for assessing the health impact of access to MAR water

**Outcomes**	**Exposures**	**Confounders**	**Co-variates**	**Moderators**
Systolic blood pressure	Access to MAR water	Sex	Age	Drinking/cooking from another water sources
Diastolic blood pressure	Drinking water salinity	Socio-economic status	Weight	
Pulse pressure	Cooking water salinity	Education	Height	
Mean arterial pressure			Waist circumference	
	Drinking water sources			
	Cooking water sources			
Urinary total protein			Mid upper arm circumference	
Urinary sodium excretion				
			Adding table salt	
			Adding salt for cooking	
			Exercise level	
			Smoking status	
			Use of betel nuts	
			Stress	
			Diet	
			Urinary creatinine	

For peer review only

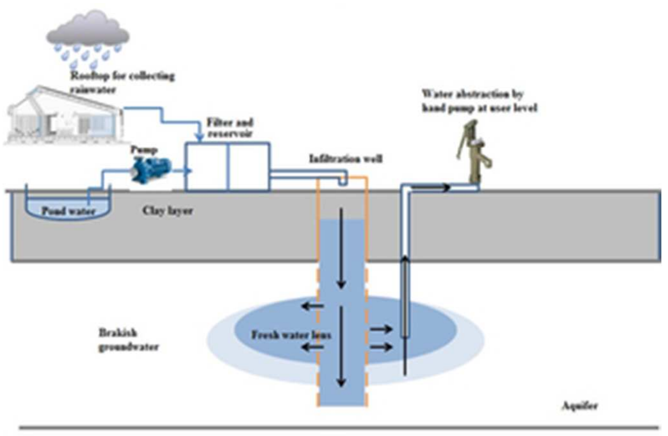

Figure 1: Schematic diagram of the MAR systems in southwest coastal Bangladesh

27x18mm (300 x 300 DPI)

For peer review only

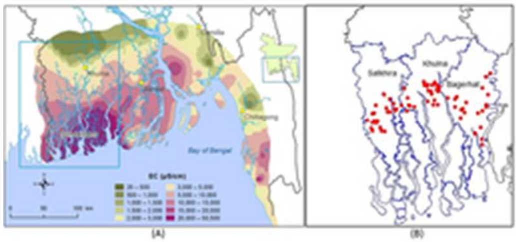

Figure 2: A) Groundwater salinity (measured as EC: electrical conductivity) distribution in coastal Bangladesh (data source: Bangladesh Water Development Board) and B) location of the 75 MAR sites in southwest coastal region illustrated by red dots [data source: Professor Kazi Matin Uddin Ahmed].

21x10mm (300 x 300 DPI)

peer review only

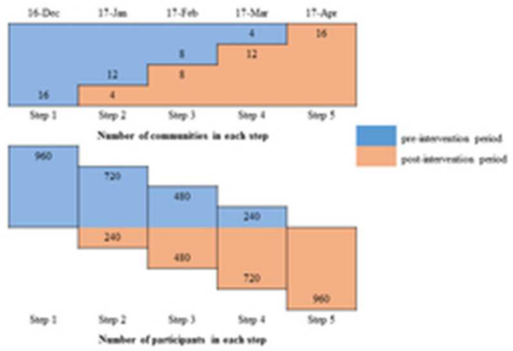

Figure 3: Timeline of blood pressure measurement with number of MAR communities and participants in pre-intervention (blue color) and post-intervention (brown color) time period in different steps

22x15mm (300 x 300 DPI)

For peer review only

Supplemental Information 1A: Consent from household head

Protocol Title: Health impacts of a climate change adaptation strategy to address drinking-water salinity in coastal Bangladesh

Investigator's name: Dr. Md. Mahbubur Rahman

Organization: **International Centre for Diarrhoeal Disease Research, Bangladesh (ICDDR,B)**

Purpose of the research

Hello (Assalamualaikum/Nomoshkar). My name is ________ and I work with the ICDDR,B (Cholera Hospital) in Dhaka. You are aware that a Managed aquifer recharge scheme has already been installed in your community that would like reduce the salinity problem in your drinking water. You may also know that drinking saline water is associated with hypertension. We will conduct a pilot study to understand the mean blood pressure of the household members who are drinking water from MAR.

Background (brief introduction of the issue and the need for/ importance of the research)
Salinity in drinking water is a big problem in your community and in many other communities in the south-western coastal region of Bangladesh. Previous research has demonstrated that drinking saline water caused high blood pressure among the adult population and gestational hypertension among the pregnant women. Hypertension risk many other cardiovascular diseases and gestational hypertension also causes adverse pregnancy and fetal outcomes. Managed aquifer recharge (MAR) is a promising sustainable intervention that can reduce salinity in groundwater. Moreover, MAR will not be affected by the cyclone and tidal surges. There is a potential to scale-up MAR across the south-western coastal region if we can demonstrate that drinking MAR water provide health benefits and do not pose any risk to human health. Now we would like to conduct a pilot study where we will measure the mean blood pressure of your household members and the salt concentration of their urine.

Why invited to participate in the study?
You're invited to participate in our study because your household is located in such an area where a new MAR site has been installed. Rain water and pond water is being infiltrated into the groundwater aquifers in this MAR scheme to lower the groundwater salinity. Currently, your household may be using MAR water or other water sources for drinking and cooking purposes. Since your household is among one of the households under the MAR catchment area, we are inviting you to participate in this study.

Methods and procedures
If your household decides to participate, we make two visits to your households. The study duration will be around four months. We will collect information from all the available household members $\geq$20 years old. In each visit, we will measure the blood pressure of all the $\geq$20 years household members and may also measure their 24 hour ambulatory arterial blood pressure by strapping a device in their arms for next 24 hours. We will collect about 20 ml urine

For peer review only

from all the household members as spot urine and will provide a large bag to collect the total amount of urine during the next 24 hours time. We will measure the total protein, creatinine, in urine that will provide important information about your kidney health. In the first visit, the research staff will collect the demographic and socio-economic information, smoking and diseases history, and measure height of available household members. We will also collect 5 ml blood from the participant to assess the genetic marker to understand whether their gene indicates that reduction of salt intake can reduce blood pressure of your family members. Samples will be stored for up to 5 years after the end of the study because we want to take advantage of expected future advances in characterizing genetic markers and other parameters relevant to the salt sensitivity of hypertension. At the end of this time, the samples will be destroyed and a certificate of their destruction will be provided to iccdr,b. If you wish to withdraw your samples from the study earlier you may contact the investigators.

In addition, we will collect information about your drinking water sources during each visit and will collect your stored drinking water samples for measuring salinity and sodium concentration in water.

Risk and benefits

There is no more than minimal risk involved in this study. Your household members may feel bit discomfort during blood pressure measurement, blood and urine sample collection, but these will provide important health messages about your household member's health. Hypertension is very prevalent in Bangladesh and it remains undetected to many people. By participating in this study, your household members will have a screening for hypertension.

There will be also no social harm to you and the rest of the households in this village by your participation in this study. During the visit day, your household need to spend time (~90 minutes) from your daily life for study purposes. There is no direct monetary compensation benefit for participating in this study. However, if drinking MAR water seems to provide health benefits, the study result will help to scale MAR across coastal Bangladesh that many people will have access to safe water with low salinity.

Privacy, anonymity and confidentiality

We will maintain the confidentiality of what information you give us and we will not disclose your identity when we write our reports. We will only use the collective information for the purpose of this study, and we will not use your name in sharing and publishing the results of this study. We expect the steps we take will keep all of your information confidential.

Future use of information

The information collected from this study may be shared with other researchers if needed, but we will strictly maintain your confidentiality and privacy. In the future, we may wish to perform additional tests on the urine and blood sample that will be collected. The samples will be stored at icddr,b.

Right not to participate and withdraw

Taking part in the study is completely voluntary. You may choose not to answer any or all of the questions that will be asked about your household or your behavior. You can drop out of this

For peer review only

study at any time during the interview or anytime during the study period. You have the right to refuse participation in this study.

Principle of compensation

You need not to pay us to take part in this study, and similarly we will not pay you money for attending in the study. You will also be provided best possible, free treatment, for research related injuries.

[Note: Payment for loss of earning of the study participants may be considered in case the participants require extended hospitalization or confinement only for the purpose of the research and/or reimbursement of cost of transportation for participating in the study. However, the amount should be equivalent to the loss and not so high that the offer might induce (influence/bias judgment) participation in the study.

Answering your questions/ Contact persons

If you have any question, you can ask me any time. If you have additional questions about the survey, you may contact: Dr. Solaiman Doza ,Infectious Disease Division, icddr,b, Mohakhali, Dhaka 1212. Phone: +880-2-9827001-10, ext. 4010

If you have questions about your rights as a participant of this research study, or if you think some harm has been done to you because of the survey, you may contact the ICDDR,B IRB: M. A. Salam Khan, IRB Secretariat, phone: 9827084 or PABX 9827001-10 ext. 3206

If you agree to our proposal of enrolling you/your patient in our study, please indicate that by putting your signature or your left thumb impression at the specified space below

Thank you for your cooperation

______________________________	________________
Signature or left thumb impression	Date
______________________________	________________
Signature or left thumb impression of the witness	Date
______________________________	________________
Signature of the PI or his/her representative	Date

(NOTE: In case of representative of the PI, she/he shall put her/his full name and designation and then sign)

For peer review only

Supplemental Information 1B: Consent from ≥20 year household members

Protocol Title: Health impacts of a climate change adaptation strategy to address drinking-water salinity in coastal Bangladesh

Investigator's name: Dr. Md. Mahbubur Rahman

Organization: **International Centre for Diarrhoeal Disease Research, Bangladesh (ICDDR,B)**

Purpose of the research

Hello (Assalamualaikum/Nomoshkar). My name is ________ and I work with the ICDDR,B (Cholera Hospital) in Dhaka. You are aware that a Managed aquifer recharge scheme has already been installed in your community that would like reduce the salinity problem in your drinking water. You may also know that drinking saline water is associated with hypertension. We will conduct a pilot study to understand the mean blood pressure of the household members who are drinking water from MAR .

Background (brief introduction of the issue and the need for/ importance of the research)
Salinity in drinking water is a big problem in your community and in many other communities in the south-western coastal region of Bangladesh. Previous research has demonstrated that drinking saline water caused high blood pressure among the adult population and gestational hypertension among the pregnant women. Hypertension risk many other cardiovascular diseases and gestational hypertension also causes adverse pregnancy and fetal outcomes. Managed aquifer recharge (MAR) is a promising sustainable intervention that can reduce salinity in groundwater. Moreover, MAR will not be affected by the cyclone and tidal surges. There is a potential to scale-up MAR across the south-western coastal region if we can demonstrate that drinking MAR water provide health benefits and do not pose any risk to human health. Now we would like to conduct a pilot study where we will measure your mean blood pressure and the salt concentration of urine.

Why invited to participate in the study?
You're invited to participate in our study because your household is located in such an area where a new MAR site has been installed. Rain water and pond water is being infiltrated into the groundwater aquifers in this MAR scheme to lower the groundwater salinity. Currently, you may be using MAR water or other water sources for drinking and cooking purposes. We are inviting all the household members ≥20 years old in this MAR scheme catchment area to routinely measure blood pressure and collect urine samples. Since your age is >20 (or 20) years, your participation is very important for the study.

Methods and procedures
If you decide to participate, we will make two visits to measure your blood pressure, collect urine and blood. We may also measure your 24 hour ambulatory arterial blood pressure by strapping a device in your arm for 24 hours. We will collect about 20 ml urine as spot urine and

For peer review only

will provide a large bag to collect the total amount of urine during the 24 hours time. We will measure the total protein, creatinine, in urine that will provide important information about your kidney health. In the first visit, the research staff will collect the demographic and socio-economic information, smoking and diseases history, and measure height of available household members. We will also collect 5 ml of your blood to assess the genetic marker to understand whether your gene indicates that reduction of salt intake can reduce blood pressure of your family members.

So, if you agree, we may also invite you for some further discussions. We would like to record this sessions in a tape recorder as it is very difficult to write all the conversation in a note pad. We would also like to take some notes from this session. This session will last about an hour and half.

Risk and benefits

There is no more than minimal risk involved in this study. You may feel bit discomfort during blood pressure measurement, and urine sample collection, but these will provide important health messages about your health. Hypertension is very prevalent in Bangladesh and it remains undetected to many people. By participating in this study, you will be screened for hypertension.

There will be also no social harm to you and the rest of the households in this village by your participation in this study. During the visit day, you may need to spend time (~90 minutes) from your daily life for study purposes. There is no direct monetary compensation benefit for participating in this study. However, if drinking MAR water seems to provide health benefits, the study result will help to scale MAR across coastal Bangladesh that many people will have access to safe water with low salinity.

Privacy, anonymity and confidentiality

We will maintain the confidentiality of what information you give us and we will not disclose your identity when we write our reports. We will only use the collective information for the purpose of this study, and we will not use your name in sharing and publishing the results of this study. We expect the steps we take will keep all of your information confidential.

Future use of information

The information collected from this study may be shared with other researchers if needed, but we will strictly maintain your confidentiality and privacy. In the future, we may wish to perform additional tests on the urine sample that will be collected. The samples will be stored at icddr,b.

Right not to participate and withdraw

Taking part in the study is completely voluntary. You may choose not to answer any or all of the questions that will be asked about your household or your behavior. You can drop out of this study at any time during the interview or anytime during the study period. You have the right to refuse participation in this study.

Principle of compensation

For peer review only

You need not to pay us to take part in this study, and similarly we will not pay you money for attending in the study. You will also be provided best possible, free treatment, for research related injuries.

[Note: Payment for loss of earning of the study participants may be considered in case the participants require extended hospitalization or confinement only for the purpose of the research and/or reimbursement of cost of transportation for participating in the study. However, the amount should be equivalent to the loss and not so high that the offer might induce (influence/bias judgment) participation in the study.

Answering your questions/ Contact persons

If you have any question, you can ask me any time. If you have additional questions about the survey, you may contact: Dr. Solaiman Doza ,Infectious Disease Division, icddr,b, Mohakhali, Dhaka 1212. Phone: +880-2-9827001-10, ext. 4010

If you have questions about your rights as a participant of this research study, or if you think some harm has been done to you because of the survey, you may contact the ICDDR,B IRB: M. A. Salam Khan, IRB Secretariat, phone: 9827084 or PABX 9827001-10 ext. 3206

If you agree to our proposal of enrolling you/your patient in our study, please indicate that by putting your signature or your left thumb impression at the specified space below

Thank you for your cooperation

________________________________ ________________
Signature or left thumb impression Date

________________________________ ________________
Signature or left thumb impression of the witness Date

________________________________ ________________
Signature of the PI or his/her representative Date

(NOTE: In case of representative of the PI, she/he shall put her/his full name and designation and then sign

For peer review only

SPIRIT

STANDARD PROTOCOL ITEMS: RECOMMENDATIONS FOR INTERVENTIONAL TRIALS

SPIRIT 2013 Checklist: Recommended items to address in a clinical trial protocol and related documents*

Section/item	Item No	Description	Addressed on page number
**Administrative information**			
Title	1	Descriptive title identifying the study design, population, interventions, and, if applicable, trial acronym	Page 1
Trial registration	2a	Trial identifier and registry name. If not yet registered, name of intended registry	Page 3
	2b	All items from the World Health Organization Trial Registration Data Set	NA
Protocol version	3	Date and version identifier	MAR design paper_30 May 2017.doc
Funding	4	Sources and types of financial, material, and other support	Page 22
Roles and responsibilities	5a	Names, affiliations, and roles of protocol contributors	Page 22
	5b	Name and contact information for the trial sponsor	Page 22
	5c	Role of study sponsor and funders, if any, in study design; collection, management, analysis, and interpretation of data; writing of the report; and the decision to submit the report for publication, including whether they will have ultimate authority over any of these activities	Page 22, 18

For peer review only

	5d	Composition, roles, and responsibilities of the coordinating centre, steering committee, endpoint adjudication committee, data management team, and other individuals or groups overseeing the trial, if applicable (see Item 21a for data monitoring committee)	NA
**Introduction**			
Background and rationale	6a	Description of research question and justification for undertaking the trial, including summary of relevant studies (published and unpublished) examining benefits and harms for each intervention	Page 4 —7
	6b	Explanation for choice of comparators	Page 8
Objectives	7	Specific objectives or hypotheses	Page 7
Trial design	8	Description of trial design including type of trial (eg, parallel group, crossover, factorial, single group), allocation ratio, and framework (eg, superiority, equivalence, noninferiority, exploratory)	Page 8
**Methods: Participants, interventions, and outcomes**			
Study setting	9	Description of study settings (eg, community clinic, academic hospital) and list of countries where data will be collected. Reference to where list of study sites can be obtained	Page 8
Eligibility criteria	10	Inclusion and exclusion criteria for participants. If applicable, eligibility criteria for study centres and individuals who will perform the interventions (eg, surgeons, psychotherapists)	Page 9
Interventions	11a	Interventions for each group with sufficient detail to allow replication, including how and when they will be administered	Page 8 —10
	11b	Criteria for discontinuing or modifying allocated interventions for a given trial participant (eg, drug dose change in response to harms, participant request, or improving/worsening disease)	NA
	11c	Strategies to improve adherence to intervention protocols, and any procedures for monitoring adherence (eg, drug tablet return, laboratory tests)	Page 9, 10
	11d	Relevant concomitant care and interventions that are permitted or prohibited during the trial	NA

For peer review only - http://bmjopen.bmj.com/site/about/guidelines.xhtml

Outcomes	12	Primary, secondary, and other outcomes, including the specific measurement variable (eg, systolic blood pressure), analysis metric (eg, change from baseline, final value, time to event), method of aggregation (eg, median, proportion), and time point for each outcome. Explanation of the clinical relevance of chosen efficacy and harm outcomes is strongly recommended	Page 13 — 15
Participant timeline	13	Time schedule of enrolment, interventions (including any run-ins and washouts), assessments, and visits for participants. A schematic diagram is highly recommended (see Figure)	Figure 3
Sample size	14	Estimated number of participants needed to achieve study objectives and how it was determined, including clinical and statistical assumptions supporting any sample size calculations	Page 10,11
Recruitment	15	Strategies for achieving adequate participant enrolment to reach target sample size	Page 9, 10
**Methods: Assignment of interventions (for controlled trials)**			
Allocation:			
Sequence generation	16a	Method of generating the allocation sequence (eg, computer-generated random numbers), and list of any factors for stratification. To reduce predictability of a random sequence, details of any planned restriction (eg, blocking) should be provided in a separate document that is unavailable to those who enrol participants or assign interventions	Page 9
Allocation concealment mechanism	16b	Mechanism of implementing the allocation sequence (eg, central telephone; sequentially numbered, opaque, sealed envelopes), describing any steps to conceal the sequence until interventions are assigned	NA
Implementation	16c	Who will generate the allocation sequence, who will enrol participants, and who will assign participants to interventions	Page 9
Blinding (masking)	17a	Who will be blinded after assignment to interventions (eg, trial participants, care providers, outcome assessors, data analysts), and how	NA
	17b	If blinded, circumstances under which unblinding is permissible, and procedure for revealing a participant's allocated intervention during the trial	NA
**Methods: Data collection, management, and analysis**			

Data collection methods	18a	Plans for assessment and collection of outcome, baseline, and other trial data, including any related processes to promote data quality (eg, duplicate measurements, training of assessors) and a description of study instruments (eg, questionnaires, laboratory tests) along with their reliability and validity, if known. Reference to where data collection forms can be found, if not in the protocol	Page 12 — 16
	18b	Plans to promote participant retention and complete follow-up, including list of any outcome data to be collected for participants who discontinue or deviate from intervention protocols	Page 9,10
Data management	19	Plans for data entry, coding, security, and storage, including any related processes to promote data quality (eg, double data entry; range checks for data values). Reference to where details of data management procedures can be found, if not in the protocol	Page 16
Statistical methods	20a	Statistical methods for analysing primary and secondary outcomes. Reference to where other details of the statistical analysis plan can be found, if not in the protocol	Page 16, 17
	20b	Methods for any additional analyses (eg, subgroup and adjusted analyses)	Page 16, 17
	20c	Definition of analysis population relating to protocol non-adherence (eg, as randomised analysis), and any statistical methods to handle missing data (eg, multiple imputation)	Page 16, 17
**Methods: Monitoring**			
Data monitoring	21a	Composition of data monitoring committee (DMC); summary of its role and reporting structure; statement of whether it is independent from the sponsor and competing interests; and reference to where further details about its charter can be found, if not in the protocol. Alternatively, an explanation of why a DMC is not needed	Page 16
	21b	Description of any interim analyses and stopping guidelines, including who will have access to these interim results and make the final decision to terminate the trial	NA
Harms	22	Plans for collecting, assessing, reporting, and managing solicited and spontaneously reported adverse events and other unintended effects of trial interventions or trial conduct	Page 16
Auditing	23	Frequency and procedures for auditing trial conduct, if any, and whether the process will be independent from investigators and the sponsor	NA
**Ethics and dissemination**			

For peer review only - http://bmjopen.bmj.com/site/about/guidelines.xhtml

Research ethics approval	24	Plans for seeking research ethics committee/institutional review board (REC/IRB) approval	Page 17
Protocol amendments	25	Plans for communicating important protocol modifications (eg, changes to eligibility criteria, outcomes, analyses) to relevant parties (eg, investigators, REC/IRBs, trial participants, trial registries, journals, regulators)	Page 17
Consent or assent	26a	Who will obtain informed consent or assent from potential trial participants or authorised surrogates, and how (see Item 32)	Page 9, 12, 17
	26b	Additional consent provisions for collection and use of participant data and biological specimens in ancillary studies, if applicable	Page 17
Confidentiality	27	How personal information about potential and enrolled participants will be collected, shared, and maintained in order to protect confidentiality before, during, and after the trial	Page 17
Declaration of interests	28	Financial and other competing interests for principal investigators for the overall trial and each study site	Page 22
Access to data	29	Statement of who will have access to the final trial dataset, and disclosure of contractual agreements that limit such access for investigators	Page 22
Ancillary and post-trial care	30	Provisions, if any, for ancillary and post-trial care, and for compensation to those who suffer harm from trial participation	NA
Dissemination policy	31a	Plans for investigators and sponsor to communicate trial results to participants, healthcare professionals, the public, and other relevant groups (eg, via publication, reporting in results databases, or other data sharing arrangements), including any publication restrictions	Page 18
	31b	Authorship eligibility guidelines and any intended use of professional writers	
	31c	Plans, if any, for granting public access to the full protocol, participant-level dataset, and statistical code	Page 22
**Appendices**			
Informed consent materials	32	Model consent form and other related documentation given to participants and authorised surrogates	Appendix 1

For peer review only - http://bmjopen.bmj.com/site/about/guidelines.xhtml

For peer review only

Biological specimens	33	Plans for collection, laboratory evaluation, and storage of biological specimens for genetic or molecular analysis in the current trial and for future use in ancillary studies, if applicable	NA

*It is strongly recommended that this checklist be read in conjunction with the SPIRIT 2013 Explanation & Elaboration for important clarification on the items. Amendments to the protocol should be tracked and dated. The SPIRIT checklist is copyrighted by the SPIRIT Group under the Creative Commons "Attribution-NonCommercial-NoDerivs 3.0 Unported" license.

For peer review only

For peer review only - http://bmjopen.bmj.com/site/about/guidelines.xhtml

BMJ Open

Stepped-wedge cluster-randomized controlled trial to assess the cardiovascular health effects of a managed aquifer recharge initiative to reduce drinking water salinity in southwest coastal Bangladesh: study design and rationale

For peer review only

Journal:	*BMJ Open*
Manuscript ID	bmjopen-2016-015205.R2
Article Type:	Protocol
Date Submitted by the Author:	16-Jun-2017

Complete List of Authors:	Naser, Abu; Emory University, Department of Environmental Health Sciences, Rollins School of Public Health<br>Unicomb, Leanne; International Centre for Diarrhoeal Disease Research, Centre for Communicable Diseases<br>Doza, Solaiman; International Centre for Diarrhoeal Disease Research, Centre for Communicable Diseases<br>Ahmed, Kazi; Dhaka University, Department of Geology<br>Rahman, Mahbubur ; International Centre for Diarrhoeal Disease Research, Centre for Communicable Diseases<br>Uddin, Mohammad; International Centre for Diarrhoeal Disease Research, Centre for Communicable Diseases<br>Quraishi, Shamshad; Atomic Energy Centre, Analytical Chemistry Laboratory<br>Selim, Shahjada; Bangabandhu Sheikh Mujib Medical University<br>Shamsudduha, Mohammad ; University College London, Institute for Risk and Disaster Reduction<br>Burgess, William; University College London, Department of Earth Sciences<br>Chang, Howard; Emory University, Department of Biostatistics and Bioinformatics, Rollins School of Public Health, Emory University<br>Gribble, Matthew; Emory University, Department of Environmental Health Sciences, Rollins School of Public Health<br>Clasen, Thomas; Emory University, Department of Environmental Health Sciences, Rollins School of Public Health<br>Luby, Stephen; International Centre for Diarrhoeal Disease Research, Centre for Communicable Diseases; Stanford University, Woods Institute for the Environment

**Primary Subject Heading**:	Public health
Secondary Subject Heading:	Epidemiology, Global health, Occupational and environmental medicine, Cardiovascular medicine
Keywords:	stepped-wedge trial, Managed aquifer recharge, Blood pressure, Sodium intake, Drinking saline water, Coastal aquifer

For peer review only

Stepped-wedge cluster-randomized controlled trial to assess the cardiovascular health effects of a managed aquifer recharge initiative to reduce drinking water salinity in southwest coastal Bangladesh: study design and rationale

Abu Mohd Naser[1*], Leanne Unicomb[2], Solaiman Doza[2], Kazi Matin Ahmed[3], Mahbubur Rahman[2], Mohammad Nasir Uddin[2], Shamshad B. Quraishi[4], Shahjada Selim[5], Mohammad Shamsudduha[6], William Burgess[7], Howard H. Chang[8], Matthew O. Gribble[1], Thomas F. Clasen[1], Stephen P. Luby[9]

[1] Department of Environmental Health Sciences, Rollins School of Public Health, Emory University, Atlanta, USA

[2]International Centre for Diarrhoeal Disease Research, Bangladesh (icddr,b), GPO Box 128, Dhaka 1000, Bangladesh

[3] Department of Geology, University of Dhaka, Dhaka, Bangladesh

[4]Analytical Chemistry Laboratory, Atomic Energy Centre, Dhaka, Bangladesh

[5]Bangabandhu Sheikh Mujib Medical University, Dhaka, Bangladesh

[6] Institute for Risk and Disaster Reduction, University College London, UK

[7] Department of Earth Sciences, University College London, London, UK

[8] Department of Biostatistics and Bioinformatics, Rollins School of Public Health, Emory University, Atlanta, USA

[9]Woods Institute for the Environment, Stanford University, Stanford, California, USA

*Corresponding author:

Abu Mohd Naser, MBBS, MPH
Department of Environmental Health Sciences
Rollins School of Public Health | Emory University
2nd floor, CNR building, Room # 2030E
Atlanta, GA 30322, USA
email: atitu@emory.edu

Abstract

Introduction: Saltwater intrusion and salinization have contributed to drinking water scarcity in many coastal regions globally, leading to dependence on alternative sources for water supply. In southwest coastal Bangladesh, communities have few options but to drink brackish groundwater which has been associated with high blood pressure among the adult population, and preeclampsia and gestational hypertension among pregnant women. Managed aquifer recharge (MAR), the purposeful recharge of surface water or rainwater to aquifers to bring hydrologic equilibrium, is a potential solution for salinity problem in southwest coastal Bangladesh by creating a freshwater lens within the brackish aquifer. Our study aims to evaluate whether consumption of MAR water improves human health, particularly by reducing blood pressure among communities in coastal Bangladesh.

Methods and analysis: The study employs a stepped-wedge cluster-randomized controlled community trial design in 16 communities over five monthly visits. During each visit, we will collect data on participants' source of drinking and cooking water and measure the salinity level and electrical conductivity of household stored water. At each visit, we will also measure the blood pressure of participants' ≥ 20 years of age and pregnant women and collect urine samples for urinary sodium and protein measurements. We will use generalized linear mixed models to determine the association of access to MAR water on blood pressure of the participants.

Ethics and dissemination: The study protocol has been reviewed and approved by the Institutional Review Boards of the International Centre for Diarrheal Disease Research, Bangladesh (icddr.b). Informed written consent will be taken from all the participants. This study is funded by Wellcome Trust, UK. The study findings will be disseminated to the government partners, at research conferences, and in peer-reviewed journals.

For peer review only - http://bmjopen.bmj.com/site/about/guidelines.xhtml

Registration: (http://www.clinicaltrials.gov): NCT02746003

Strengths and limitation of the study

- This is the first study to evaluate the health impact of managed aquifer recharge in southwest coastal Bangladesh.
- The stepped-wedge trial ensures we will have counterfactual data as well as gradual access to MAR water in all communities.
- Objective measurement of exposure (drinking water salinity) and outcomes (urinary sodium and blood pressure).
- The magnitude of exposure will vary geographically and across time period. Therefore, MAR water salinity will differ across communities at a single point of time, and also for the same community at different points of time.
- Compliance of the intervention may be different across sites and for individuals of different socioeconomic status.

For peer review only

For peer review only - http://bmjopen.bmj.com/site/about/guidelines.xhtml

Introduction

Background and rationale: Saltwater intrusion and salinization have increased groundwater salinity in many coastal aquifers and small islands across the world.[1-5] This is driven by a number of climatological and anthropogenic factors including global warming, increased cyclones and tidal surges, reduced river discharge, and increased groundwater abstraction in excess of recharge.[6-8] Communities in many coastal regions rely on groundwater as their main source of drinking water [9] as well as freshwater for domestic, agricultural and industrial purposes.[10] Nearly half of the world's population resides in coastal areas [3] and 10% of these live in low-lying coastal areas where surface elevation is generally ≤10 meters above mean sea level.[11] Salinization in coastal areas is expected to increase in the future because of increased groundwater withdrawal due to population and economic growth and sea-level rise.[3] As the world's population and economic activities continue to grow, groundwater supplies are progressively under threat of depletion, which increases the importance of monitoring, management and conservation of coastal freshwater aquifers.[12 13]

One approach to minimize the impact of groundwater salinization is to enhance groundwater recharge into coastal aquifers.[14] Managed aquifer recharge (MAR) is an approach to artificially promote freshwater recharge to increase storage. MAR involves infiltration of freshwater (e.g. rainwater and pond water) into aquifers to create a store of freshwater within the naturally brackish aquifer (**Figure 1**).[15-17] MAR represents a promising adaptive strategy for increasing freshwater availability and sustaining a year-round drinking water supply that is protected from evaporation, and could be resilient to tidal storms, cyclones and surface water salinity since freshwater infiltration and storage occur under confined conditions.[18]

For peer review only - http://bmjopen.bmj.com/site/about/guidelines.xhtml

The brackishness of groundwater in southwest coastal Bangladesh is caused by a combination of climatic and anthropogenic factors including sea level rise, frequent cyclones and tidal storms, and shrimp cultivation.[19-22] In the future, climate change and sea-level rise are expected to cause more cyclones, tidal surges and flood in this region that will further affect surface water and groundwater salinity.[7] In many areas in southwest coastal Bangladesh, both shallow and deep aquifers contain naturally brackish water causing acute scarcity of drinking water (**Figure 2A**).[23 24] Water salinity in southwest coastal Bangladesh follows a clear seasonal pattern ─ higher in the dry season than the wet season.[25] Salinity of water bodies builds up from October to May, peaking during February to early May. After May, salinity decreases sharply due to rainfall and increased upstream river flow. People collect and use rainwater for drinking and cooking purpose during the monsoon when precipitation is intense, but during the dry season, they generally rely on pond water or saline tube well water.[26]

The Geology Department of the University of Dhaka, in collaboration with UNICEF and the Department of Public Health Engineering (DPHE), Government of Bangladesh have piloted 20 small-scale MAR projects in three districts of southwest coastal Bangladesh to evaluate the feasibility of MAR for drinking water supply in rural communities.[27] The shallow brackish aquifer was the target storage zone that is overlain by Holocene clay aquitard of 3 to 15 meter thickness.[16] In the pilot phase, an average storage of approximately 900 m^3 of fresh water per year per site was established, sufficient to deliver 15 L of safe drinking water per day per household, which can fulfill the demand for drinking and cooking for approximately 300 people in 60-70 households during the dry season.[28 29] The second phase of the MAR project started in 2014 aiming to install 75 MAR systems in three districts of southwest coastal Bangladesh: Satkhira, Khulna and Bagerhat (**Figure 2B**). In March, 2016, 30 MAR sites were handed over to

For peer review only - http://bmjopen.bmj.com/site/about/guidelines.xhtml

the community for water consumption. The remaining 45 sites are expected to be ready by November 2016 following infiltration of water during monsoon of 2016 (June – October, 2016).

Epidemiological studies have demonstrated that high sodium intake is associated with elevated blood pressure [30-32] and other cardiovascular diseases.[33] A study conducted among the adult population residing in southwest coastal Bangladesh suggest drinking saline water was associated with high blood pressure after adjusting for personal, lifestyle and environmental factors.[34] Among study participants, the mean systolic blood pressure for those consuming water with sodium from the lowest quintile was 119.4 (SD 13.7) and from the highest sodium quintile was 126.7 mm Hg (18·0).[34] Excess sodium intake from drinking brackish water has been also associated increased gestational hypertension and preeclampsia among pregnant women in southwest coastal Bangladesh.[35 36] Mean systolic and diastolic blood pressure were higher among the pregnant women who drank tube well or pond (saline sources) water compared to those who drank rain water.[35 37] The mean systolic blood pressure of pregnant women from these areas was 102.4 mm Hg among those who drank rainwater, 112.6 mm Hg among pond water users and 119.4 mm Hg among brackish groundwater users.[35] High blood pressure during pregnancy is associated with high maternal mortality, and adverse pregnancy and fetal outcomes.[38]

There are 37 million people living in the southwest coastal region and 20 million are currently affected by drinking water salinity.[39] The estimated mean global sodium consumption is 3.95 g per day (range 2.2 to 5.5 g per day),[40] but people in southwest coastal Bangladesh consume up to 16 g of sodium per day through drinking brackish groundwater.[41] While MAR water can potentially reduce exposure to saline water, the health effects of providing access to MAR water have not been investigated. Characterizing the health consequences of shifting from

For peer review only

current drinking water supply to MAR water can assist decision-makers to assess the value of scaling up MAR. Many water interventions, for example, failed to achieve health impact, because people do not use them. There is also considerable controversy on whether reducing sodium intake improves health.[42] In addition the MAR system will also alter the intake of other cations such as calcium and magnesium that may have health impact.[43-46] Because the cost of these systems is high and their long-term feasibility an open question, clarifying the amount of health benefit, if any, is an important step that can help inform future efforts to provide water to communities faced with high levels of groundwater salinity. The human subjects committees who reviewed the study appreciated the uncertainty in bringing solutions to scale in this setting and considered it appropriate to include health measurements of participating residents to objectively evaluate the potential impact.

Objectives: Randomized controlled trials demonstrate that reduction in dietary sodium in adults decreases blood pressure among people both with and without hypertension. [47-49] Meta-analysis of randomized trials also suggest modest reduction in salt intake for four or more weeks causes significant reduction in blood pressure at the population-level. [50 51] The primary objective of the study is to assess whether access to low-salinity MAR water can reduce blood pressure of community members ≥20 years of age. Secondary objectives include whether access to MAR water can reduces urinary sodium and total protein excretion. We will evaluate water salinity, urinary sodium excretion and blood pressure so that we understand whether or not we achieved our immediate targets along the causal pathway.

For peer review only - http://bmjopen.bmj.com/site/about/guidelines.xhtml

Methods

Study design: We will implement a stepped-wedge cluster randomized controlled trial in 16 MAR communities. MAR system is a community based intervention designed to supply low-salinity water for 60-70 households in a village or community. MAR intervention cannot be implemented and randomized at individual- or household-levels. Once a community will have access to MAR water, it is difficult to withhold the access of MAR water for some households. Therefore, we will conduct a cluster randomized trial where each community will be considered as a cluster. The stepped wedge design allows communities to gradually have access to MAR water; however, the point at which their access commences will be randomly assigned.[52] In this way, each MAR site will contribute data for both the intervention and the control time periods. We will have five monthly steps in the stepped wedge trial (**Figure 3**). In the first step, none of the communities will have MAR water available for drinking and we will collect baseline information. During each subsequent month, four randomly selected communities will receive access to MAR water for drinking and cooking. In the last (fifth) monthly step, all the communities will have access to MAR drinking water.

Study settings: The study will be conducted at the community-level in three districts in southwest coastal Bangladesh ─ Khulna, Satkhira and Bagerhat ─ where 75 MAR systems have been installed. Of these, 30 systems are already in use. We will select 16 communities for study based on consultation with the implementers of MAR systems (Dhaka University and UNICEF, Bangladesh). Three criteria need to be met for inclusion of communities in the study: communities have not started drinking MAR water, acceptable level of arsenic (<50 μg/L) in drinking water set by the government of Bangladesh,[53 54] and electrical conductivity (a measure

For peer review only - http://bmjopen.bmj.com/site/about/guidelines.xhtml

of salinity) of water below 2000 µS/cm, an indication that the MAR system has successfully reduced salinity in the aquifer.

Participant eligibility criteria: The inclusion criteria will be households whose ≥ 20 year old members and pregnant women willingly agree to exclusively use MAR water during the dry season for drinking and cooking purposes. The Dhaka University team have determined the catchment area for each MAR site based on geographic distance from MAR infrastructure and have developed a list of households who expressed willingness to drink MAR water. Any ≥ 20 year old hypertensive household members will be also eligible for participation but research staff will collect information about their medication if any. Research staff will enrol post-adolescent ≥ 20 year population as an individual's response to salt intake (salt-sensitivity) increases with age[55] and adolescence is associated adrenal and nervous system maturation that may contribute to salt sensitivity.[56 57] They will approach households living near the MAR water access point. They will enroll the 28 closest households surrounding each MAR systems that meet the inclusion criteria and consent (Supplemental Information 1) to study participation. During the informed written consent process, research staff will inform each household that they will be randomly selected when to start consuming MAR water as part of the scientific process of the study. All household members ≥20 year of age will be eligible and enrolled in the study from the selected 28 households. In addition, research staff will enroll households that include a pregnant woman in each selected MAR catchment community irrespective of household selection, from a list of pregnant women developed by a female promoter.

Randomization and intervention delivery: We will randomly select four MAR communities to drink MAR water in each step following the first (baseline) step (**Figure 3**) by computer-generated random numbers. Randomization will be conducted by an investigator who

For peer review only - http://bmjopen.bmj.com/site/about/guidelines.xhtml

will not be directly involved in implementation of the stepped wedge study and this will be done before commencement of the study. The study could not be blinded therefore there was no concealment from the cluster participants. Although infiltration of rainwater and pond water into the brackish aquifer through the MAR systems will be ongoing for 1-2 years in each community, people will not have access to MAR water until a formal handover of MAR systems to community members. The implementers hire a caretaker or gatekeeper for each MAR community who is responsible for maintenance of the respective MAR system prior to the community hand over. The implementers form a community management team who are responsible for maintenance of each MAR system following handover. We have synchronized the community handover with the randomization schedule in the 16 communities after consulting with the gatekeepers. Community health promoters will inform the participants about when the MAR water will be available for consumption once the community management team is formed. Agreement of the implementers and gatekeepers was sought at the beginning of the study during site selection for inclusion of any site in trial and access of MAR water as per the randomization.

We will deploy a local trained promoter at each MAR site, who will visit these households, list members and identify pregnant women in these and other households in catchment areas of each MAR site. Promoters will encourage all household members to drink MAR water exclusively while they are at home, carry a bottle of MAR water while they go out for work and other activities, and to cook with MAR water. The community health promoters will visit households with promotional materials (e.g. flip charts) from the beginning of the study to inform household members about adverse health effects of drinking brackish water and potential benefits of drinking low-salinity MAR water. As per the randomization schedule, they will inform households when MAR water will be available for consumption.

For peer review only - http://bmjopen.bmj.com/site/about/guidelines.xhtml

Sample size: The sample size of the stepped wedge trial was calculated based on the effect of MAR systems to reduce systolic blood pressure of the ≥ 20 years old community members. Data from southwest coastal Bangladesh demonstrated a 7 mmHg lower mean systolic blood pressure among pregnant women who drank pond water compared to those who drank brackish water, and a 17 mmHg lower mean systolic blood pressure for pregnant women who drank rainwater compared to those who drank brackish water [35]. The mean reduction in systolic blood pressure following long term modest salt reduction is 4.2 mmHg.[50] For calculations we considered a mean systolic blood pressure reduction of at least 3 mmHg among the communities who will have access to MAR water for drinking and cooking purposes compared to those who will use brackish water. The entire population under the MAR community catchment area will be considered as one cluster. Each MAR community serves approximately 300 people, constituting 60-70 households in the catchment area.[28] The mean household size in southwest coastal Bangladesh is 4.2, approximately 52% of household members are ≥ 20 years.[58 59] Since we will collect blood pressure for all household members ≥ 20 years, we estimated an average of 2.2 participants in this age group per household. We will select 28 households per cluster (28 x 2.2 = 60 people per cluster). A previously published study in Bangladesh reported standard deviation (SD) for systolic blood pressure as 13.51,[60] but we considered standard deviation of systolic blood pressure 20 to capture a greater variation of blood pressure. We calculated the sample size for the stepped wedge trial adopting the formula used by Hussey and Hughes 2007.[61] Although their formula assumed no within participant correlation over time (cross sectional design), we applied their formula for a cohort design (same participant followed up over time). The salinity problem and the MAR water quality may be influenced by seasonality over the progression of dry season and participants' blood pressure may respond differently to different level of sodium

For peer review only - http://bmjopen.bmj.com/site/about/guidelines.xhtml

exposure in MAR drinking water. Therefore, it is important that we investigate the effect of the MAR system on the same participants over the entire dry season. Since we will investigate the same participants in each steps, we will have less participant-level variability and sufficient power compared to a cross-sectional design to investigate the effect of MAR systems. We initially calculated the sample size for a simple unadjusted trial (N_U), and then multiplied by the design effect for a stepped wedge trial (DE_{SW}). The sample size for a simple unadjusted trial was calculated as 1,396 participants (effect size= 3, standard deviation of 20 for both groups, 80% power, and 5% Type I error). The DE_{SW} was 3.04 based on a formula provided by Hussy and Hughes therefore sample size was 1396 x 3.04. We also inflated the total sample size considering 10% loss to follow-up. We then followed the approaches of determining the number of clusters required for total sample size considering a fixed cluster size of 60.[62] We then calculated the number of steps that we need to randomize by dividing total cluster by steps. We calculated that 16 MAR communities will be required for the study with four communities randomized to access MAR water in each step (**Figure 3**).

Data collection methods: The outcomes of interest will be measured from selected participants of 16 communities irrespective of their access to MAR water during five monthly visits. The interval between two successive blood pressure measurements or two consecutive data collection visits will be at least four weeks for each participant. After taking informed written consent from household heads and each participant, research staff will administer a survey using a structured questionnaire to collect information on drinking water sources, household members' socio-demographic status, medical history, family history of cardiovascular diseases, medication intake and cardiovascular risk factors including smoking history and alcohol intake during the first monthly visit. Exposure and outcome data will be collected in all

For peer review only - http://bmjopen.bmj.com/site/about/guidelines.xhtml

five visits. Participants' weight and hip circumference will be measured in all visits but height will be measure in one visit.

As part of the MAR uptake evaluation, the research staff will collect self-reported data on MAR water use for each households and whether households exclusively or intermittently use MAR water for drinking and cooking during follow-up visits. Research staff will also collect stored drinking and cooking water samples for measurement of electrical conductivity and will ask respondents the source of stored water.

Exposure assessment: During each visit, research staff will inquire about household members' reported primary water sources used for drinking and cooking purposes in last 24 hours, collect reported information on whether participants exclusively used the primary water sources and explore whether any alternative water sources were used. They will also ask about the frequency of collection and cost of primary water sources for drinking and cooking, time required to collect water, amount of collected water and when the last water was collected. Research staff will observe the presence of stored water in households, ask the sources of each container of stored water and will collect water samples that have been stored for drinking and cooking (if any) to measure the salinity level, electrical conductivity, resistivity, total dissolve solutes (TDS) and temperature of water samples using Hanna Salinity™ meter. They will also collect the MAR water from the source MAR outlet to measure the salinity level, electrical conductivity, resistivity, total dissolve solutes (TDS) and temperature of water. Research staff will collect 24 hour urine from each participant to measure the urinary sodium excretion as a proxy for sodium intake.[63 64]

For peer review only - http://bmjopen.bmj.com/site/about/guidelines.xhtml

Outcomes assessment: Systolic blood pressure of the ≥ 20 year old participants is the primary outcome of this study. Secondary outcomes will be diastolic blood pressure, mean arterial pressure, and pulse pressure (**Table 1**). The different components of blood pressure are independent cardiovascular risk factors associated with sodium intake,[65-69] and indicate future risk for different cardiovascular diseases.[70] High systolic blood pressure puts more stress on the vascular wall and is strongly associated with risk for future intracerebral haemorrhage, subarachnoid haemorrhage, angina, myocardial infarction and peripheral vascular diseases.[70] Raised diastolic blood pressure is associated with aortic and thoracic aneurysm.[70] Some effects of high sodium intake are independent of high systolic and diastolic blood pressure such as arterial stiffness and left ventricular mass ─ both of these are independent predictor of future cardiovascular diseases. High pulse pressure (difference between systolic and diastolic blood pressure) is associated with increased arterial stiffness,[66 68] and high mean arterial pressure (diastolic blood pressure plus one-third of systolic blood pressure) and high pulse pressure is independently associated with increased left ventricular mass.[66 68]

We will also measure the creatinine adjusted protein excretion of all participants as tertiary outcomes (**Table 1**). Proteinuria is a biomarker for future risk of cardiovascular diseases,[71-74] and is associated with the pathogenesis of cardiovascular diseases, including hypertension,[75 76] chronic kidney disease,[77] myocardial ischemia,[78] carotid artery thickness,[79 80] left ventricular hypertrophy,[81 82] hyperlipidemia,[83] atherosclerosis,[84] and coronary artery calcification.[72 80 85 86] Reduced salt intake for four weeks has been associated with decreased proteinuria in blinded randomized controlled trial.[87] Preeclampsia is associated with high maternal mortality and adverse pregnancy and fetal outcomes [38] and is characterized by high blood pressure and proteinuria after 20 weeks of pregnancy.[88]

For peer review only - http://bmjopen.bmj.com/site/about/guidelines.xhtml

For peer review only

Blood pressure measurement: Blood pressure will be measured for all ≥20 year old participants and pregnant women during each step. Research staff will use a Omron® HEM–907 blood pressure device, which is comparable to the gold standard mercury sphygmomanometers in terms of measurement and meets the Association for the Advancement of Medical Instrumentation's (AAMI) criteria.[89] Blood pressure will be measured following the guidelines described by Pickering et al. 2005 and Giorgini et al. 2014.[90 91] Caffeine (tea, coffee, carbonated beverages), eating, heavy physical activities and smoking will be proscribed for 30 minutes prior to measuring blood pressure. Participants will rest for 5 minutes sitting on a chair keeping their arm supported. An appropriate sized cuff and calibrated instrument will be used for different age groups and the blood pressure instrument will be positioned at heart level. Blood pressure will be measured three times; first left arm, then right arm, then again left arm. Both systolic and diastolic blood pressure will be recorded from all measurements. The arithmetic mean of three systolic blood pressure measurements will be used as the primary outcome. However, if a systolic blood pressure measurement differs by 10% from the other measurements, that measurement will be excluded when calculating the arithmetic mean systolic blood pressure.

Biomarker measurements: Field research staff will instruct the participants' to collect 24 hours urine sample during each household visit. The volume of the urine samples will be noted at household level, and a sample of 25 ml urine will be collected and transported to the field laboratory at 2-8° C within 6 hours of collection for processing, analysis and storage. Aliquots of each participant's urine sample will be made for biochemical and electrolyte measurements. Urinary creatinine concentration will be measured by a colorimetric method (Jaffe reaction) using a semi-auto biochemistry analyzer (Evolution 3000, BSI, Italy). Urinary total protein will be estimated using a light sensitive colored reagent (Randox.UK). We will use

For peer review only

For peer review only - http://bmjopen.bmj.com/site/about/guidelines.xhtml

the direct Ion Selective Electrode (ISE) method for urinary sodium measurements using a semi-auto electrolyte analyzer (Biolyte2000, Bio-care Corporation, Taiwan). We will measure the uric acid concentration in blood by an enzymatic colorimetric method (Evolution 3000, BSI, Italy). We will perform routine Quality Control for all tests using standard quality control reagents (Bio-Rad Laboratories, USA) and 5% of samples will be cross checked at the International Centre for Diarrhoeal Disease Research, Bangladesh (icddr,b) central laboratory, in Dhaka.

Data management and monitoring: The data collection instrument will be programmed and field research staff will use hand held computers to collect data. Appropriate range values and data values will be programmed to minimize data entry errors. The dataset downloaded from handheld devices will be cleaned and checked by the site investigators. All laboratory data will be double entered. Data will be stored in icddr,b's data repository system, in compliance with the system's requirements and will be publicly available after analyzing the primary result.

The research staff will be trained for identifying adverse events such as hypertension and hypertensive disorders in pregnancy. They will report to the investigators following identification of these patients and the investigators will assess whether these adverse events need to be reported to icddr,b's Ethical Review Committee.

Statistical methods: We will conduct an intention-to-treat analysis for the primary analysis. For the primary analysis we will assess whether access to MAR water reduce the systolic blood pressure (continuous outcome) of the ≥20 years old participants. Pregnant women will be included in the primary analysis because it is likely few pregnant women will be identified in the 16 communities and separate analysis of pregnant women will be underpowered. We will use generalized linear mixed models with appropriate links for the primary analysis considering random effects for community, households and participants, and a fixed effects of

For peer review only - http://bmjopen.bmj.com/site/about/guidelines.xhtml

steps or visits. We will adjust the effect of MAR systems on blood pressure for age, sex, weight and height, personal, and socioeconomic factors **(Table 2).** We will inspect the missing data patterns and use multiple imputation with chained equations to jointly impute data on missing exposure and confounders to preserve an unbiased association estimate if the data are missing at random conditional on measured variables. We will also conduct subgroup analyses among the households that adhere with the MAR intervention and that exclusively use MAR water. If the trial demonstrates drinking MAR water has health effects, we will investigate the time required for health effects following receiving MAR intervention in secondary analysis.

Ethics: Informed written consent will be taken from all participants and household heads. Consent will be also taken for ancillary studies and future use of specimens collected from study participants. This study protocol has been reviewed and approved by the icddr,b Ethical Review Committee. Approval will be taken for any addition or modification of the protocol from icddr,b Ethical Review Committee. If research staff members identify hypertensive patients or cases of hypertension during pregnancy, they will refer patients to the local government health facilities for further management. In addition, research staff will train pregnant women and family members to recognize the danger signs during pregnancy, and will also instruct them where to seek medical care if such danger signs appear. During monthly household visits research staff will encourage pregnant women to attend prenatal visits. All dataset will be anonymous without the personal identifiers and participants' privacy will be maintained during data storage, analysis and dissemination.

Dissemination: Study findings will be shared with the Department of Public Health Engineering (DPHE), Department of Environment of the government of Bangladesh and other partner NGOs working in southwest coastal Bangladesh for access to safe drinking water. We

For peer review only

For peer review only - http://bmjopen.bmj.com/site/about/guidelines.xhtml

will discuss the scope and limitations of the MAR system to address the demand of safe drinking water based on findings from this study. We will be submitting abstracts to international conferences for dissemination to the international audience working on safe water. We will develop manuscripts and submit to peer-reviewed journals to publish our research.

For peer review only

For peer review only - http://bmjopen.bmj.com/site/about/guidelines.xhtml

Discussion

This will be the first study to assess the health impact of an environmental intervention to reduce groundwater salinity in southwest coastal Bangladesh. Our study has several strengths. The stepped-wedge trial ensures that we will have counterfactual data as well as gradual access to MAR water in all communities. In a stepped-wedge design, treatment effect of an intervention can be estimated from between- and within-cluster comparisons as participants will act as their own control, compared to only between-cluster comparison in a parallel cluster randomized design.[61]

We explored other study designs such as non-randomized study. As per suggestion of icddr,b's Ethical Review Committee, we conducted a pilot study in four communities (two MAR communities and two non-MAR communities) during May-August 2016. Pilot study findings suggested that because of the heterogeneity in village level hydrogeological conditions and in participation by community residents with different characteristics, non-randomized quasi-experimental studies would expected to generate unequal distribution of confounders that would undermine scientific inference. The objective of the pilot study was to pre-test our data collection instruments laboratory protocols prior commencement of the main study We identified unequal distributions of several important observed confounders across the communities such as socio-economic status, and household characteristics such as supply of electricity and ownership refrigerator. We identified that some covariates such as supply of electricity was perfectly predictive of communities receiving MAR interventions. We were unable to derive satisfactory matching of MAR intervention and control households after principal component analysis. The findings highlighted the importance of a randomized trial to evaluate the health effects of MAR

For peer review only - http://bmjopen.bmj.com/site/about/guidelines.xhtml

systems since there are many observed and unobserved household and community-level and hydrogeological confounders that may influence blood pressure.

We also explored the possibilities of parallel group randomized trial but several programmatic issues barred us to implement a parallel group cluster randomized trial. The implementers of MAR systems (UNICEF Bangladesh and University of Dhaka) installed the 75 new sites in different stages and planned to allow communities to access these over three dry seasons. The implementers informed us that 20-25 MAR sites may be suitable for community access during our study period and they wanted to allow all communities to drink MAR water in the same season after satisfactory reduction in salinity. Therefore, a parallel group cluster randomized trial was not suitable as we could not randomly select control sites and postpone the community to drink water for the dry season. We believe that the stepped-wedge trial design was appropriate to obtain counterfactual data as well as fulfilling the programmatic need.

We will use the same instruments for data collection and outcome measurements for all steps for exposed and unexposed communities, which will mitigate bias in data collection. Measurement of water source salinity and urinary sodium concentration will explain the biologically plausible effect of drinking MAR water on blood pressure. All outcomes planned to measure in the study are objective outcomes that will reduce the risk of reporting bias. Collection of detailed exposure and co-variate data will help in determining a valid association between drinking MAR water and health benefits. We will have several outcome variables and biomarkers that will ensure a comprehensive health benefit evaluation of access to MAR water.

The study has several limitations. We will be unable to control salinity level of drinking water across the 16 MAR communities due to different hydro-geological conditions and volume

For peer review only - http://bmjopen.bmj.com/site/about/guidelines.xhtml

of infiltrated water across the aquifers. Although the initial electrical conductivity of MAR water will be < 2000 μS/cm, electrical conductivity is likely to vary across different MAR communities with the intensification of dry season. Therefore, MAR water salinity will differ across communities at a single point of time, and also for the same community at different points of time. This will result in different versions of exposures (i.e. access to MAR water with different salinity-levels) that may affect different responses for some participants depending on the version of exposure they will receive.[92 93] To account this, we will specify the versions of exposure by measuring the salinity level of MAR water available in participants' households and interpret the response as unit change of salinity at different MAR water salinity-levels. As compliance of drinking MAR water may be different across communities despite active encouragement by promoters, one problem of the intention to treat analysis is that if the proportions of participants who will always drink MAR water is low compared to those who will not, the potentially greater effect of MAR intervention on blood pressure of fully participating individuals may be washed out by the smaller effect of those who will not comply. To account for this, as a secondary analysis, we will conduct "instrumental variable" (IV) analyses by jointly running two regression models: a regression model for predicting urinary sodium excretion by drinking water salinity, and a regression model for blood pressure prediction given the urinary sodium excretion. The assumption of instrumental variable analyses is fulfilled in our context as the exogenous variable (instrumental variable) can only affect outcome variable (blood pressure) through influencing the endogenous variable (urinary sodium excretion).[94] If MAR water has lower-salinity than other water sources and if participants always drink MAR water, their urinary sodium excretion will be low compared to those who will not fully participate.

For peer review only - http://bmjopen.bmj.com/site/about/guidelines.xhtml

Acknowledgements: The study has been funded by Wellcome Trust, UK, Our Planet, Our Health Award. Wellcome Trust reviewed and approved the design as the condition of providing funding but will not have a role in data collection, data analysis, data interpretation, or writing of the report. The study was implemented by International Centre for Diarrhoeal Disease Research, Bangladesh (icddr,b). icddr,b acknowledges with gratitude the commitment of Wellcome Trust, UK to its research efforts. icddr,b is also grateful to the Governments of Bangladesh, Canada, Sweden and the UK for providing core/unrestricted support. Dr. Gribble is supported by funding from the National Institute for Environmental Health Sciences to the Emory Health and Research Exposome Center (P30 ES019776). The authors would like to thank Dr. Karla Hemming, Institute of Applied Health Research, University of Birmingham for her input in design and sample size calculation. We are grateful to the Dhaka University/UNICEF field team for their assistance in providing preliminary data about the readiness of MAR sites.

Contributors: SPL and AMN developed the study concept. AMN, SPL, LU, KMA, and SD have developed the study design. MR, MOG, TFC and HHC reviewed the epidemiological study design. KMA, MS, WB, SS and MNU provided input in environmental and biological sample collection and analysis. HHC and MOG provided input in statistical analysis. AMN drafted and all author reviewed the manuscript.

Competing interest: None

Funding: Wellcome Trust, UK (Grant # 106871/Z/15/Z).

Data sharing statement: The anonymous dataset without personal identifiers will be publicly available following publication of the primary trial results.

For peer review only - http://bmjopen.bmj.com/site/about/guidelines.xhtml

References-

1. Van Weert F, van der Gun J, Reckman J. Global overview of saline groundwater occurrence and genesis. International Groundwater Resources Assessment Centre 2009.
2. Vengosh A, Rosenthal E. Saline groundwater in Israel: its bearing on the water crisis in the country. Journal of Hydrology 1994;**156**(1):389-430.
3. Post V. Fresh and saline groundwater interaction in coastal aquifers: Is our technology ready for the problems ahead? Hydrogeology Journal 2005;**13**(1):120-23.
4. Holman IP. Climate change impacts on groundwater recharge-uncertainty, shortcomings, and the way forward? Hydrogeology journal 2006;**14**(5):637-47.
5. Ferguson G, Gleeson T. Vulnerability of coastal aquifers to groundwater use and climate change. Nature Climate Change 2012;**2**(5):342-45.
6. Mall R, Gupta A, Singh R, et al. Water resources and climate change: an Indian perspective. Current Science 2006;**90**(12):1610-26.
7. Taylor RG, Scanlon B, Döll P, et al. Ground water and climate change. Nature Climate Change 2013;**3**(4):322-29.
8. Changming L, Jingjie Y, Kendy E. Groundwater exploitation and its impact on the environment in the North China Plain. Water International 2001;**26**(2):265-72.
9. Counts HB, Donsky E. *Salt-water Encroachment Geology and Ground-water Resources of Savannah Area Georgia and South Carolina*: US Government Printing Office, 1963.
10. Barlow PM. *Ground Water in Fresh Water-salt Water Environments of the Atlantic*: Geological Survey (USGS), 2003.
11. McGranahan G, Balk D, Anderson B. The rising tide: assessing the risks of climate change and human settlements in low elevation coastal zones. Environment and urbanization 2007;**19**(1):17-37.
12. Villholth KG. Integrated groundwater use and management in vulnerable coastal zones of Asia-Pacific. Groundwater in the Coastal Zones of Asia-Pacific: Springer, 2013:317-42.
13. Morris BL, Lawrence AR, Chilton P, et al. Groundwater and its susceptibility to degradation: a global assessment of the problem and options for management. 2003.
14. Dillon P. Future management of aquifer recharge. Hydrogeology journal 2005;**13**(1):313-16.
15. Bouwer H. Artificial recharge of groundwater: systems, design, and management. Hydraulic design handbook McGraw-Hill, New York 1999:24.1-24.44.
16. Ahmed K, Sultana S, Groen K, et al. DEVELOPMENT OF OPTIMAL DESIGN OF AQUIFER STORAGE AND RECOVERY FOR PROVIDING SAFE WATER TO VULNERABLE COMMUNITIES OF COASTAL BANGLADESH. International conference on Climate Change in relation to Water and Environment (I3CWE-2015). Department of Civil Engineering, DUET - Gazipur, Bangladesh, 2015:1-8.
17. Dillon P, Toze S, Page D, et al. Managed aquifer recharge: rediscovering nature as a leading edge technology. Water Science and Technology 2010;**62**(10):2338.
18. Groundwater and Climate Change: Challenges and Possibilities 2008 [Available from: http://www.geus.dk/program-areas/water/denmark/rapporter/groundwater_and_%20climate_change_071108.pdf.
19. Allison MA, Khan SR, Goodbred SL, et al. Stratigraphic evolution of the late Holocene Ganges-Brahmaputra lower delta plain. Sedimentary Geology 2003;**155**(3-4):317-42.
20. Mirza MMQ. Diversion of the Ganges water at Farakka and its effects on salinity in Bangladesh. Environmental management 1998;**22**(5):711-22.
21. Karim MR. Brackish-water shrimp cultivation threatens permanent damage to coastal agriculture in Bangladesh. Environment and livelihoods in tropical coastal zones: managing agriculture-fishery-aquaculture conflicts 2006;**2**:61-71.

For peer review only - http://bmjopen.bmj.com/site/about/guidelines.xhtml

22. Mahmuduzzaman M, Ahmed ZU, Nuruzzaman A, et al. Causes of Salinity Intrusion in Coastal Belt of Bangladesh. International Journal of Plant Research 2014;**4**(4A):8-13.
23. Bahar MM, Reza MS. Hydrochemical characteristics and quality assessment of shallow groundwater in a coastal area of Southwest Bangladesh. Environmental Earth Sciences 2010;**61**(5):1065-73.
24. Zahid A, Jahan K, Ali MH, et al. Distribution of Groundwater Salinity and Its Seasonal Variability in the Coastal Aquifers of Bengal Delta, in: Zahid et al. (Eds.), Impact of Climate Change on Socio-economic Conditions of Bangladesh. German Academic Exchange Service (DAAD) 2013:170-93.
25. Changes of the seasonal salinity distribution at the Sundarbans coast due to impact of climate change. 4th International Conference on Water & Flood Management (ICWFM); 2013.
26. Islam MA, Sakakibara H, Karim MR, et al. Potable water scarcity: options and issues in the coastal areas of Bangladesh. Journal of water and health 2013;**11**(3):532-42.
27. Acacia Water DU. Action reserach: Creating fresh water bubbles in Brackish aquifer 2011 [Available from: http://www.google.com.bd/url?sa=t&rct=j&q=creating+Fresh+water+bubbles+in+Brackish+aquifers&source=web&cd=1&cad=rja&ved=0CCcQFjAA&url=http%3A%2F%2Facaciawater.com%2Fsite%2Fmedia%2Fprojecten%2FFlyer_MAR_BD.pdf&ei=Q6gHUZrHDKe0iQfdjoCADg&usg=AFQjCNHc5fhWh5Nk2v-jzz0s6PTsztFAqg&bvm=bv.41524429,d.aGc.
28. UNICEF. Underground fresh water storage A practical solution to increase water security in saline deltas 2014 [Available from: http://www.spaarwater.com/content/27227/download/clnt/57128_Underground_fresh_water_storage_-_a_practical_solution_to_increase_water_security_in_saline_deltas.pdf.
29. Sultana S, Ahmed K, Mahtab-Ul-Alam S, et al. Low-Cost Aquifer Storage and Recovery: Implications for Improving Drinking Water Access for Rural Communities in Coastal Bangladesh. Journal of Hydrologic Engineering 2014.
30. Alderman MH. Salt, blood pressure, and human health. Hypertension 2000;**36**(5):890-3.
31. Cutler JA, Follmann D, Allender PS. Randomized trials of sodium reduction: an overview. Am J Clin Nutr 1997;**65**(2 Suppl):643S-51S.
32. Midgley JP, Matthew AG, Greenwood CM, et al. Effect of reduced dietary sodium on blood pressure: a meta-analysis of randomized controlled trials. JAMA 1996;**275**(20):1590-7.
33. Strazzullo P, D'Elia L, Kandala N-B, et al. Salt intake, stroke, and cardiovascular disease: meta-analysis of prospective studies. Bmj 2009;**339**.
34. Scheelbeek PF, Chowdhury MA, Haines A, et al. High concentrations of sodium in drinking water and raised blood pressure in coastal deltas affected by episodic seawater inundations. The Lancet Global Health 2016;**4**:S18.
35. Khan AE, Scheelbeek PFD, Shilpi AB, et al. Salinity in Drinking Water and the Risk of (Pre) Eclampsia and Gestational Hypertension in Coastal Bangladesh: A Case-Control Study. 2014.
36. Khan AE, Ireson A, Kovats S, et al. Drinking Water Salinity and Maternal Health in Coastal Bangladesh: Implications of Climate Change. Environ Health Perspect 2011.
37. Scheelbeek PF, Khan AE, Mojumder S, et al. Drinking Water Sodium and Elevated Blood Pressure of Healthy Pregnant Women in Salinity-Affected Coastal Areas. Hypertension 2016;**68**(2):464-70.
38. Brown MA, Lindheimer MD, de Swiet M, et al. The classification and diagnosis of the hypertensive disorders of pregnancy: statement from the International Society for the Study of Hypertension in Pregnancy (ISSHP). Hypertension in pregnancy 2001;**20**(1):ix-xiv.
39. Dasgupta S, Kamal FA, Khan ZH, et al. River salinity and climate change: evidence from coastal Bangladesh. World Bank Policy Research Working Paper 2014(6817).
40. Mozaffarian D, Fahimi S, Singh GM, et al. Global sodium consumption and death from cardiovascular causes. New England Journal of Medicine 2014;**371**(7):624-34.

For peer review only - http://bmjopen.bmj.com/site/about/guidelines.xhtml

41. Khan AE, Ireson A, Kovats S, et al. Drinking Water Salinity and Maternal Health in Coastal Bangladesh: Implications of Climate Change. Environ Health Perspect.
42. Mente A, O'Donnell M, Rangarajan S, et al. Associations of urinary sodium excretion with cardiovascular events in individuals with and without hypertension: a pooled analysis of data from four studies. The Lancet 2016;**388**(10043):465-75.
43. Kinniburgh D, Smedley P. Arsenic contamination of groundwater in Bangladesh. Kinniburgh, D G and Smedley, P L (Editors), British Geological Survey Report WC/00/19, British Geological Survey, Keyworth. British Geological Survey 2001.
44. Sauvant M-P, Pepin D. Drinking water and cardiovascular disease. Food and chemical toxicology 2002;**40**(10):1311-25.
45. Marx A, Neutra RR. Magnesium in drinking water and ischemic heart disease. Epidemiologic reviews 1997;**19**(2):258-72.
46. BiÖrck G, BostrÖm H, WidstrÖm A. On the Relationship Between Water Hardness and Death Rate in Cardiovascular Diseases1. Acta medica Scandinavica 1965;**178**(2):239-52.
47. He FJ, MacGregor GA. Effect of longer-term modest salt reduction on blood pressure. Cochrane Database Syst Rev 2004;**3**.
48. Organization WH. *Guideline: Sodium intake for adults and children*: World Health Organization, 2012.
49. Graudal NA, Hubeck-Graudal T, Jurgens G. Effects of low sodium diet versus high sodium diet on blood pressure, renin, aldosterone, catecholamines, cholesterol, and triglyceride. Cochrane Database Syst Rev 2011;**11**.
50. He FJ, Li J, MacGregor GA. Effect of longer term modest salt reduction on blood pressure: Cochrane systematic review and meta-analysis of randomised trials. 2013.
51. He F, MacGregor G. Effect of modest salt reduction on blood pressure: a meta-analysis of randomized trials. Implications for public health. Journal of human hypertension 2002;**16**(11):761-70.
52. De Allegri M, Pokhrel S, Becher H, et al. Step-wedge cluster-randomised community-based trials: an application to the study of the impact of community health insurance. Health Res Policy Syst 2008;**6**:10.
53. UNICEF B. Bangladesh National Drinking Water Quality Survey of 2009 2009 [Available from: https://www.unicef.org/bangladesh/BNDWQS_2009_web.pdf.
54. Ministry of Environment and Forest, Government of the People’s Republic of Bangladesh. The Environment Conservation Rules, 1997 [Available from: http://extwprlegs1.fao.org/docs/pdf/bgd19918.pdf.
55. Luft FC, Miller JZ, Grim CE, et al. Salt sensitivity and resistance of blood pressure. Age and race as factors in physiological responses. Hypertension 1991;**17**(1 Suppl):I102.
56. Orchard T, Rodgers M, Hedley A, et al. Changes in blood lipids and blood pressure during adolescence. Br Med J 1980;**280**(6231):1563-67.
57. Fujita T. Mechanism of salt-sensitive hypertension: focus on adrenal and sympathetic nervous systems. Journal of the American Society of Nephrology 2014;**25**(6):1148-55.
58. BBS. POPULATION AND HOUSING CENSUS 2011 [Available from: http://203.112.218.66/WebTestApplication/userfiles/Image/BBS/Socio_Economic.pdf.
59. NIPORT. Bangladesh Demographic and Health Survey: Preliminary Report. Dhaka, 2011.
60. Khan SH, Talukder SM, Zaman MM. Non-communicable Disease Prevention Intervention in Production Sector of Bangladesh: Findings and Lessons Learnt. 13th ASCON. icddr,b, Dhaka, 2011.
61. Hussey MA, Hughes JP. Design and analysis of stepped wedge cluster randomized trials. Contemporary clinical trials 2007;**28**(2):182-91.

For peer review only - http://bmjopen.bmj.com/site/about/guidelines.xhtml

62. Hemming K, Taljaard M. Sample size calculations for stepped wedge and cluster randomised trials: a unified approach. Journal of clinical epidemiology 2016;**69**:137-46.
63. McLean RM. Measuring population sodium intake: a review of methods. Nutrients 2014;**6**(11):4651-62.
64. Brown IJ, Dyer AR, Chan Q, et al. Estimating 24-hour urinary sodium excretion from casual urinary sodium concentrations in western populations the INTERSALT study. American journal of epidemiology 2013;**177**(11):1180-92.
65. Bednarski A, Stolarz-Skrzypek K, Kielbasa G, et al. Pulse pressure in relation to 24-hour urinary sodium excretion in a sample of high-salt intake population. Artery Research 2015;**12**:5.
66. Staessen J, Bulpitt CJ, Thijs L, et al. Pulse Rate and Sodium Intake Interact to Determine Blood Pressure A Population Study. American journal of hypertension 1991;**4**(2 Pt 1):107-12.
67. Weir MR, Dengel DR, Behrens MT, et al. Salt-induced increases in systolic blood pressure affect renal hemodynamics and proteinuria. Hypertension 1995;**25**(6):1339-44.
68. Du Cailar G, Mimran A. Non-pressure-related effects of dietary sodium. Current hypertension reports 2009;**11**(1):12-17.
69. Cheung B, Ho S, Cheung A, et al. Diastolic blood pressure is related to urinary sodium excretion in hypertensive Chinese patients. QJM 2000;**93**(3):163-68.
70. Rapsomaniki E, Timmis A, George J, et al. Blood pressure and incidence of twelve cardiovascular diseases: lifetime risks, healthy life-years lost, and age-specific associations in 1· 25 million people. The Lancet 2014;**383**(9932):1899-911.
71. Kramer H, Jacobs DR, Bild D, et al. Urine albumin excretion and subclinical cardiovascular disease. Hypertension 2005;**46**(1):38-43.
72. Weir MR. Microalbuminuria and cardiovascular disease. Clinical Journal of the American Society of Nephrology 2007;**2**(3):581-90.
73. Luft FC, Agrawal B. Microalbuminuria as a predictive factor for cardiovascular events. Journal of cardiovascular pharmacology 1999;**33**:S11-S15.
74. Bigazzi R, Bianchi S, Baldari D, et al. Microalbuminuria in salt-sensitive patients. A marker for renal and cardiovascular risk factors. Hypertension 1994;**23**(2):195-99.
75. Bianchi S, Bigazzi R, Campese VM. Microalbuminuria in essential hypertension: significance, pathophysiology, and therapeutic implications. American journal of kidney diseases 1999;**34**(6):973-95.
76. Agrawal B, Berger A, Wolf K, et al. Microalbuminuria screening by reagent strip predicts cardiovascular risk in hypertension. Journal of hypertension 1996;**14**(2):223-28.
77. Sarafidis PA, Bakris GL. Microalbuminuria and chronic kidney disease as risk factors for cardiovascular disease. Nephrology Dialysis Transplantation 2006;**21**(9):2366-74.
78. Diercks G, Van Boven A, Hillege H, et al. Microalbuminuria is independently associated with ischaemic electrocardiographic abnormalities in a large non-diabetic population. The PREVEND (Prevention of REnal and Vascular ENdstage Disease) study. European heart journal 2000;**21**(23):1922-27.
79. Bigazzi R, Bianchi S, Nenci R, et al. Increased thickness of the carotid artery in patients with essential hypertension and microalbuminuria. Journal of human hypertension 1995;**9**(10):827-33.
80. Mykkänen L, Zaccaro DJ, O'Leary DH, et al. Microalbuminuria and carotid artery intima-media thickness in nondiabetic and NIDDM subjects The Insulin Resistance Atherosclerosis Study (IRAS). Stroke 1997;**28**(9):1710-16.
81. Pedrinelli R, Di Bello V, Catapano G, et al. Microalbuminuria is a marker of left ventricular hypertrophy but not hyperinsulinemia in nondiabetic atherosclerotic patients. Arteriosclerosis, Thrombosis, and Vascular Biology 1993;**13**(6):900-06.

For peer review only - http://bmjopen.bmj.com/site/about/guidelines.xhtml

82. Assadi F. Relation of left ventricular hypertrophy to microalbuminuria and C-reactive protein in children and adolescents with essential hypertension. Pediatric cardiology 2008;**29**(3):580-84.
83. Campese VM, Bianchi S, Bigazzi R. Association between hyperlipidemia and microalbuminuria in essential hypertension. Kidney International 1999;**56**:S10-S13.
84. Jensen JS, Borch-Johnsen K, Jensen G, et al. Atherosclerotic risk factors are increased in clinically healthy subjects with microalbuminuria. Atherosclerosis 1995;**112**(2):245-52.
85. Klausen K, Borch-Johnsen K, Feldt-Rasmussen B, et al. Very low levels of microalbuminuria are associated with increased risk of coronary heart disease and death independently of renal function, hypertension, and diabetes. Circulation 2004;**110**(1):32-35.
86. Currie G, Delles C. Proteinuria and its relation to cardiovascular disease. International journal of nephrology and renovascular disease 2013;**7**:13-24.
87. Swift PA, Markandu ND, Sagnella GA, et al. Modest salt reduction reduces blood pressure and urine protein excretion in black hypertensives A randomized control trial. Hypertension 2005;**46**(2):308-12.
88. Leeman L, Fontaine P. Hypertensive disorders of pregnancy. American family physician 2008;**78**(1):93-100.
89. Ostchega Y, Zhang G, Sorlie P, et al. Blood Pressure Randomized Methodology Study Comparing Automatic Oscillometric and Mercury Sphygmomanometer Devices: National Health and Nutrition Examination Survey, 2009–2010. National Health Statistics Reports, 2012.
90. Pickering T, Hall J, Appel L, et al. Blood pressure measurement in humans: A statement for professionals from the subcommittee of professional and public education of the American Heart Association Council on High Blood Pressure Research. J Am Heart Assoc 2005;**45**:142-61.
91. Giorgini P, Weder AB, Jackson EA, et al. A review of blood pressure measurement protocols among hypertension trials: implications for “evidence-based” clinical practice. Journal of the American Society of Hypertension 2014;**8**(9):670-76.
92. Schwartz S, Gatto NM, Campbell UB. Extending the sufficient component cause model to describe the Stable Unit Treatment Value Assumption (SUTVA). Epidemiologic Perspectives & Innovations 2012;**9**(1):3.
93. VanderWeele TJ. Concerning the consistency assumption in causal inference. Epidemiology 2009;**20**(6):880-83.
94. Arellano M, Bover O. Another look at the instrumental variable estimation of error-components models. Journal of econometrics 1995;**68**(1):29-51.

For peer review only

For peer review only - http://bmjopen.bmj.com/site/about/guidelines.xhtml

Table 1: Study outcomes including normal ranges of blood pressure and biomarkers

Outcomes	Study population		Measures	Normal range/calculation
	≥ 20 years old	Pregnant women		
Primary outcome: Systolic blood pressure	Yes	Yes	Systolic blood pressure	90 -140 mmHg
Secondary outcomes: Diastolic blood pressure	Yes	Yes	Diastolic blood pressure	60 -90 mmHg
Mean arterial pressure	Yes	Yes	Mean arterial pressure	Diastolic blood pressure + 1/3 systolic blood pressure
Pulse pressure	Yes	Yes	Pulse pressure	Systolic blood pressure – diastolic blood pressure
Tertiary outcomes: Urinary sodium excretion	Yes	Yes	Urinary sodium-creatinine ratio	40-220 mEq/L/24 hours
Proteinuria	Yes	Yes	Urinary protein-creatinine ratio	< 0.11 mg/mg

For peer review only - http://bmjopen.bmj.com/site/about/guidelines.xhtml

Table 2: Key variables to be used for assessing the health impact of access to MAR water

**Outcomes**	**Exposures**	**Confounders**	**Co-variates**	**Moderators**
Systolic blood pressure	Access to MAR water	Sex	Age	Drinking/cooking from another water sources
Diastolic blood pressure	Drinking water salinity	Socio-economic status	Weight	
Pulse pressure	Cooking water salinity	Education	Height	
Mean arterial pressure			Waist circumference	
	Drinking water sources			
	Cooking water sources			
Urinary total protein			Mid upper arm circumference	
Urinary sodium excretion				
			Adding table salt	
			Adding salt for cooking	
			Exercise level	
			Smoking status	
			Use of betel nuts	
			Stress	
			Diet	
			Urinary creatinine	

For peer review only

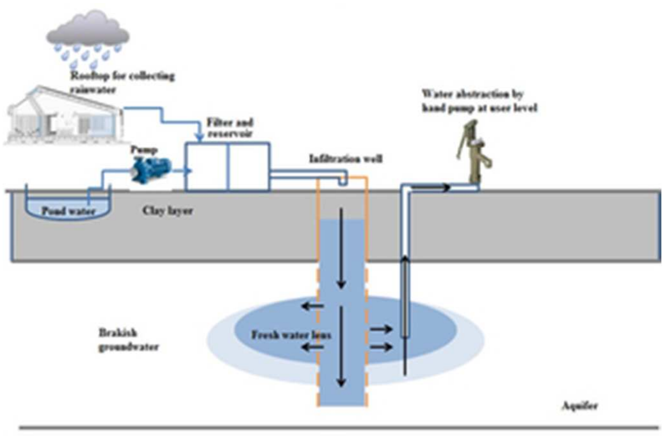

Figure 1: Schematic diagram of the MAR systems in southwest coastal Bangladesh

27x18mm (300 x 300 DPI)

For peer review only

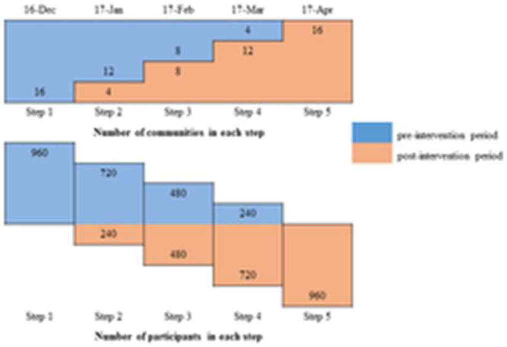

Figure 3: Timeline of blood pressure measurement with number of MAR communities and participants in pre-intervention (blue color) and post-intervention (brown color) time period in different steps

22x15mm (300 x 300 DPI)

For peer review only

SPIRIT 2013 Checklist: Recommended items to address in a clinical trial protocol and related documents*

Section/item	Item No	Description	Addressed on page number
**Administrative information**			
Title	1	Descriptive title identifying the study design, population, interventions, and, if applicable, trial acronym	Page 1
Trial registration	2a	Trial identifier and registry name. If not yet registered, name of intended registry	Page 3
	2b	All items from the World Health Organization Trial Registration Data Set	NA
Protocol version	3	Date and version identifier	MAR design paper_30 May 2017.doc
Funding	4	Sources and types of financial, material, and other support	Page 22
Roles and responsibilities	5a	Names, affiliations, and roles of protocol contributors	Page 22
	5b	Name and contact information for the trial sponsor	Page 22
	5c	Role of study sponsor and funders, if any, in study design; collection, management, analysis, and interpretation of data; writing of the report; and the decision to submit the report for publication, including whether they will have ultimate authority over any of these activities	Page 22, 18

For peer review only - http://bmjopen.bmj.com/site/about/guidelines.xhtml

	5d	Composition, roles, and responsibilities of the coordinating centre, steering committee, endpoint adjudication committee, data management team, and other individuals or groups overseeing the trial, if applicable (see Item 21a for data monitoring committee)	NA
**Introduction**			
Background and rationale	6a	Description of research question and justification for undertaking the trial, including summary of relevant studies (published and unpublished) examining benefits and harms for each intervention	Page 4 —7
	6b	Explanation for choice of comparators	Page 8
Objectives	7	Specific objectives or hypotheses	Page 7
Trial design	8	Description of trial design including type of trial (eg, parallel group, crossover, factorial, single group), allocation ratio, and framework (eg, superiority, equivalence, noninferiority, exploratory)	Page 8
**Methods: Participants, interventions, and outcomes**			
Study setting	9	Description of study settings (eg, community clinic, academic hospital) and list of countries where data will be collected. Reference to where list of study sites can be obtained	Page 8
Eligibility criteria	10	Inclusion and exclusion criteria for participants. If applicable, eligibility criteria for study centres and individuals who will perform the interventions (eg, surgeons, psychotherapists)	Page 9
Interventions	11a	Interventions for each group with sufficient detail to allow replication, including how and when they will be administered	Page 8 —10
	11b	Criteria for discontinuing or modifying allocated interventions for a given trial participant (eg, drug dose change in response to harms, participant request, or improving/worsening disease)	NA
	11c	Strategies to improve adherence to intervention protocols, and any procedures for monitoring adherence (eg, drug tablet return, laboratory tests)	Page 9, 10
	11d	Relevant concomitant care and interventions that are permitted or prohibited during the trial	NA

For peer review only - http://bmjopen.bmj.com/site/about/guidelines.xhtml

Outcomes	12	Primary, secondary, and other outcomes, including the specific measurement variable (eg, systolic blood pressure), analysis metric (eg, change from baseline, final value, time to event), method of aggregation (eg, median, proportion), and time point for each outcome. Explanation of the clinical relevance of chosen efficacy and harm outcomes is strongly recommended	Page 13 — 15
Participant timeline	13	Time schedule of enrolment, interventions (including any run-ins and washouts), assessments, and visits for participants. A schematic diagram is highly recommended (see Figure)	Figure 3
Sample size	14	Estimated number of participants needed to achieve study objectives and how it was determined, including clinical and statistical assumptions supporting any sample size calculations	Page 10,11
Recruitment	15	Strategies for achieving adequate participant enrolment to reach target sample size	Page 9, 10
**Methods: Assignment of interventions (for controlled trials)**			
Allocation:			
Sequence generation	16a	Method of generating the allocation sequence (eg, computer-generated random numbers), and list of any factors for stratification. To reduce predictability of a random sequence, details of any planned restriction (eg, blocking) should be provided in a separate document that is unavailable to those who enrol participants or assign interventions	Page 9
Allocation concealment mechanism	16b	Mechanism of implementing the allocation sequence (eg, central telephone; sequentially numbered, opaque, sealed envelopes), describing any steps to conceal the sequence until interventions are assigned	NA
Implementation	16c	Who will generate the allocation sequence, who will enrol participants, and who will assign participants to interventions	Page 9
Blinding (masking)	17a	Who will be blinded after assignment to interventions (eg, trial participants, care providers, outcome assessors, data analysts), and how	NA
	17b	If blinded, circumstances under which unblinding is permissible, and procedure for revealing a participant’s allocated intervention during the trial	NA
**Methods: Data collection, management, and analysis**			

For peer review only - http://bmjopen.bmj.com/site/about/guidelines.xhtml

Data collection methods	18a	Plans for assessment and collection of outcome, baseline, and other trial data, including any related processes to promote data quality (eg, duplicate measurements, training of assessors) and a description of study instruments (eg, questionnaires, laboratory tests) along with their reliability and validity, if known. Reference to where data collection forms can be found, if not in the protocol	Page 12 — 16
	18b	Plans to promote participant retention and complete follow-up, including list of any outcome data to be collected for participants who discontinue or deviate from intervention protocols	Page 9,10
Data management	19	Plans for data entry, coding, security, and storage, including any related processes to promote data quality (eg, double data entry; range checks for data values). Reference to where details of data management procedures can be found, if not in the protocol	Page 16
Statistical methods	20a	Statistical methods for analysing primary and secondary outcomes. Reference to where other details of the statistical analysis plan can be found, if not in the protocol	Page 16, 17
	20b	Methods for any additional analyses (eg, subgroup and adjusted analyses)	Page 16, 17
	20c	Definition of analysis population relating to protocol non-adherence (eg, as randomised analysis), and any statistical methods to handle missing data (eg, multiple imputation)	Page 16, 17
**Methods: Monitoring**			
Data monitoring	21a	Composition of data monitoring committee (DMC); summary of its role and reporting structure; statement of whether it is independent from the sponsor and competing interests; and reference to where further details about its charter can be found, if not in the protocol. Alternatively, an explanation of why a DMC is not needed	Page 16
	21b	Description of any interim analyses and stopping guidelines, including who will have access to these interim results and make the final decision to terminate the trial	NA
Harms	22	Plans for collecting, assessing, reporting, and managing solicited and spontaneously reported adverse events and other unintended effects of trial interventions or trial conduct	Page 16
Auditing	23	Frequency and procedures for auditing trial conduct, if any, and whether the process will be independent from investigators and the sponsor	NA
**Ethics and dissemination**			

For peer review only - http://bmjopen.bmj.com/site/about/guidelines.xhtml

Research ethics approval	24	Plans for seeking research ethics committee/institutional review board (REC/IRB) approval	Page 17
Protocol amendments	25	Plans for communicating important protocol modifications (eg, changes to eligibility criteria, outcomes, analyses) to relevant parties (eg, investigators, REC/IRBs, trial participants, trial registries, journals, regulators)	Page 17
Consent or assent	26a	Who will obtain informed consent or assent from potential trial participants or authorised surrogates, and how (see Item 32)	Page 9, 12, 17
	26b	Additional consent provisions for collection and use of participant data and biological specimens in ancillary studies, if applicable	Page 17
Confidentiality	27	How personal information about potential and enrolled participants will be collected, shared, and maintained in order to protect confidentiality before, during, and after the trial	Page 17
Declaration of interests	28	Financial and other competing interests for principal investigators for the overall trial and each study site	Page 22
Access to data	29	Statement of who will have access to the final trial dataset, and disclosure of contractual agreements that limit such access for investigators	Page 22
Ancillary and post-trial care	30	Provisions, if any, for ancillary and post-trial care, and for compensation to those who suffer harm from trial participation	NA
Dissemination policy	31a	Plans for investigators and sponsor to communicate trial results to participants, healthcare professionals, the public, and other relevant groups (eg, via publication, reporting in results databases, or other data sharing arrangements), including any publication restrictions	Page 18
	31b	Authorship eligibility guidelines and any intended use of professional writers	
	31c	Plans, if any, for granting public access to the full protocol, participant-level dataset, and statistical code	Page 22
**Appendices**			
Informed consent materials	32	Model consent form and other related documentation given to participants and authorised surrogates	Supplemental Information 1A & 1B

For peer review only - http://bmjopen.bmj.com/site/about/guidelines.xhtml

Biological specimens	33	Plans for collection, laboratory evaluation, and storage of biological specimens for genetic or molecular analysis in the current trial and for future use in ancillary studies, if applicable	NA

*It is strongly recommended that this checklist be read in conjunction with the SPIRIT 2013 Explanation & Elaboration for important clarification on the items. Amendments to the protocol should be tracked and dated. The SPIRIT checklist is copyrighted by the SPIRIT Group under the Creative Commons "Attribution-NonCommercial-NoDerivs 3.0 Unported" license.

For peer review only

Supplemental Information 1A: Consent from household head

Protocol Title: Health impacts of a climate change adaptation strategy to address drinking-water salinity in coastal Bangladesh

Investigator's name: Dr. Md. Mahbubur Rahman

Organization: **International Centre for Diarrhoeal Disease Research, Bangladesh (ICDDR,B)**

Purpose of the research

Hello (Assalamualaikum/Nomoshkar). My name is ________ and I work with the ICDDR,B (Cholera Hospital) in Dhaka. You are aware that a Managed aquifer recharge scheme has already been installed in your community that would like reduce the salinity problem in your drinking water. You may also know that drinking saline water is associated with hypertension. We will conduct a pilot study to understand the mean blood pressure of the household members who are drinking water from MAR.

Background (brief introduction of the issue and the need for/ importance of the research)
Salinity in drinking water is a big problem in your community and in many other communities in the south-western coastal region of Bangladesh. Previous research has demonstrated that drinking saline water caused high blood pressure among the adult population and gestational hypertension among the pregnant women. Hypertension risk many other cardiovascular diseases and gestational hypertension also causes adverse pregnancy and fetal outcomes. Managed aquifer recharge (MAR) is a promising sustainable intervention that can reduce salinity in groundwater. Moreover, MAR will not be affected by the cyclone and tidal surges. There is a potential to scale-up MAR across the south-western coastal region if we can demonstrate that drinking MAR water provide health benefits and do not pose any risk to human health. Now we would like to conduct a pilot study where we will measure the mean blood pressure of your household members and the salt concentration of their urine.

Why invited to participate in the study?
You're invited to participate in our study because your household is located in such an area where a new MAR site has been installed. Rain water and pond water is being infiltrated into the groundwater aquifers in this MAR scheme to lower the groundwater salinity. Currently, your household may be using MAR water or other water sources for drinking and cooking purposes. Since your household is among one of the households under the MAR catchment area, we are inviting you to participate in this study.

Methods and procedures
If your household decides to participate, we make two visits to your households. The study duration will be around four months. We will collect information from all the available household members $\geq$20 years old. In each visit, we will measure the blood pressure of all the $\geq$20 years household members and may also measure their 24 hour ambulatory arterial blood pressure by strapping a device in their arms for next 24 hours. We will collect about 20 ml urine

For peer review only - http://bmjopen.bmj.com/site/about/guidelines.xhtml

from all the household members as spot urine and will provide a large bag to collect the total amount of urine during the next 24 hours time. We will measure the total protein, creatinine, in urine that will provide important information about your kidney health. In the first visit, the research staff will collect the demographic and socio-economic information, smoking and diseases history, and measure height of available household members. We will also collect 5 ml blood from the participant to assess the genetic marker to understand whether their gene indicates that reduction of salt intake can reduce blood pressure of your family members. Samples will be stored for up to 5 years after the end of the study because we want to take advantage of expected future advances in characterizing genetic markers and other parameters relevant to the salt sensitivity of hypertension. At the end of this time, the samples will be destroyed and a certificate of their destruction will be provided to iccdr,b. If you wish to withdraw your samples from the study earlier you may contact the investigators.

In addition, we will collect information about your drinking water sources during each visit and will collect your stored drinking water samples for measuring salinity and sodium concentration in water.

Risk and benefits

There is no more than minimal risk involved in this study. Your household members may feel bit discomfort during blood pressure measurement, blood and urine sample collection, but these will provide important health messages about your household member's health. Hypertension is very prevalent in Bangladesh and it remains undetected to many people. By participating in this study, your household members will have a screening for hypertension.

There will be also no social harm to you and the rest of the households in this village by your participation in this study. During the visit day, your household need to spend time (~90 minutes) from your daily life for study purposes. There is no direct monetary compensation benefit for participating in this study. However, if drinking MAR water seems to provide health benefits, the study result will help to scale MAR across coastal Bangladesh that many people will have access to safe water with low salinity.

Privacy, anonymity and confidentiality

We will maintain the confidentiality of what information you give us and we will not disclose your identity when we write our reports. We will only use the collective information for the purpose of this study, and we will not use your name in sharing and publishing the results of this study. We expect the steps we take will keep all of your information confidential.

Future use of information

The information collected from this study may be shared with other researchers if needed, but we will strictly maintain your confidentiality and privacy. In the future, we may wish to perform additional tests on the urine and blood sample that will be collected. The samples will be stored at icddr,b.

Right not to participate and withdraw

Taking part in the study is completely voluntary. You may choose not to answer any or all of the questions that will be asked about your household or your behavior. You can drop out of this

For peer review only

study at any time during the interview or anytime during the study period. You have the right to refuse participation in this study.

Principle of compensation

You need not to pay us to take part in this study, and similarly we will not pay you money for attending in the study. You will also be provided best possible, free treatment, for research related injuries.

[Note: Payment for loss of earning of the study participants may be considered in case the participants require extended hospitalization or confinement only for the purpose of the research and/or reimbursement of cost of transportation for participating in the study. However, the amount should be equivalent to the loss and not so high that the offer might induce (influence/bias judgment) participation in the study.

Answering your questions/ Contact persons

If you have any question, you can ask me any time. If you have additional questions about the survey, you may contact: Dr. Solaiman Doza ,Infectious Disease Division, icddr,b, Mohakhali, Dhaka 1212. Phone: +880-2-9827001-10, ext. 4010

If you have questions about your rights as a participant of this research study, or if you think some harm has been done to you because of the survey, you may contact the ICDDR,B IRB: M. A. Salam Khan, IRB Secretariat, phone: 9827084 or PABX 9827001-10 ext. 3206

If you agree to our proposal of enrolling you/your patient in our study, please indicate that by putting your signature or your left thumb impression at the specified space below

Thank you for your cooperation

____________________________________ ____________________
Signature or left thumb impression Date

____________________________________ ____________________
Signature or left thumb impression of the witness Date

____________________________________ ____________________
Signature of the PI or his/her representative Date

(NOTE: In case of representative of the PI, she/he shall put her/his full name and designation and then sign)

For peer review only

Supplemental Information 1B: Consent from ≥20 year household members

Protocol Title: Health impacts of a climate change adaptation strategy to address drinking-water salinity in coastal Bangladesh

Investigator's name: Dr. Md. Mahbubur Rahman

Organization: **International Centre for Diarrhoeal Disease Research, Bangladesh (ICDDR,B)**

Purpose of the research

Hello (Assalamualaikum/Nomoshkar). My name is ________ and I work with the ICDDR,B (Cholera Hospital) in Dhaka. You are aware that a Managed aquifer recharge scheme has already been installed in your community that would like reduce the salinity problem in your drinking water. You may also know that drinking saline water is associated with hypertension. We will conduct a pilot study to understand the mean blood pressure of the household members who are drinking water from MAR .

Background (brief introduction of the issue and the need for/ importance of the research)
Salinity in drinking water is a big problem in your community and in many other communities in the south-western coastal region of Bangladesh. Previous research has demonstrated that drinking saline water caused high blood pressure among the adult population and gestational hypertension among the pregnant women. Hypertension risk many other cardiovascular diseases and gestational hypertension also causes adverse pregnancy and fetal outcomes. Managed aquifer recharge (MAR) is a promising sustainable intervention that can reduce salinity in groundwater. Moreover, MAR will not be affected by the cyclone and tidal surges. There is a potential to scale-up MAR across the south-western coastal region if we can demonstrate that drinking MAR water provide health benefits and do not pose any risk to human health. Now we would like to conduct a pilot study where we will measure your mean blood pressure and the salt concentration of urine.

Why invited to participate in the study?
You're invited to participate in our study because your household is located in such an area where a new MAR site has been installed. Rain water and pond water is being infiltrated into the groundwater aquifers in this MAR scheme to lower the groundwater salinity. Currently, you may be using MAR water or other water sources for drinking and cooking purposes. We are inviting all the household members ≥20 years old in this MAR scheme catchment area to routinely measure blood pressure and collect urine samples. Since your age is >20 (or 20) years, your participation is very important for the study.

Methods and procedures
If you decide to participate, we will make two visits to measure your blood pressure, collect urine and blood. We may also measure your 24 hour ambulatory arterial blood pressure by strapping a device in your arm for 24 hours. We will collect about 20 ml urine as spot urine and

For peer review only

will provide a large bag to collect the total amount of urine during the 24 hours time. We will measure the total protein, creatinine, in urine that will provide important information about your kidney health. In the first visit, the research staff will collect the demographic and socio-economic information, smoking and diseases history, and measure height of available household members. We will also collect 5 ml of your blood to assess the genetic marker to understand whether your gene indicates that reduction of salt intake can reduce blood pressure of your family members.

So, if you agree, we may also invite you for some further discussions. We would like to record this sessions in a tape recorder as it is very difficult to write all the conversation in a note pad. We would also like to take some notes from this session. This session will last about an hour and half.

Risk and benefits

There is no more than minimal risk involved in this study. You may feel bit discomfort during blood pressure measurement, and urine sample collection, but these will provide important health messages about your health. Hypertension is very prevalent in Bangladesh and it remains undetected to many people. By participating in this study, you will be screened for hypertension.

There will be also no social harm to you and the rest of the households in this village by your participation in this study. During the visit day, you may need to spend time (~90 minutes) from your daily life for study purposes. There is no direct monetary compensation benefit for participating in this study. However, if drinking MAR water seems to provide health benefits, the study result will help to scale MAR across coastal Bangladesh that many people will have access to safe water with low salinity.

Privacy, anonymity and confidentiality

We will maintain the confidentiality of what information you give us and we will not disclose your identity when we write our reports. We will only use the collective information for the purpose of this study, and we will not use your name in sharing and publishing the results of this study. We expect the steps we take will keep all of your information confidential.

Future use of information

The information collected from this study may be shared with other researchers if needed, but we will strictly maintain your confidentiality and privacy. In the future, we may wish to perform additional tests on the urine sample that will be collected. The samples will be stored at icddr,b.

Right not to participate and withdraw

Taking part in the study is completely voluntary. You may choose not to answer any or all of the questions that will be asked about your household or your behavior. You can drop out of this study at any time during the interview or anytime during the study period. You have the right to refuse participation in this study.

Principle of compensation

For peer review only

You need not to pay us to take part in this study, and similarly we will not pay you money for attending in the study. You will also be provided best possible, free treatment, for research related injuries.

[Note: Payment for loss of earning of the study participants may be considered in case the participants require extended hospitalization or confinement only for the purpose of the research and/or reimbursement of cost of transportation for participating in the study. However, the amount should be equivalent to the loss and not so high that the offer might induce (influence/bias judgment) participation in the study.

Answering your questions/ Contact persons

If you have any question, you can ask me any time. If you have additional questions about the survey, you may contact: Dr. Solaiman Doza ,Infectious Disease Division, icddr,b, Mohakhali, Dhaka 1212. Phone: +880-2-9827001-10, ext. 4010

If you have questions about your rights as a participant of this research study, or if you think some harm has been done to you because of the survey, you may contact the ICDDR,B IRB: M. A. Salam Khan, IRB Secretariat, phone: 9827084 or PABX 9827001-10 ext. 3206

If you agree to our proposal of enrolling you/your patient in our study, please indicate that by putting your signature or your left thumb impression at the specified space below

Thank you for your cooperation

Signature or left thumb impression	Date
Signature or left thumb impression of the witness	Date
Signature of the PI or his/her representative	Date

(NOTE: In case of representative of the PI, she/he shall put her/his full name and designation and then sign

For peer review only
